# Balancing the need for seed against invasive species risks in prairie habitat restorations

**DOI:** 10.1371/journal.pone.0248583

**Published:** 2021-04-07

**Authors:** Jennifer L. Larson, Diane L. Larson, Robert C. Venette

**Affiliations:** 1 United States Geological Survey, Northern Prairie Wildlife Research Center, Minnesota Field Station, Saint Paul, Minnesota, United States of America; 2 United States Department of Agriculture–Forest Service, Northern Research Station, Saint Paul, Minnesota, United States of America; Institute for Applied Ecology, UNITED STATES

## Abstract

Adequate diversity and abundance of native seed for large-scale grassland restorations often require commercially produced seed from distant sources. However, as sourcing distance increases, the likelihood of inadvertent introduction of multiple novel, non-native weed species as seed contaminants also increases. We created a model to determine an “optimal maximum distance” that would maximize availability of native prairie seed from commercial sources while minimizing the risk of novel invasive weeds via contamination. The model focused on the central portion of the Level II temperate prairie ecoregion in the Midwest US. The median optimal maximum distance from which to source seed was 272 km (169 miles). In addition, we weighted the model to address potential concerns from restoration practitioners: 1. sourcing seed via a facilitated migration strategy (i.e., direct movement of species from areas south of a given restoration site to assist species’ range expansion) to account for warming due to climate change; and 2. emphasizing non-native, exotic species with a federal mandate to control. Weighting the model for climate change increased the median optimal maximum distance to 398 km (247 miles), but this was not statistically different from the distance calculated without taking sourcing for climate adaptation into account. Weighting the model for federally mandated exotic species increased the median optimal maximum distance only slightly to 293 km (182 miles), so practitioners may not need to adjust their sourcing strategy, compared to the original model. This decision framework highlights some potential inadvertent consequences from species translocations and provides insight on how to balance needs for prairie seed against those risks.

## Introduction

Habitat restorations are increasingly used as a means to conserve species, enhance ecosystem function, and respond to environmental changes [1]. Native plant species translocations (i.e., moving plant material from one part of a species’ range to another, primarily via seed) are one of the primary tools used to restore grassland habitats [2]. Moving seed presents a variety of concerns to restoration practitioners. For example, obtaining seed from sources close to a planned restoration site (i.e., a local provenance strategy) is often recommended to maintain local adaptation and avoid outbreeding depression in both the translocated population and nearby native populations of the same species [3]. Sourcing seed from locations with a historical climate that resembles anticipated future conditions from climate change has received recent attention as a form of climate adaptation to preserve ecosystem function [4–6]. To date, much of the academic literature regarding plant species translocations has focused on genetic concerns, and that work has resulted in the main seed sourcing strategies presented to restoration practitioners [4, 7].

Practitioners and seed commerce regulatory agencies have long recognized the risk of introducing weedy, exotic plant species to a site or a landscape via contamination of commercially produced seed [8], particularly for prairies or grasslands. The recent introduction of Palmer amaranth (*Amaranthus palmeri*) in the upper Midwest exemplifies this threat [9]. Palmer amaranth is an agricultural weed common in the southern and southwestern United States [10] that has developed resistance to several herbicides [11]. It was likely introduced to the Upper Midwest as a contaminant of native seed produced outside the region, which was brought into the region for use in prairie conservation plantings [12]. Had seed been sourced closer to the restoration sites, Palmer amaranth likely would not have entered the region through this pathway. Thus, the distance between the range of a potentially new exotic species and a restoration site substantially affects the degree of risk associated with plant translocations, via the potential for an exotic species to be a contaminant of native seed lots produced within, but then planted outside of, its current range. Indeed, many agencies recommend or require sourcing native seed from as close as possible to a restoration site, partly out of concern over the introduction of novel weeds [13–16].

Requiring that seed be sourced locally to a restoration site, while having numerous benefits, can be challenging for practitioners with respect to the diversity, abundance, and cost of native seed [e.g.,^16^, ^17^]. Ethically harvesting native seed from the wild is limited to the species already present on a local landscape and to only a fraction of the seed they produce per season [18–20]. Commercial production of native species is limited by the available knowledge of their biology [19] and to species that can be profitably grown in agricultural-like conditions [21]. Thus, while increasing the acceptable seed sourcing distance from a restoration site increases the risk of introducing a new exotic species, it also increases native seed availability (diversity and/or abundance), via an increase in the number of natural or commercial sources of seed. Good seed availability is essential for maximizing species diversity in grassland restorations, which can increase resilience and decrease invasibility [22, 23].

Here, we present the results of a decision framework that balances the increased risk of introducing new exotic species to a landscape associated with moving plant seed greater distances, with the increase in commercial seed availability that also comes with expanding the acceptable sourcing distance. First, we developed a geospatial model to identify an optimal maximum distance for sourcing native prairie species’ seed in the upper Midwest. Our model was parametrized with the locations of native plant species’ seed producers and the geographic distributions of exotic species that have the potential to invade the region. Second, we interpret the results of our model in relation to other common concerns among restoration practitioners, including risk associated with facilitated migration in sourcing based on climate change, and exotic species with a federal mandate for control. We use these concerns to provide examples of how our model recommendations could be incorporated into a practitioner’s larger seed sourcing or invasive species risk management decision-making processes. Together, these two parts create a decision framework that first uses an evidence-based tool, then provides guidance for incorporating that tool’s empirical results into the complexity of grassland restoration implementation and management. This guidance complements, but does not substitute for, seed sourcing recommendations based on genetic adaptation.

## Methods

### Model

#### Overview

**Fig 1:**
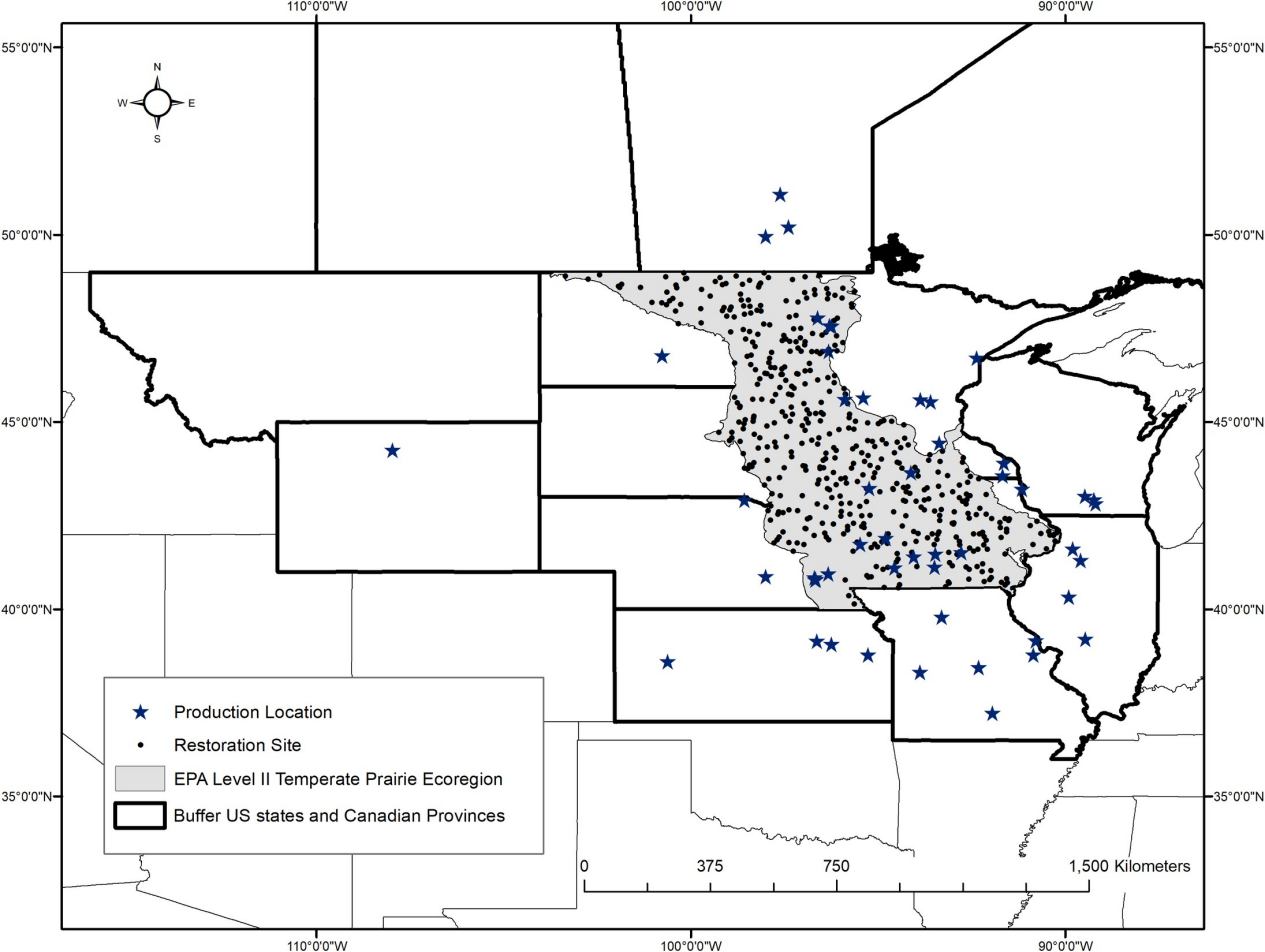
Geographic scope of the analyses. The focal region for identifying native seed production locations was delineated as a one US state or Canadian province buffer around the Level II Temperate Prairie Ecoregion within the upper Midwest USA, which is our sampling region. The map shows the final 483 hypothetical restoration sites (filled black circles) that were used in our analyses. Seed production locations and exotic species distributions were obtained for the focal region of the study. US state and county data are publicly available from the US Census Bureau and are not subject to copyright (https://www.census.gov/geographies/mapping-files/time-series/geo/carto-boundary-file.2017.html). Canadian province data are publicly available through Statistics Canada and also are not subject to copyright (https://www150.statcan.gc.ca/n1/en/catalogue/92-160-X).

**Fig 2:**
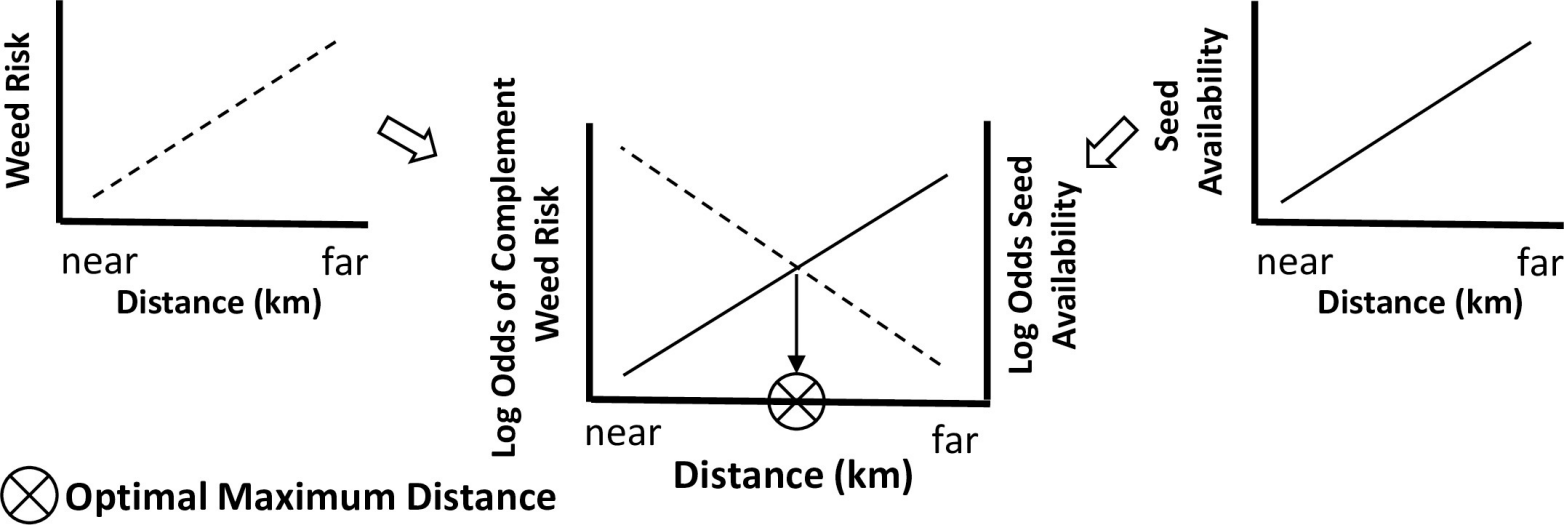
Conceptual description of the approach to determine the maximum optimal distance for sourcing seed. As the distance from a restoration site increases: 1) the total number of seed production locations available to a practitioner increases, thus the availability of native seed increases (right graphic); but 2) the total number of exotic species within that distance also increases, thus the risk of introducing a new weedy invader increases (left graphic). Our model found a balance point between seed availability and weed risk by: 1) taking the complement of the weed risk curve, 2) fitting a logistic regression to each curve, then 3) finding the distance at which the two lines intersect. We called that distance the ‘optimal maximum distance’ and define it as: the maximum distance from a restoration site for sourcing native plant species’ seed in order to minimize the risk of introducing a new exotic species while retaining as much native species’ seed availability as possible.

The goal of the model was to quantitatively balance two primary concerns that restoration practitioners consider when making seed provenance choices: the availability of native plant species seed and the risk of inadvertently introducing a new exotic plant species as a seed contaminant. To do this, we compiled two georeferenced datasets within our focal region in the upper Midwest USA and south-central Canada. The focal region for identifying native seed production locations was delineated as a one US-state or Canadian-province buffer around the five states in which our model’s sampling region was located. Our model’s sampling region was delineated as the Level II Temperate Prairie Ecoregion [24, 25] within the upper Midwest US states of Minnesota, Iowa, North Dakota, South Dakota, and Nebraska (Fig 1). We postulated that as the distance from a restoration site increases: 1) the total number of seed production locations available to a practitioner increases, thus the availability of native seed increases; but, 2) the total number of exotic species within that distance also increases; thus, the risk of introducing a new invader increases and the safety associated with that seed decreases (Figs 2 and S1). The model was parametrized with the known locations of native plant species’ seed producers and the geographic distributions of exotic species that have the potential to invade the region. Using linear approximations, the model quantitatively integrates the distance between randomly sampled hypothetical restoration sites (Fig 1) and production locations (availability) or exotic species’ range edges (risk). Our result identified an optimal maximum distance (km) within which native plant species’ seed could be sourced such that seed availability is maximized while the risk of introducing new exotic species to the region is minimized.

#### Data collection

**Fig 3:**
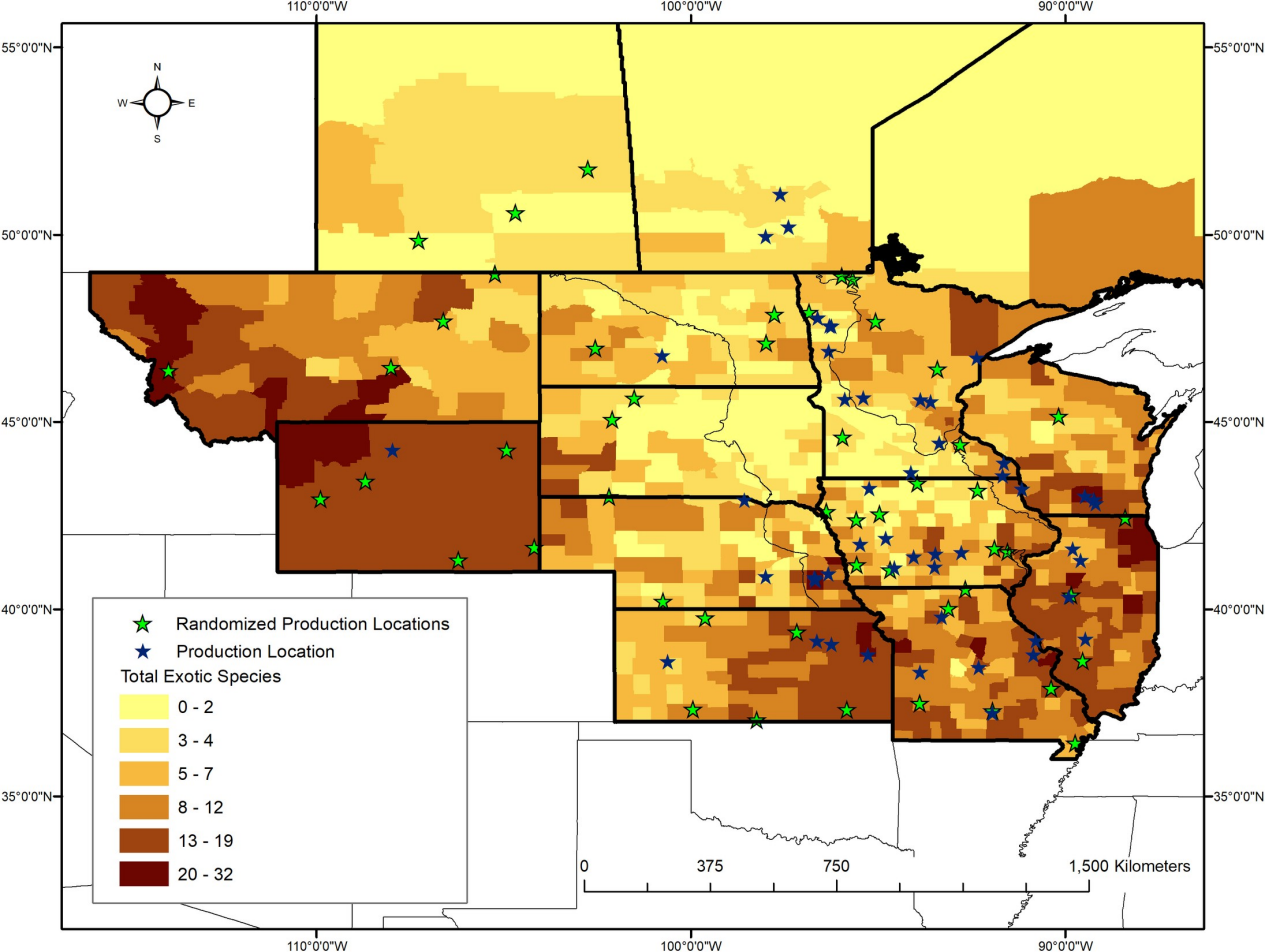
Data used to parameterize the model. The 50 production locations (filled black stars) are operated by 46 native seed production companies used in this study. Color shading by county indicates the total number of exotic species that would be new to each county within our focal region (‘new’ defined in text). The exotic species data are presented in aggregate here, however the data were used at the species level (S2 Appendix). US state and county data are publicly available from the US Census Bureau and are not subject to copyright (https://www.census.gov/geographies/mapping-files/time-series/geo/carto-boundary-file.2017.html). Canadian province data are publicly available through Statistics Canada and also are not subject to copyright (https://www150.statcan.gc.ca/n1/en/catalogue/92-160-X).

We searched ten sources (S1 Table) for native-seed production companies within our focal region. To be included in our model, a company had to grow or wild-harvest seed from native plant species that would be appropriate for a grassland-style planting (e.g., native roadside plantings, prairie restorations) in our model’s sampling area. Companies that did not produce seed or wild-harvest seed (i.e., they re-distribute seed purchased wholesale) were excluded because they did not have a production location that could be spatially referenced. We contacted each company to ensure they met the above criteria. We identified 46 production companies that operated 50 production locations (Fig 3). Some production locations did not have a physical address (e.g., when the location was an agricultural field with no associated structures). Those locations were recorded as the center of the nearest township. Addresses or townships were georeferenced using Google Earth Pro (7.3.2), then exported to ArcGIS (10.6, ESRI, Redlands, CA). Because location information could include proprietary information about a given producer, we could not publicly release this data. We acknowledge that some grassland species’ seed appropriate for native plantings in our area are produced outside our focal region [26]. A nationwide assessment was beyond the scope of our study.

We created 500 hypothetical restoration sites (hereafter ‘restoration sites’) as points randomly chosen within the Level II Temperate Prairie Ecoregion within the upper Midwest US (hence the model’s sampling region; Fig 1). The points were stratified such that the number of points in each state is proportional to the area within each state classified as Temperate Prairie.

We used seven sources (S2 Table) to compile a list of exotic plant species identified as noxious weeds, federally or at the state-level, within at least one of the five states in our model’s sampling region. Using that list, we visually assessed each species’ county-level distribution based on three sources [10, 27, 28]. A species was removed from consideration if none of the sources showed it to be within our focal region, or if it was unlikely (<30% of the time; [29]) to be considered a ‘new’ invader (i.e., already within 25 miles (40.2 km) of a restoration site. We adopted a strict definition of ‘new’ (25 miles, 40.2 km) to represent similar definitions of local seed sourcing we found to be common among our practitioner colleagues. Next, we restricted the exotic plant species used to: 1) species capable of moving with native species’ seed as contaminants (i.e., reproduce by seed), 2) terrestrial, herbaceous species, as aquatic and woody exotic species are not typically weed species of concern in agricultural-like production fields, and 3) are problematic in open, grassland-like habitats (i.e., are not shade-loving, such as *Alliaria petiolata*, garlic mustard). Forty-seven exotic species met the above criteria. For those species, county-level occurrence data were collected (or converted to county-level from point data) from 31 sources, primarily databases, and primary and grey literature found using the species’ name and ‘distribution’ as search terms connected with ‘AND’ on Web of Science (© 2019 Clarivate) and Google (S3 Table). Data were collected directly in ArcGIS whenever possible, otherwise data were georeferenced using Google Earth Pro, then exported to ArcGIS. These data are publicly available through ScienceBase [30].

#### Analyses

We used logistic regression [31] to relate the cumulative probability of occurrence, either of a production location or exotic species, to distance from a restoration site. The line that relates the probability of occurrence of a seed production location to distance from a restoration site is a measure of availability. We took the complement of the probability of a weed being present as a metric of safety and solved for the distance where the availability and safety lines intersected. That distance is the ‘optimal maximum distance’, the maximum distance from a restoration site for sourcing native plant species’ seed to minimize the risk of introducing a new exotic species while retaining as much seed availability as possible. We ran a series of simulations to determine how the number of exotic species per restoration site affected variation about the slope and intercept of logistic regressions. Based on those simulations (S1 Appendix), we set a minimum sample size of 25 new exotic species per restoration site. Seventeen restoration sites did not meet this minimum and were removed. Note, these sites clustered in southeastern Nebraska (Fig 1), and the results from that region should be interpreted with caution. Using the remaining 483 restoration sites, we extracted the straight-line distance in kilometers from each restoration site to each production location, and from each restoration site to the edge of the nearest county containing an occurrence for each exotic species (see S2 Appendix for each species’ distribution map). Each restoration site was given a unique identifier allowing the two distances to be linked during analyses and the spatial reference maintained for presenting results.

Optimal maximum distances were calculated for each of the 483 restoration locations, mapped in ArcGIS, and solutions for all other possible locations were interpolated by using the IDW (inverse distance weighted) tool with default settings in ArcGIS.

#### Randomization tests

Two randomization tests were performed individually to assess the effects of each real dataset, production locations and exotic species’ distributions, on optimal maximum distances. For production locations, 50 random points were chosen within our focal region and the entire analysis was re-run using those points in place of the real production locations, while maintaining each restoration site’s real exotic species data. For exotic species distributions, we created a new dataset for each restoration site by randomly sampling the entire exotic species dataset without replacement, the same number of times as the sample size of each site. So, each restoration site had a new set of randomly selected species and distances, but a given species could occur more than once for an individual restoration site. The entire analysis was re-run using the revised exotic species dataset in place of the real exotic species data, while maintaining each restoration site’s real production location data. Optimal maximum distances obtained with random production locations or random exotic species were compared to actual data with Welch’s t-test. The goal of these analyses was to ensure that our results were not an artifact of measuring several distances within a defined area; results provided insight on the importance of production locations or exotic species’ distributions, but did not measure the relative contribution of each to the models compared to one another.

### Scenarios for alternative restoration practices

#### Sourcing via facilitated migration to address climate change predictions

To assess facilitated migration as a sourcing option in anticipation of warming due to climate change, we altered both the exotic species data and production data to reflect sourcing of seed south of a given restoration site. For each restoration site, we calculated the absolute geodesic angle from a given restoration site to each exotic species and to each production location (where 0˚ = North, +/-180˚ = South). All exotic species and production locations with absolute geodesic angles less than 90˚ were removed from the analysis. We removed restoration sites with fewer than 25 exotic species or 25 production locations from consideration, to maintain the same standards as in earlier renditions of the main model. With this exercise we were asking if the optimal maximum distance changes if seed sourcing follows a simplified facilitated migration strategy that assumes seeds will be sourced south of the restoration site. It provides one example of how practitioners might source for adaptation to climate change and the implications for importing exotic weeds.

#### Cost of control of exotic species

Another concern expressed by managers regarding inadvertent exotic species introduction is the cost associated with a federal mandate for its control under the Federal Noxious Weed Act. Within each set of exotic species for a given restoration site, all exotic species not listed as a federal noxious weed were removed from the dataset, and those that were on the list and known to invade row crop agriculture, pastures, rangeland, natural areas, or grasslands were retained for analysis. As with the original and climate-change models, we retained only those restoration sites with 25 or more exotic species for subsequent analysis. With this exercise, we are asking if the optimal maximum distance changes when we include only those species with a potential ‘cost’ associated with a federal mandate for control.

## Results

### Model

**Fig 4:**
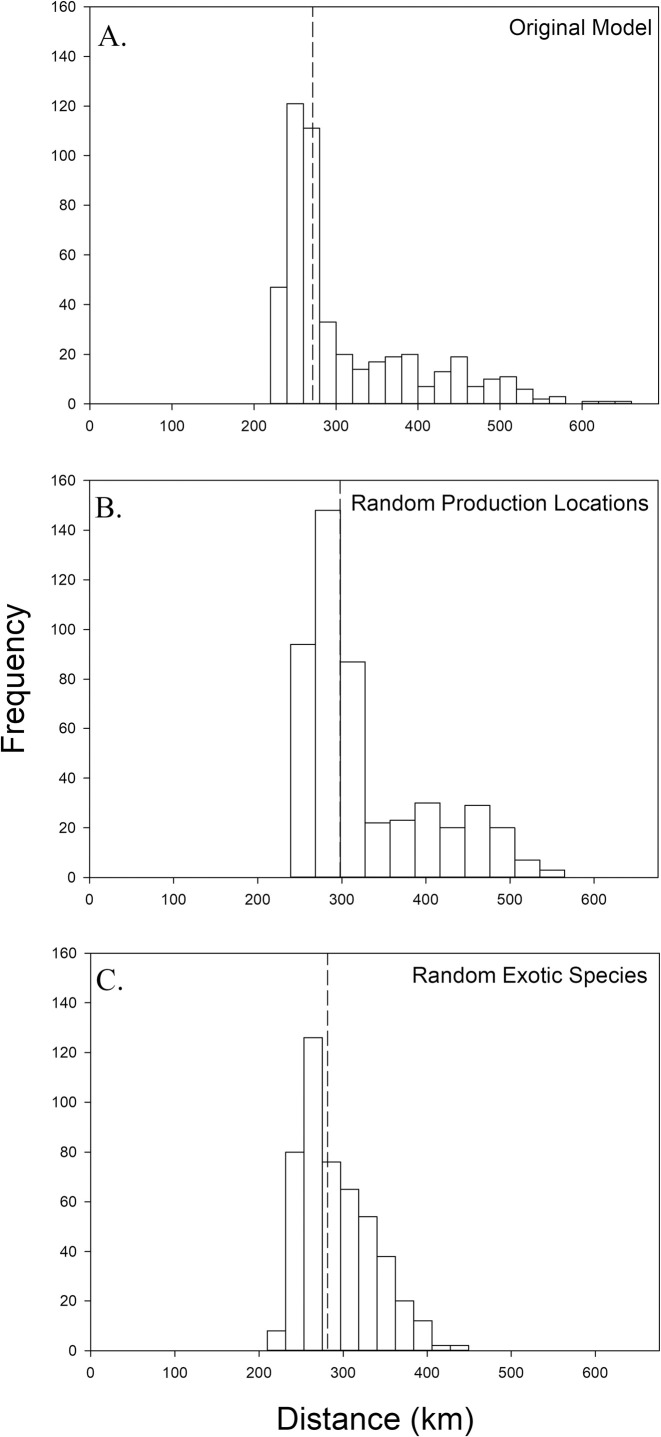
Histograms of modeled results estimating the optimal maximum distance from a given restoration site where sourcing native plant species’ seed minimizes the risk of introducing a new exotic species. Modeled results to show central tendency (medians as dotted line) and range. A) Result for the main analyses. B) Results from the randomization test for the production locations across the focal region. C) Results from the randomization test for the exotic species new to each site.

**Fig 5:**
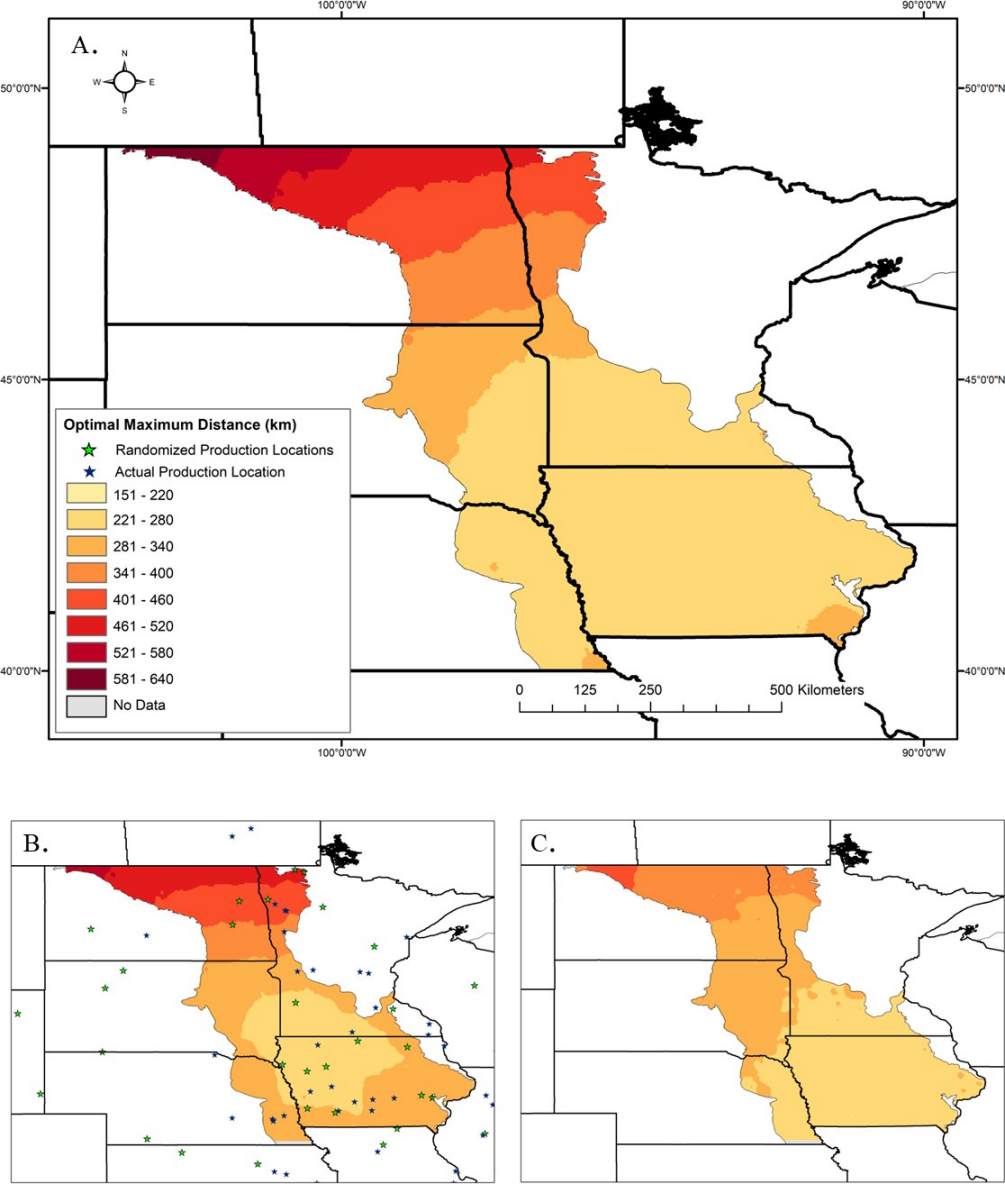
Maps of modeled results to show the spatial distribution of optimal maximum distance values across the sampling region. Those values were interpolated across the sampling region using the inverse distance weighted (IDW) tool in ArcGIS and binned by manual intervals to allow for comparison among maps. A) Interpolation of the main results. B) Results from the randomization tests for the production locations across the focal region. C) Results from the randomization test for the exotic species new to each site. US state and county data are publicly available from the US Census Bureau and are not subject to copyright (https://www.census.gov/geographies/mapping-files/time-series/geo/carto-boundary-file.2017.html). Canadian province data are publicly available through Statistics Canada and also are not subject to copyright (https://www150.statcan.gc.ca/n1/en/catalogue/92-160-X).

To determine the optimal maximum distance from a given restoration site where sourcing native plant species’ seed minimizes the risk of introducing a new exotic species, we used linear approximations to calculate attributes we defined as safety and availability; the optimal maximum distance is the intersection of the two logistic regressions. Our logistic regression analyses for safety and availability for all restoration sites in the model had R^2^ values between 0.723–0.972 (safety) and 0.880–0.986 (availability). The optimal maximum distance within our study area ranged from 220–653 km in a right-skewed distribution (Fig 4A) with a median value of 272 km (169 miles). The optimal maximum distance is lowest in the southern half of our focal region and increases somewhat radially from there (Fig 5A). Notably, production locations and exotic species are generally more concentrated in the southern half of our focal region (Fig 3).

To test if availability assessments and corresponding estimates of optimal maximum distance were sensitive to production locations, 50 random locations were selected within the focal region (Fig 3; note that we zoomed in on the sampling region to better compare effects of model scenarios). The most noticeable differences between the randomly chosen locations and the real production locations (Fig 3) are that the clump of actual production locations in Iowa and southern Minnesota is lost and that fewer fall within the model’s sampling region. In this randomization test, the range of the optimal maximum distances was slightly decreased (239–565 km) and the median was slightly increased (298 km) in comparison to the real result (Fig 4B compare to Fig 4A). The spatial structure of the results does not substantially change, but Welch’s t-test indicates that optimal maximum distance results are affected by the true coordinates of the production locations (t = -2.992, df = 941.35, p = 0.003). The area where the optimal maximum distance is lowest retracts (Fig 5B compare to Fig 5A). Because actual production locations are more concentrated in the southern portion of the focal region (Fig 3), when production locations are chosen at random, the distance to a production location would increase, resulting in greater optimal maximum distance values, especially for the southern restoration sites.

We assessed the contribution of exotic species’ ranges to the model by creating a new exotic species dataset for each restoration site and randomly sampling the entire dataset without replacement, the same number of times as the exotic species’ sample size of each site. The entire analysis was re-run using those datasets in place of the real exotic species data, while the actual production location data was retained. The frequency distribution of the optimal maximum distances is less right-skewed when the exotic species’ data are randomized compared to the real result (Fig 4C compare to Fig 4A). Again, the range is reduced (210–449 km) and there is a slight increase in the median (282 km) compared to the original model (272 km). However, here Welch’s t-test indicates the means of the two models are different (t = 4.4561, df = 704.03, p < 0.0001). With randomized exotic species, the area where the optimal maximum distance ranges from 221–280 km retracts slightly compared to the main model, while the areas with an optimal distance between 281–400 km greatly expand. This expansion is the likely cause of the increased median distance. The north-south gradient remains with exotic species randomization, but the optimal maximum distance is greatly reduced in the northwest portion of our sampling region, compared to the main model (Fig 5C compare to Fig 5A). Together, these results indicate that our model is sensitive to both the distances between restoration sites and exotic species’ range edges and the distances between restoration sites and seed production locations.

### Other restoration practitioner concerns

#### Sourcing via facilitated migration to address climate-change predictions

**Fig 6:**
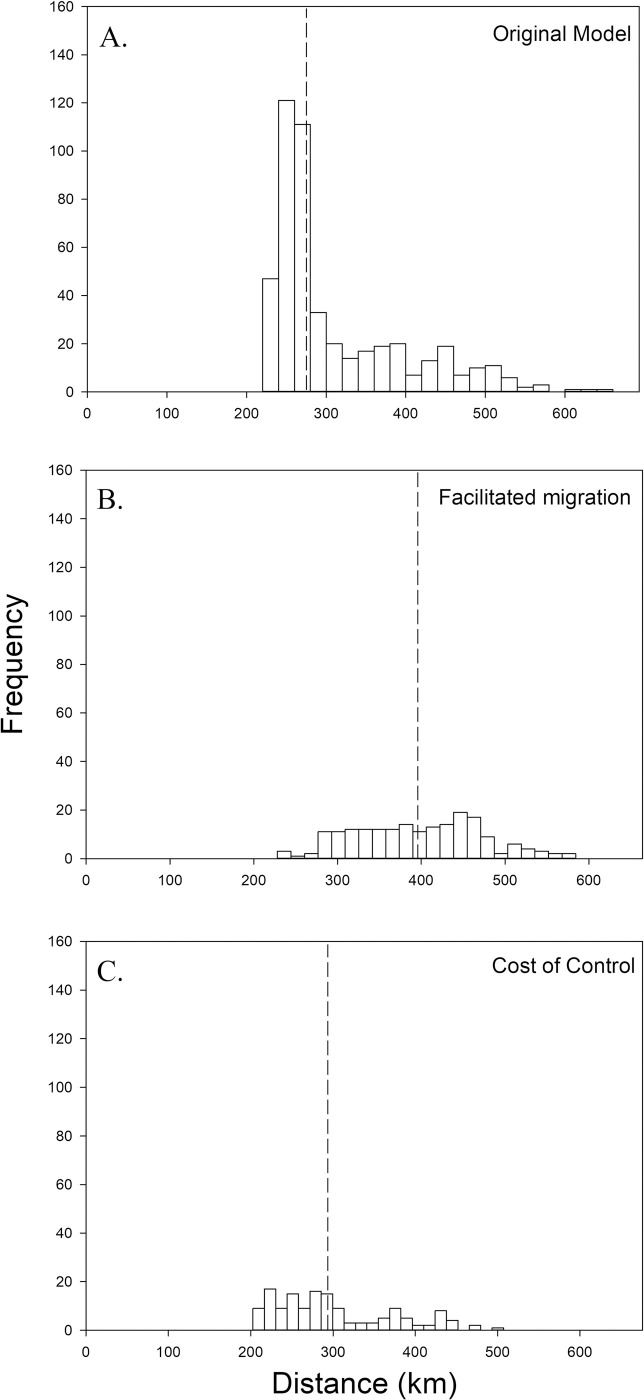
Frequency histograms of optimal maximum distances (medians as dotted line) for A) the baseline model that relies on locations for 50 seed production areas and 47 weed species; B) a model for facilitated migration, restricting seed production areas and weed species to those south of a restoration site; and C) a model that allows all current seed production areas but focuses on federally listed noxious weed species.

**Fig 7:**
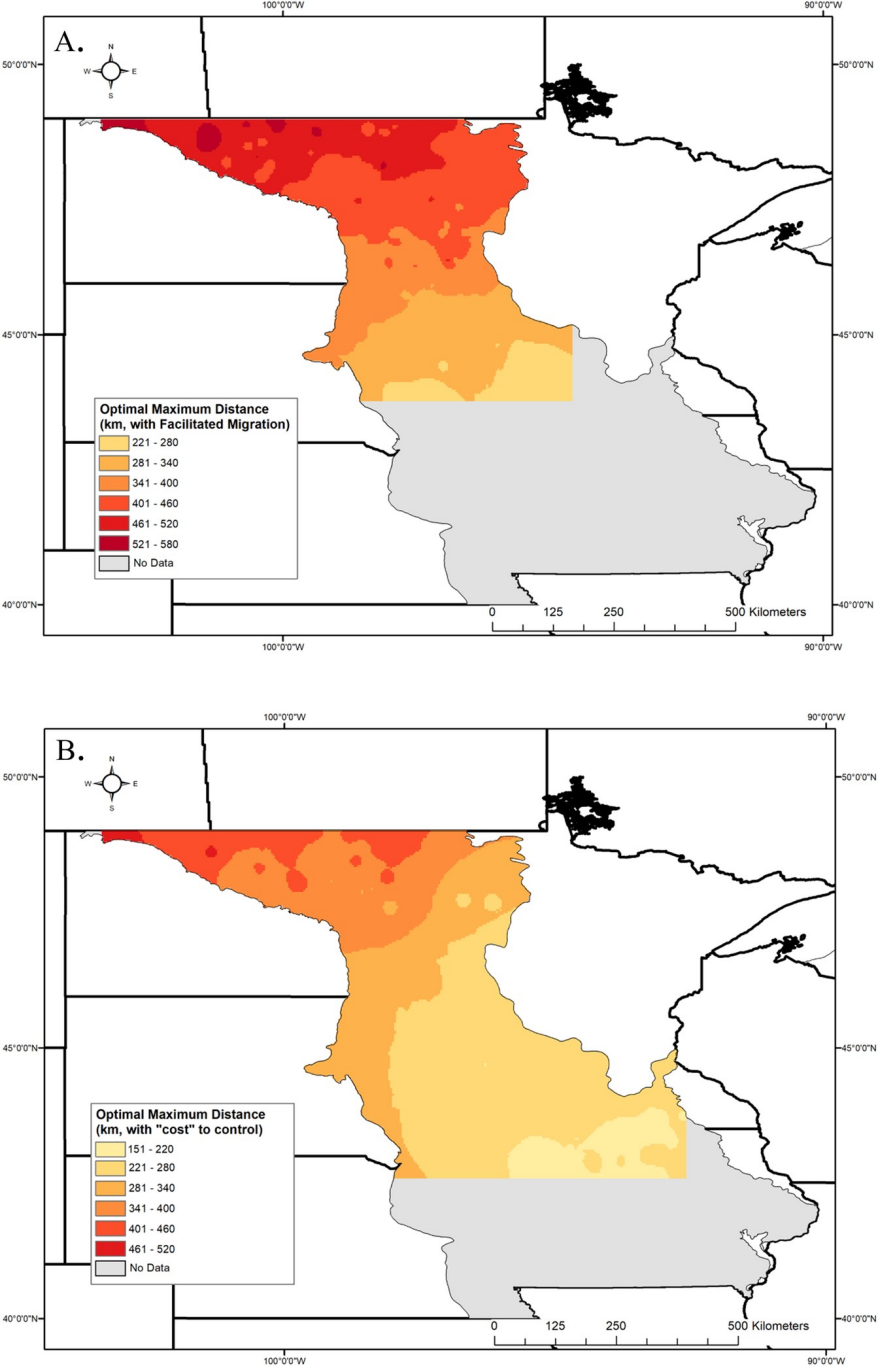
Maps of modeled results to show the spatial distribution of optimal maximum distance values across the sampling region in the facilitated migration and cost of control weighting exercises. Those values were interpolated across the sampling region by using the inverse distance weighted (IDW) tool in ArcGIS. A) Results for facilitated migration exercise, where all exotic species and production locations north of a given restoration site were removed from analysis. B) Results for the cost of control exercise, retaining only those exotic species with a federal mandate to control and that are known to invade one of three habitats: row agriculture crops, rangeland/pasture, or natural habitat/grassland. US state and county data are publicly available from the US Census Bureau and are not subject to copyright (https://www.census.gov/geographies/mapping-files/time-series/geo/carto-boundary-file.2017.html). Canadian province data are publicly available through Statistics Canada and also are not subject to copyright (https://www150.statcan.gc.ca/n1/en/catalogue/92-160-X).

Because practitioners may choose to use a facilitated migration seed sourcing strategy in anticipation of warming due to climate change, we modified both exotic species and production data such that sourcing of seed would only take place south of a given restoration site. In requiring seed sources to be south of a given restoration site and focusing on exotic weeds in those areas the optimal maximum distance range contracted from the original model (with facilitated migration: 228–584 km; original model: 220–653 km), and the median increased substantially from 272 km to 398 km (Fig 6B). The increase in the median is likely a result of the removal of many of the restoration plots from the southern region, due to inadequate sample sizes, where values of the maximum optimum distance were on the lowest end of the range from the original model (Fig 7A; e.g., below 280 km). Welch’s t- test indicates that optimal maximum distance results of the climate change model do not differ from the main model when comparing sites present in both models (t = -1.510, df = 371.88, p = 0.132).

#### Cost of control of exotic species

Removing exotic species that are not federally noxious from the model resulted in a slight increase in the median of the optimal maximum distance from 272 km to 283 km (Fig 6C). However, as with the facilitated weighting exercise, we lost a substantial number of restoration sites from the model in order to retain a minimum of 25 exotic species per site; 146 sites remained after exclusion. Similarly, there also was a contraction of the range in optimal maximum distance values (202–507 km) when compared to the original model. The gradient from low-high optimal maximum distance values persists, when moving from the southeast to the northwest corner of the sampling region, (Fig 7B)

## Discussion

### Seed sourcing guidelines

Seed source recommendations among agencies can vary widely, from “local” recommendations of <42 km (25 miles) to historical recommendations of up to 482 km (300 miles) away [32]. Most, however, do not give a definitive distance threshold to follow [but see 15, 30]. Instead, recommendations often include adherence to local ecotypes and avoidance of agronomic and horticultural cultivars [14, 33, 34], to preserve long-term fitness of extant populations that are (presumed to be) best adapted to the site [15]. While use of locally sourced seed in close proximity to a restoration is seen as ideal, there are often limitations on species availability in quantities needed for large-scale restorations. Using cultivated seed, often sourced from greater distances, increases availability but comes with increased risk of introducing exotic, invasive species’ seed into grassland restorations. Results from our analyses offer guidance on minimizing risk of exotic species’ introduction, while maximizing native seed availability.

Our model results suggest that, across our sampling region, an optimal maximum seed sourcing distance of 272 km, or approximately 170 miles, could reduce the risk of introducing a new exotic plant species as a contaminant of native species’ seed, while retaining as much seed availability as possible, measured as the number of acceptable production locations. That median value well represents most of the optimal maximum distance values calculated by our model, despite a long tail of higher values (Fig 4A). Those higher values were clustered spatially at the northern edge of our sampling region along the Canadian border (Fig 5A). While it is possible that there are fewer exotic species invading our sampling region from the north, this pattern is also likely in part due to occurrence data for exotic plant species being much less available from Canada (e.g., *Rhaponticum repens* in Appendix B). The lowest optimal maximum distance values are located in the southern half of our sampling region (Fig 5A). The randomization tests support that this area experiences both high pressure from nearby new exotic species, as can be seen when the spatial structure of the production locations is removed (Fig 5B), and high seed availability from nearby commercial production locations, as can be seen when the exotic species data are generated randomly (Fig 5C). Therefore, that is the region where it is most critical, but also most feasible, to source seed within our model’s suggested median optimal maximum distance.

When weighting our model for facilitated migration, the median optimal maximum distance increased from 272 km to 398 km (169–247 miles), when compared to the original model. However, in this exercise many of our random restoration sites were removed from consideration after either exotic species or production areas north of a given restoration site were removed from the analysis. The majority of restoration sites removed due to insufficient (<25) exotic species after weighting were from the southern half of our sampling region; this makes sense given how the data for the study were structured. Our emphasis was the Level II Temperate Prairie Ecoregion within the upper Midwest US. Exotic plant species certainly occur farther south of this region but were not included in this study due to logistical constraints. Further, we did not include seed production sites that are farther south than our focal region. Thus, our data quality standards dictated, appropriately, that we not forecast an optimal maximum distance for facilitated migration events in these southerly locations. Maintaining a strict minimum sample size allowed for better fit of linear models and greater precision in determining a specific intersection point between safety and availability. Despite the large increase in the median optimal maximum distance from this exercise (398 km, or 247 miles), the area for which we had enough data to interpolate remains relatively unchanged from the original model (compare Figs 7A–5A) and analyses suggest no significant difference from the main model. Because the southeast portion of our study region lacks sufficient data to interpolate optimal maximum distances, it may be best to take a conservative approach to sourcing from the minimum end of the optimal maximum distances from this weighting exercise (228 km, or 141 miles). The ability to do so will depend on sufficient availability of seed sources south of a given restoration site.

In addition to facilitated migration, restoration practitioners also expressed concern about federally listed noxious weeds, for which control is required, as compared to non-native invasive weeds in general. We addressed this by retaining only those exotic species with a federal mandate to control and known to invade row crop agriculture, pasture or rangeland, or grasslands, in our model; this approach to weighting substantially reduced the number of non-native, invasive species under consideration. The range of optimal maximum distances contracted compared to the original model (202–507 km) and only 146 random restoration sites remained for interpolation. Like the climate-change weighting exercise, restoration sites from the southern end of the sampling area were removed from consideration due to low sample sizes after removal of those exotic species with no associated mandated “cost.” However, despite the minimal change from the original model’s median optimal maximum distance (283 km or 175 miles), there is a noticeable visual shift in the optimal maximum distance categories, particularly on the lower end of the range in western Minnesota and northern Iowa (Compare Figs 5A–7B). Given this overall expansion of the lower end of the range of optimal maximum distances, one might again consider a conservative approach to sourcing seed from the lowest end of the categories provided (181–220 km, or 112–137 miles) in those areas for which no data for interpolation is available.

### Caveats

It is important to note that the above findings apply to the linear distance between a restoration site (or any native planting site) and the site where the seed used in that planting was grown, in the Level II Temperate Prairie Ecoregion within the upper Midwest. We believe this method provides a useful conceptual starting point for other regions, but formal testing of the approach was beyond the scope of the study. Application of the method requires knowledge of where plants for restorations are being produced, where exotic species that could be moved with those plants might be found, and where future restoration activities might occur. We cannot comment on the applicability of the method to other systems (e.g., forests or wetlands) as additional caveats beyond our experience may apply. To date, seed sourcing guidelines for native plant species are largely based on the linear, or sometimes ecological, distance between a restoration site and the genetic origin of the seed used in the planting [7, 15]. As such, many seed labeling and certification regulations for native seed require the genetic origin to be reported [35], not the location where the seed lot was grown. Thus, practitioners attempting to incorporate the findings provided here will likely have to request this additional information during the seed bidding process. However, labeling the location where seed was grown or wild-harvested has been required by law in the United States for agricultural or silvicultural seed for decades [8]. Thus, we anticipate this information will be available from seed suppliers upon request.

Testing commercial seed lots for the presence of noxious weed seeds is also required by law [8, 36]. This testing helps to ensure good field hygiene and seed cleaning procedures by growers and is a critical layer of protection for consumers. However, only a limited number of exotic species’ seed are regulated via testing, and the list of species’ seed that are prohibited or restricted varies by state [36]. Thus, while this regulation certainly helps prevent the spread of well recognized or highly noxious species, it does not provide comprehensive protection against seed-lot contamination by the many potentially problematic, exotic invaders encroaching on any given landscape.

Numerous idiosyncratic factors affect the true probability that a weed contaminant will be present in a seed lot. Such factors include the abundance of different weed seeds at the time of harvest, the efficiency with which weeds are removed by various post-harvest cleaning techniques, and the potential for contaminated seed lots to be detected during seed testing and regulatory inspections. Each of these factors is influenced by numerous other variables that may change over time (e.g., weed densities and weather conditions in production areas, efficiency of weed management, timing of harvest, size and shape of weed seeds, etc.). We consider efforts to account for all of these variables to be untenable. Our abstraction of the problem assumes a constant, non-zero probability of introduction for each exotic weed species that could occur in a production area. Thus, as additional exotic weed species are encountered, the probability that at least one of these species is present in a shipment of seed increases. Our analysis provides an empirical estimate of the rate at which new weed species might be encountered as distance from a restoration site increases and a baseline against which future analyses might be compared.

## Conclusion

Here we presented results from a decision framework in which we sought to quantitatively find the balance between two primary concerns of grassland restoration practitioners: 1. Availability of native prairie seed and 2. Risk of unintentional introduction of novel, invasive seed via contamination. Our analyses indicated that seed obtained from no more than 272 km from a prairie restoration site would balance these objectives. If a strategy of facilitated migration is pursued to adapt to anticipated future climate change, this distance would increase to 398 km to maintain current levels of seed availability. Our results are consistent with several current recommendations/requirements for maximum seed-sourcing distances in the sampling region but are more transparent in their derivation. We hope these results provide practical guidance to public entities that support prairie restorations and valuable insights to restoration practitioners about the potential hazards of importing seeds from long distances.

## Supporting information

S1 Fig Maximum optimal distance for sourcing seed from a hypothetical restoration site. A logistic regression was fit to seed availability and the complement of weed risk. The ‘optimal maximum distance’ is the distance at which these two regressions intersect and is represented by a circle with an “X” strike-through on the x-axis.
(TIF)

S1 Appendix Determining the number of ‘new’ exotic species needed per restoration site. (DOCX)

S2 Appendix Maps of each species’ county-level distribution used in this study. Bold black lines are borders of the US states or Canadian provinces of our study’s focal region. Thin black lines are the borders of the counties (USA) or census tracts (Canada). The crosshatched area is the Level II Temperate Prairie Ecoregion within the upper Midwest, which was used to delineate our model’s sampling region. Darkened counties or census tracts are known occurrences of the species in each map. A complete list of references used to gather distribution data is provided in S3 Table.
(DOCX)

S1 Table National and regional websites, databases, reports and publications documenting commercial sources for native plant species’ propagation materials. (DOCX)

S2 Table Sources for state and federal noxious weed lists. (DOCX)

S3 Table Total list of species used in the analyses for this study; sources used to obtain exotic species occurrence data is listed in endnotes. (DOCX)
