# Supplementary figures and images for: Balancing the need for seed against invasive species risks in prairie habitat restorations
Source: PLoS One. 2021 Apr 7;16(4):e0248583. doi: 10.1371/journal.pone.0248583 (PMC8026064; doi:10.1371/journal.pone.0248583)

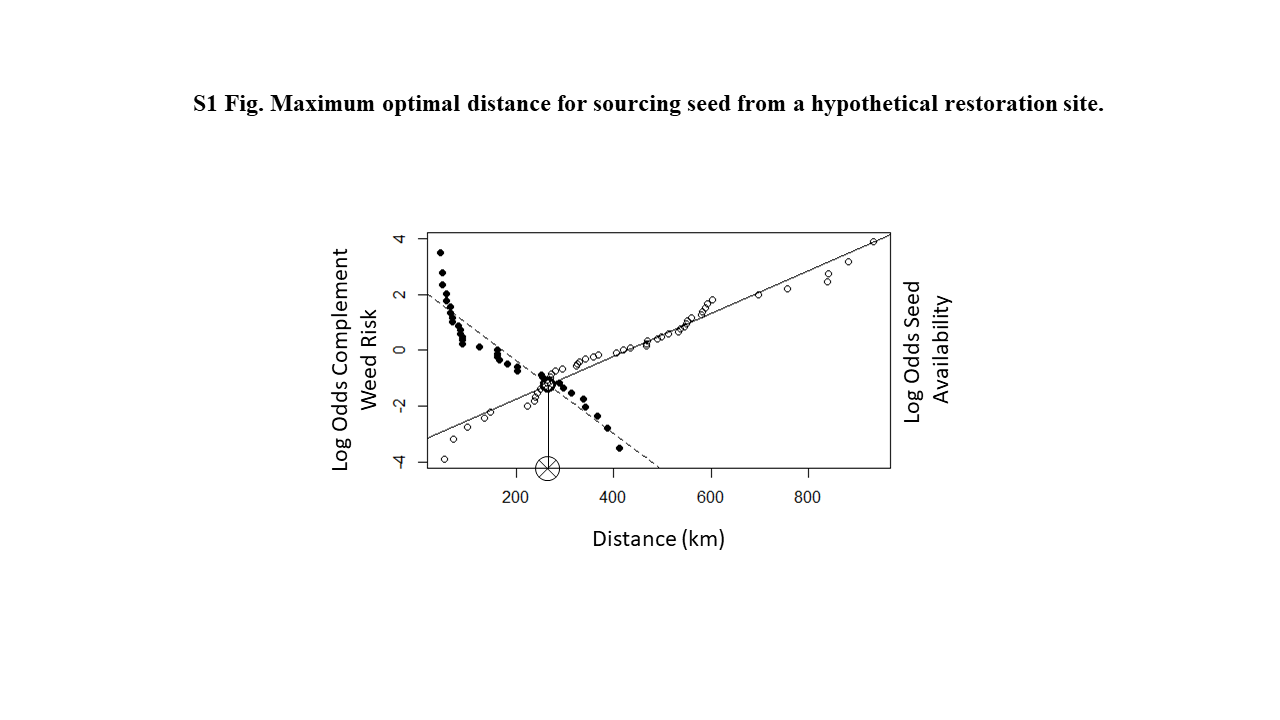

Supplement: S1 Fig — A logistic regression was fit to seed availability and the complement of weed risk. The ‘optimal maximum distance’ is the distance at which these two regressions intersect and is represented by a circle with an “X” strike-through on the x-axis. (TIF) [file pone.0248583.s001.tif]

S2 Appendix: Maps of each species’ county-level distribution used in this study.


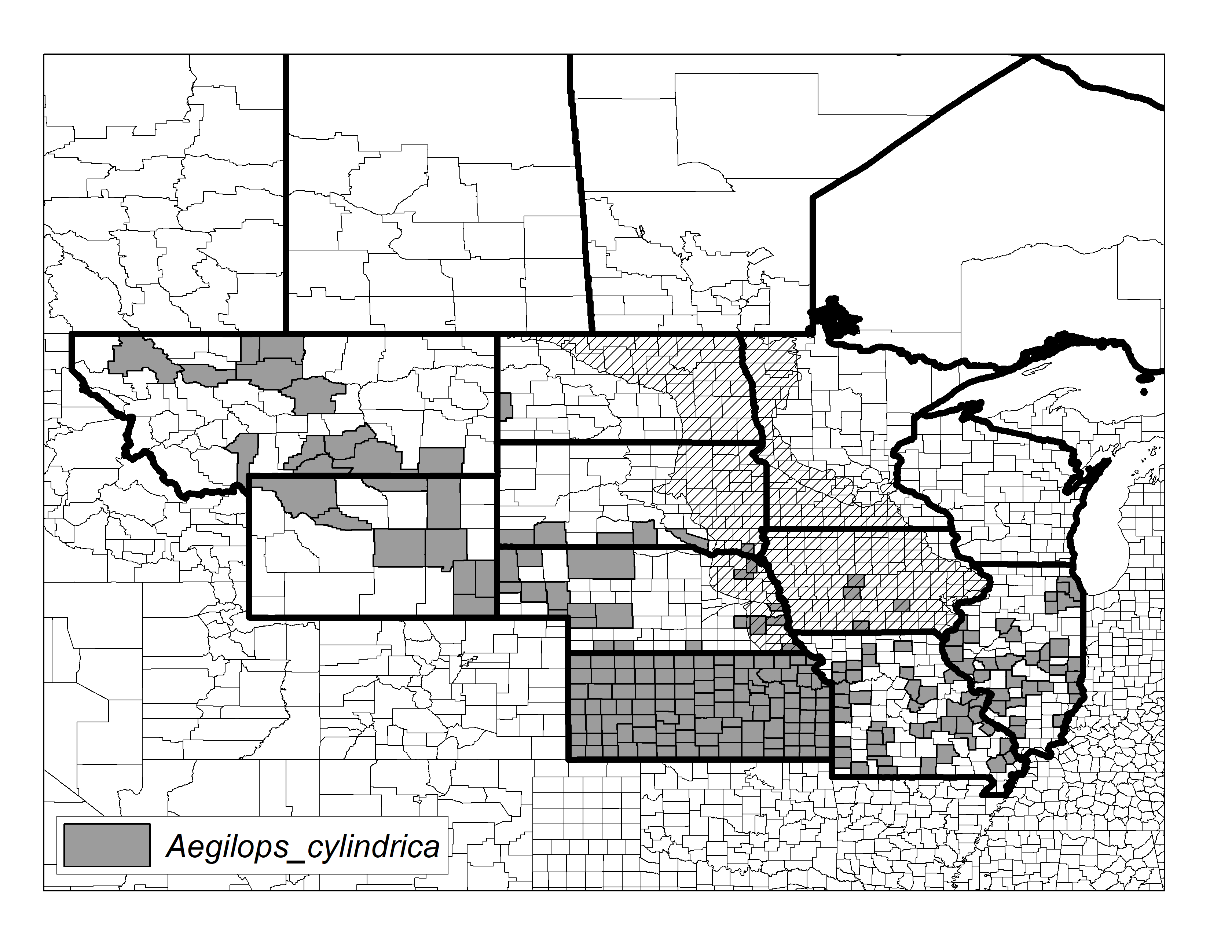


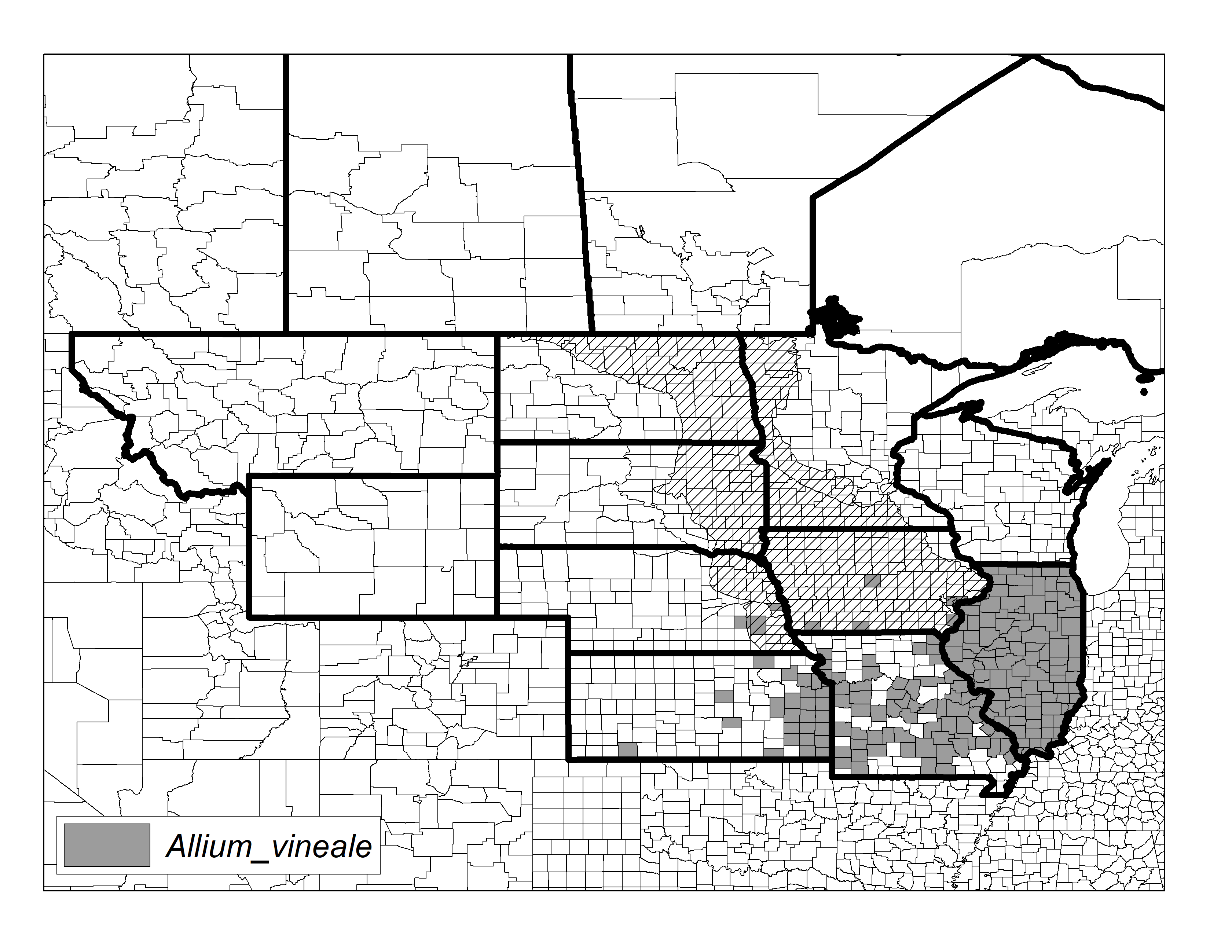

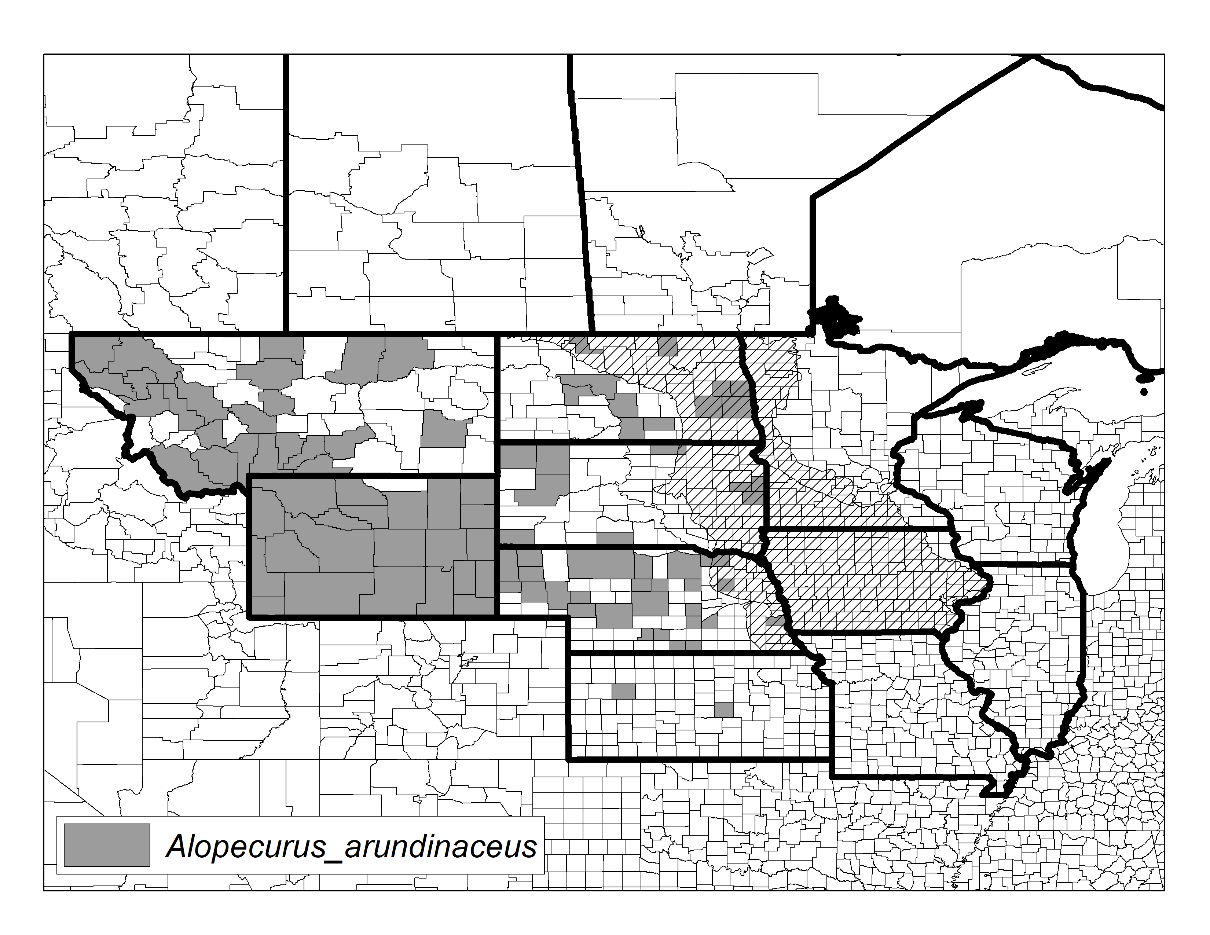


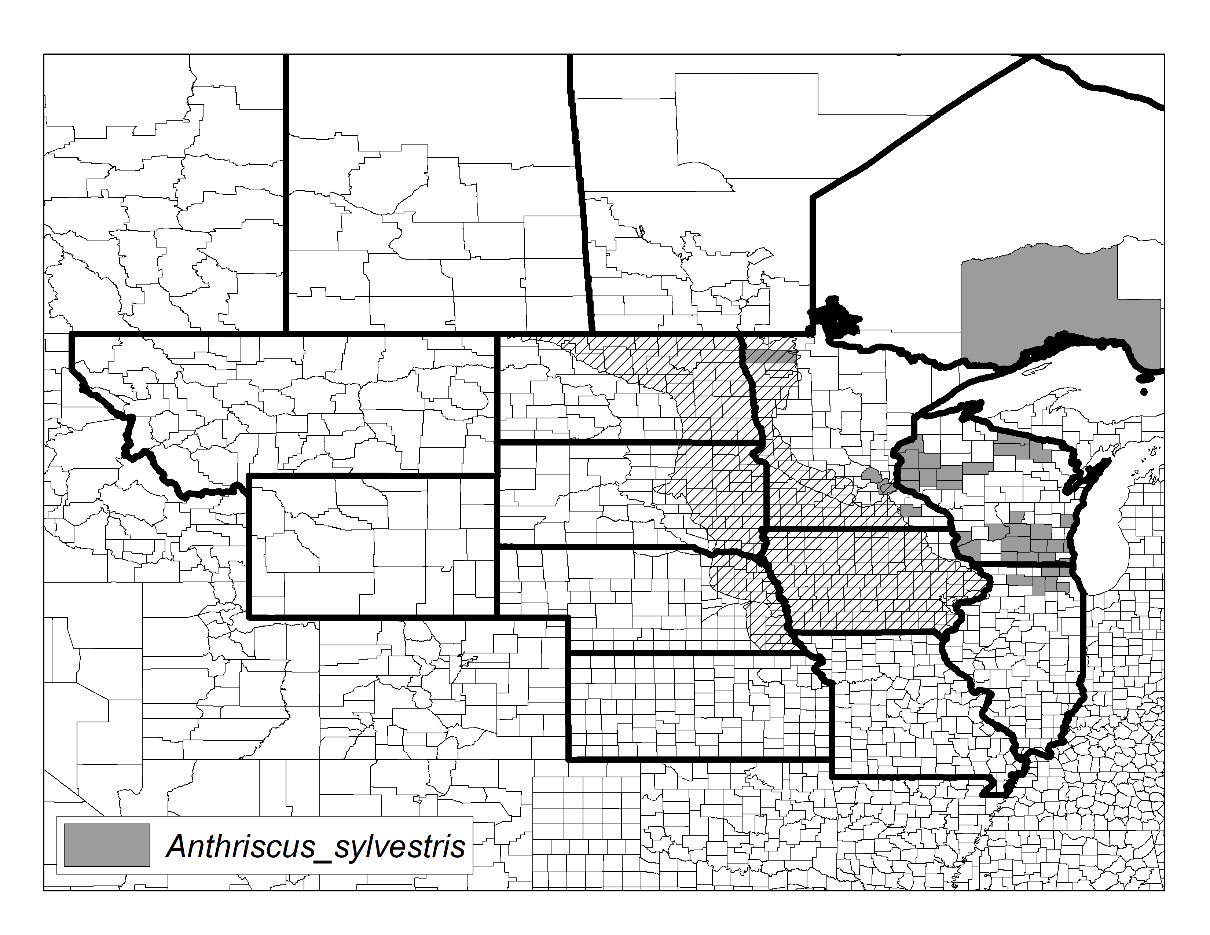

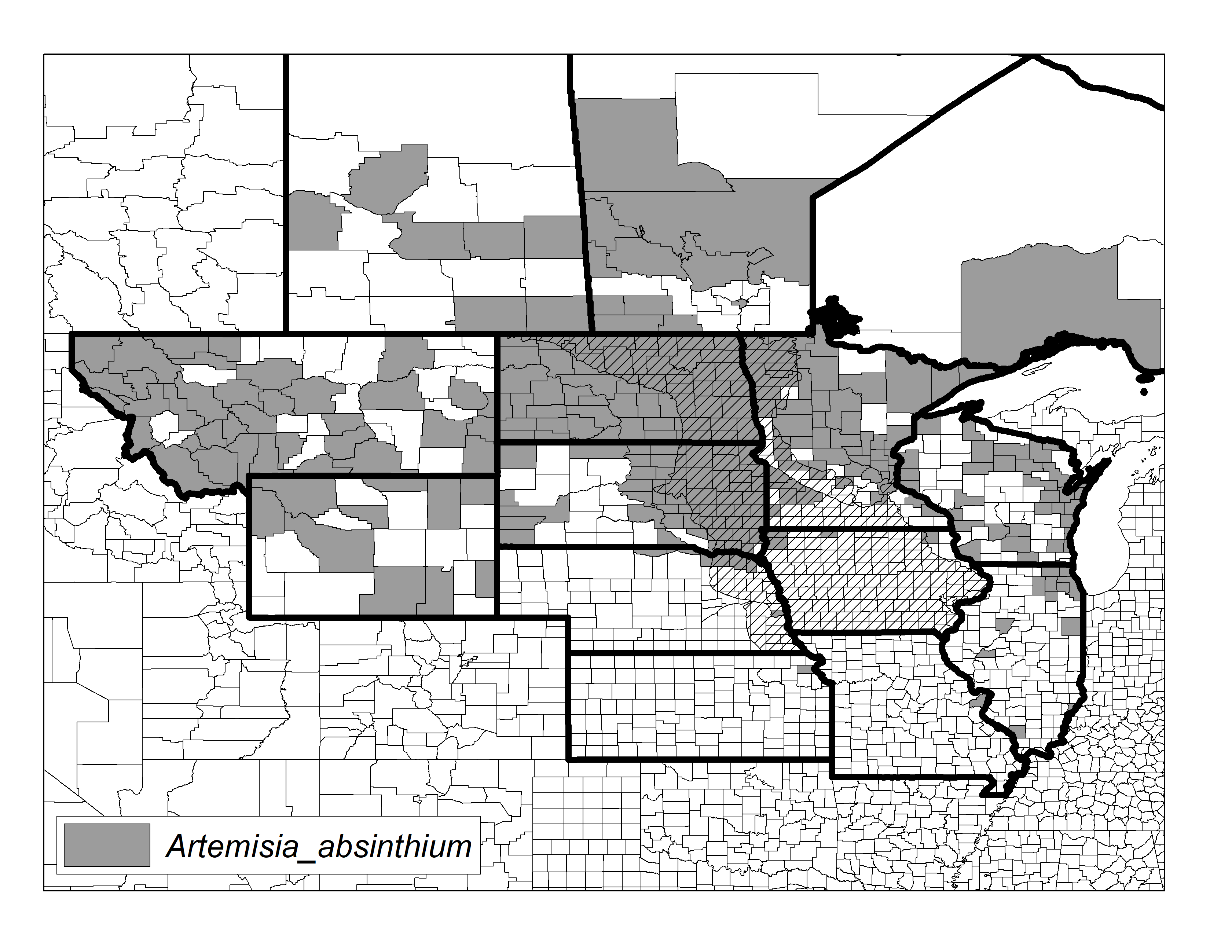


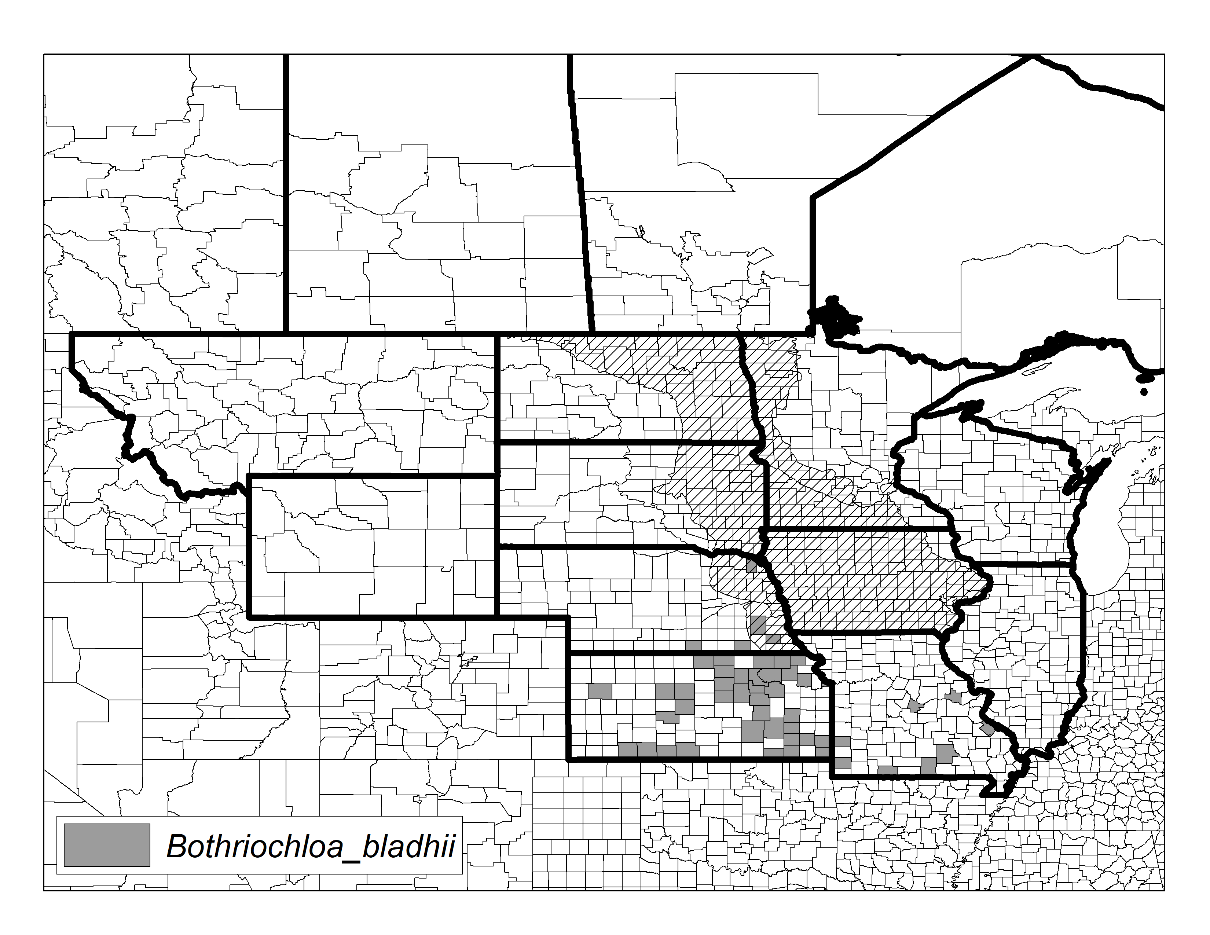

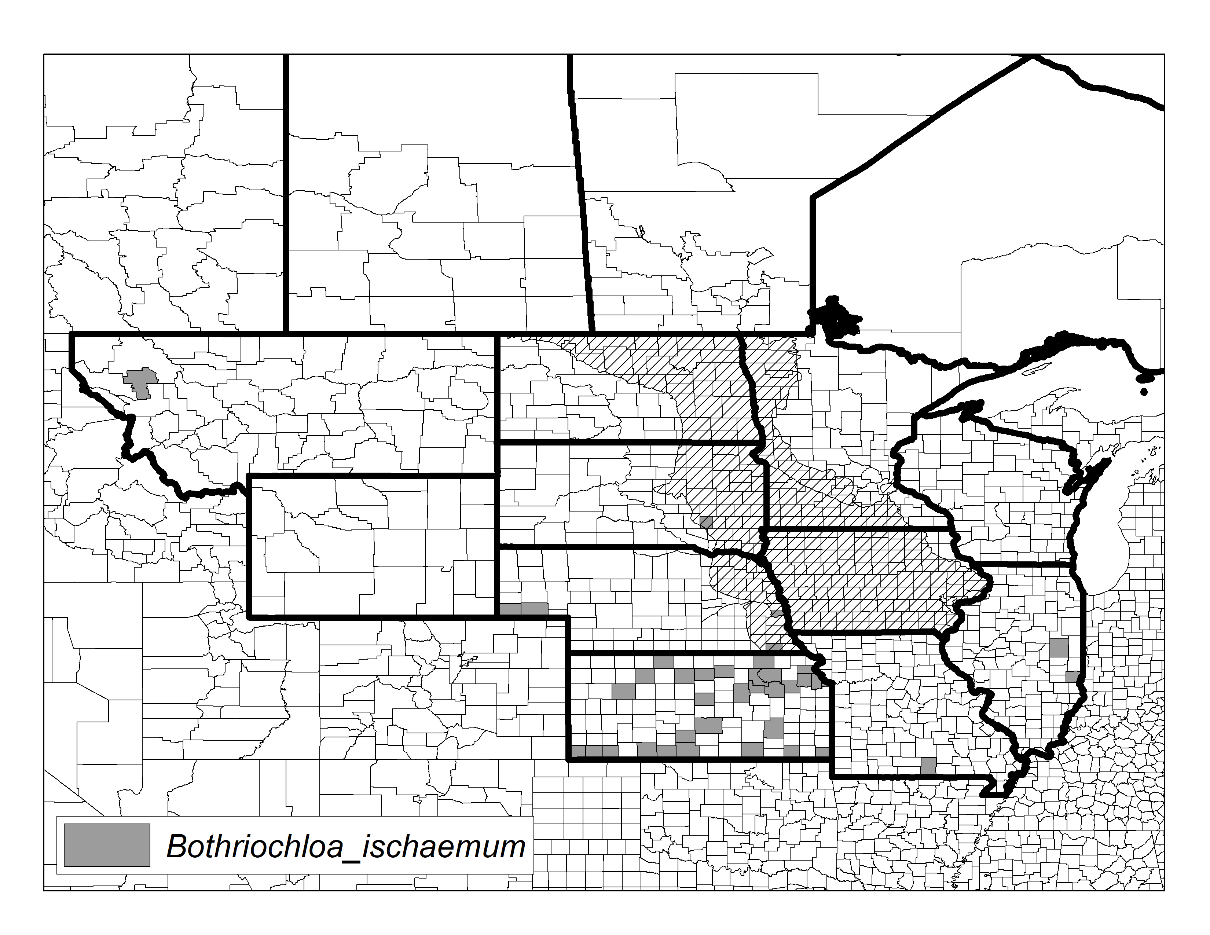


.
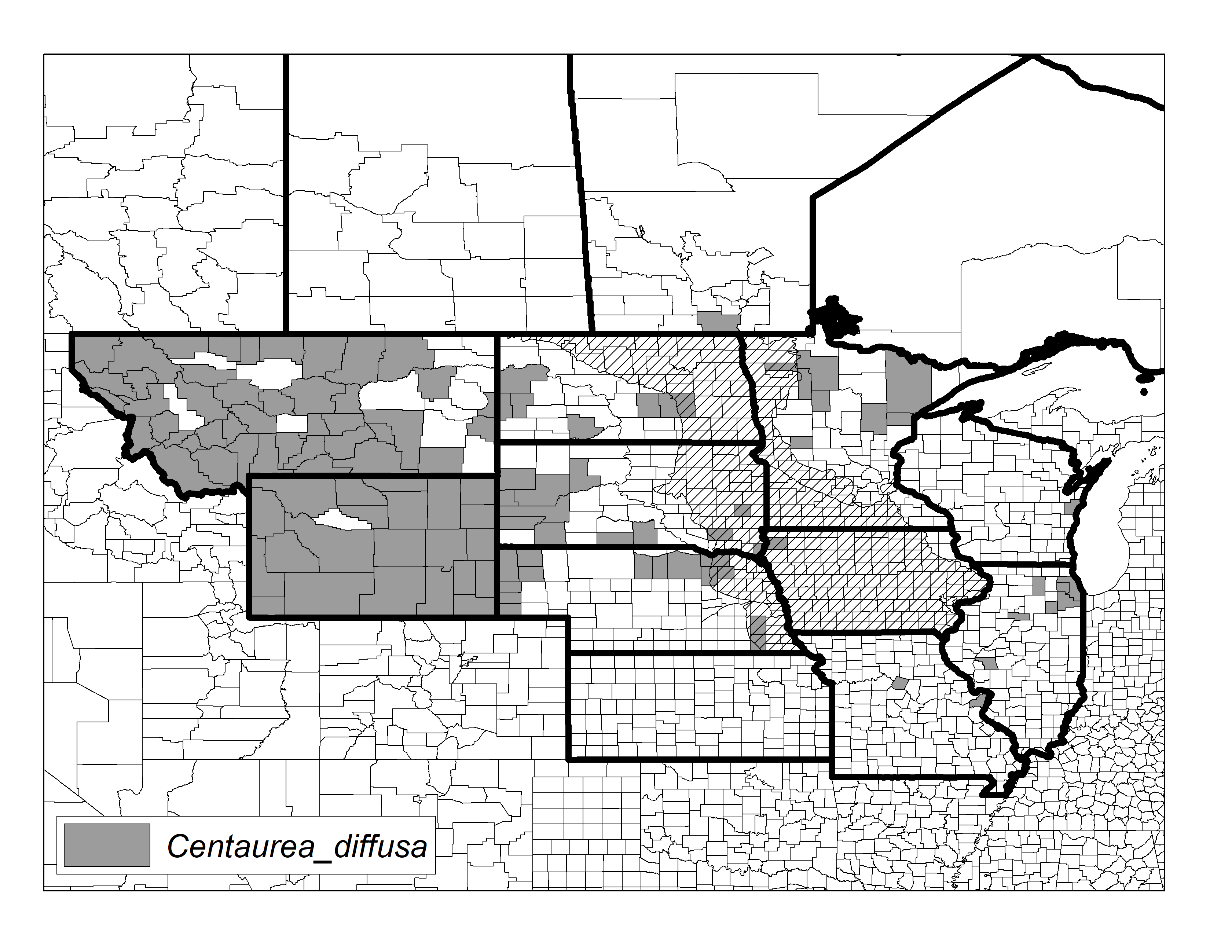

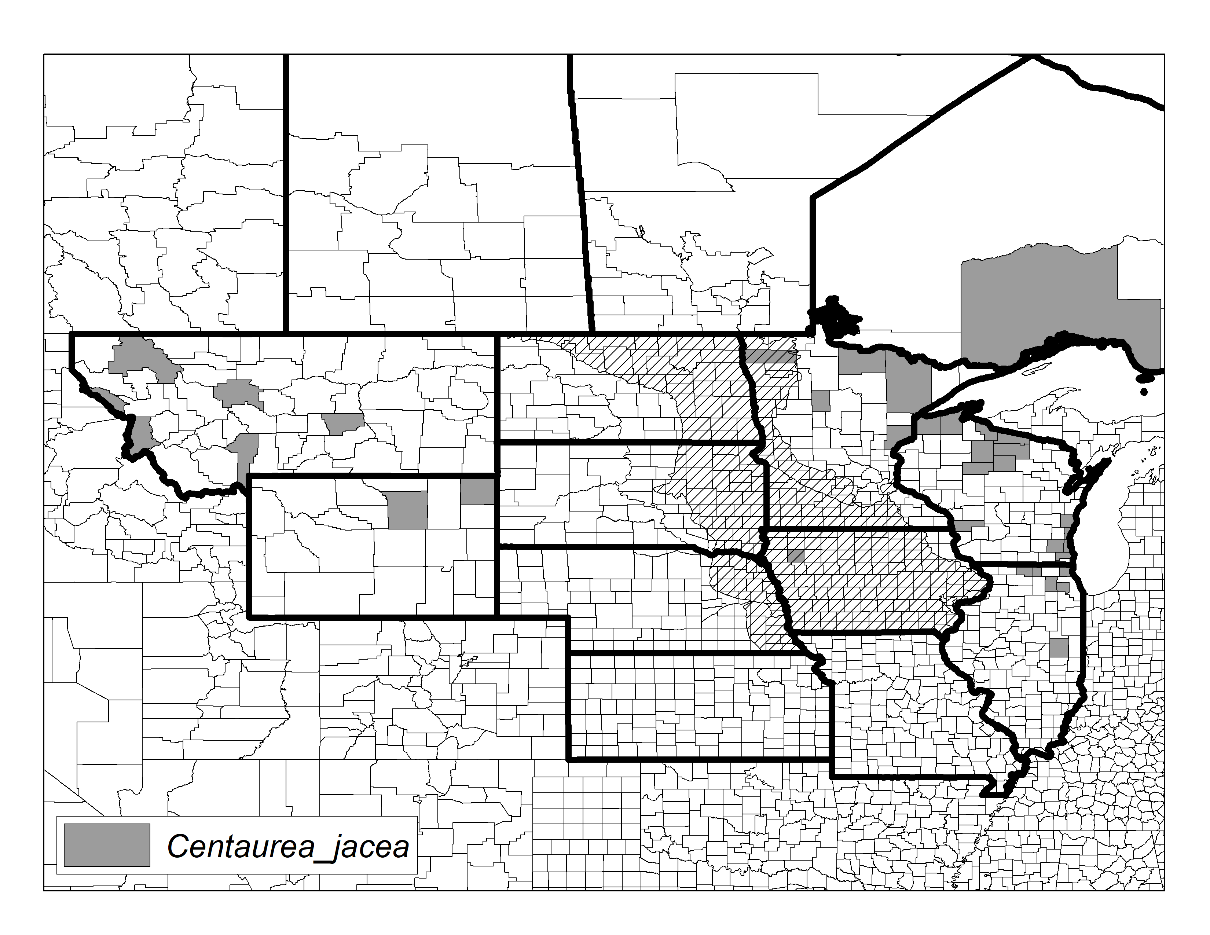


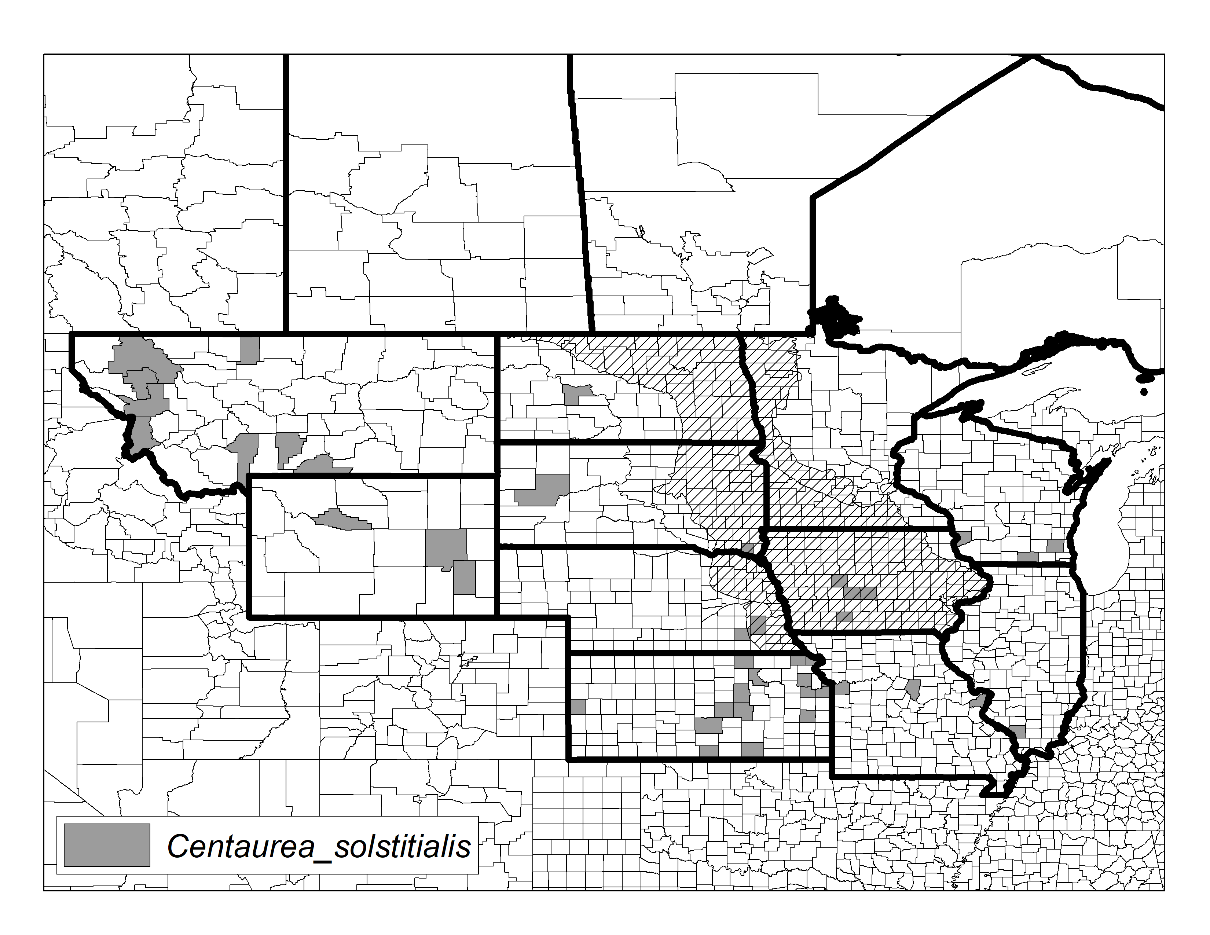

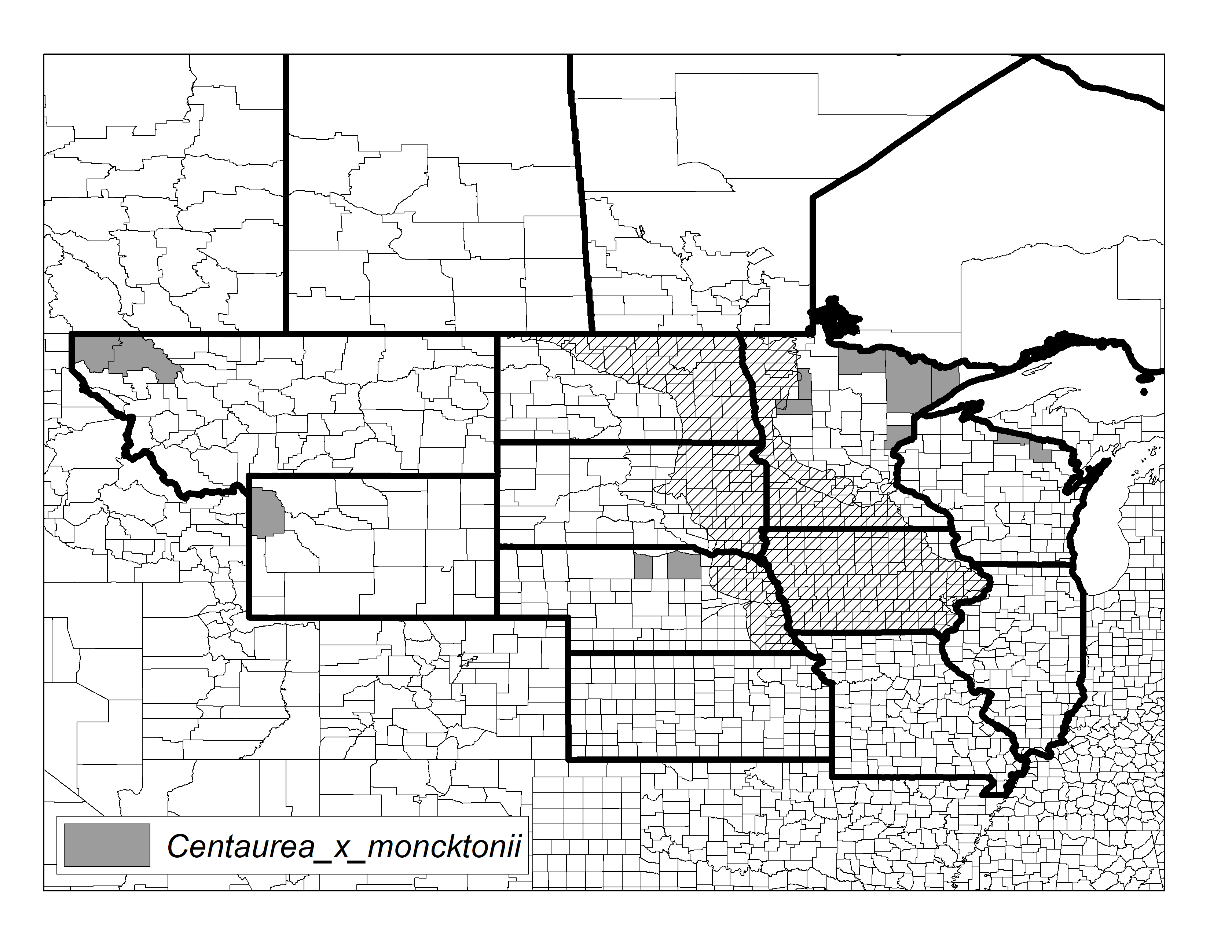


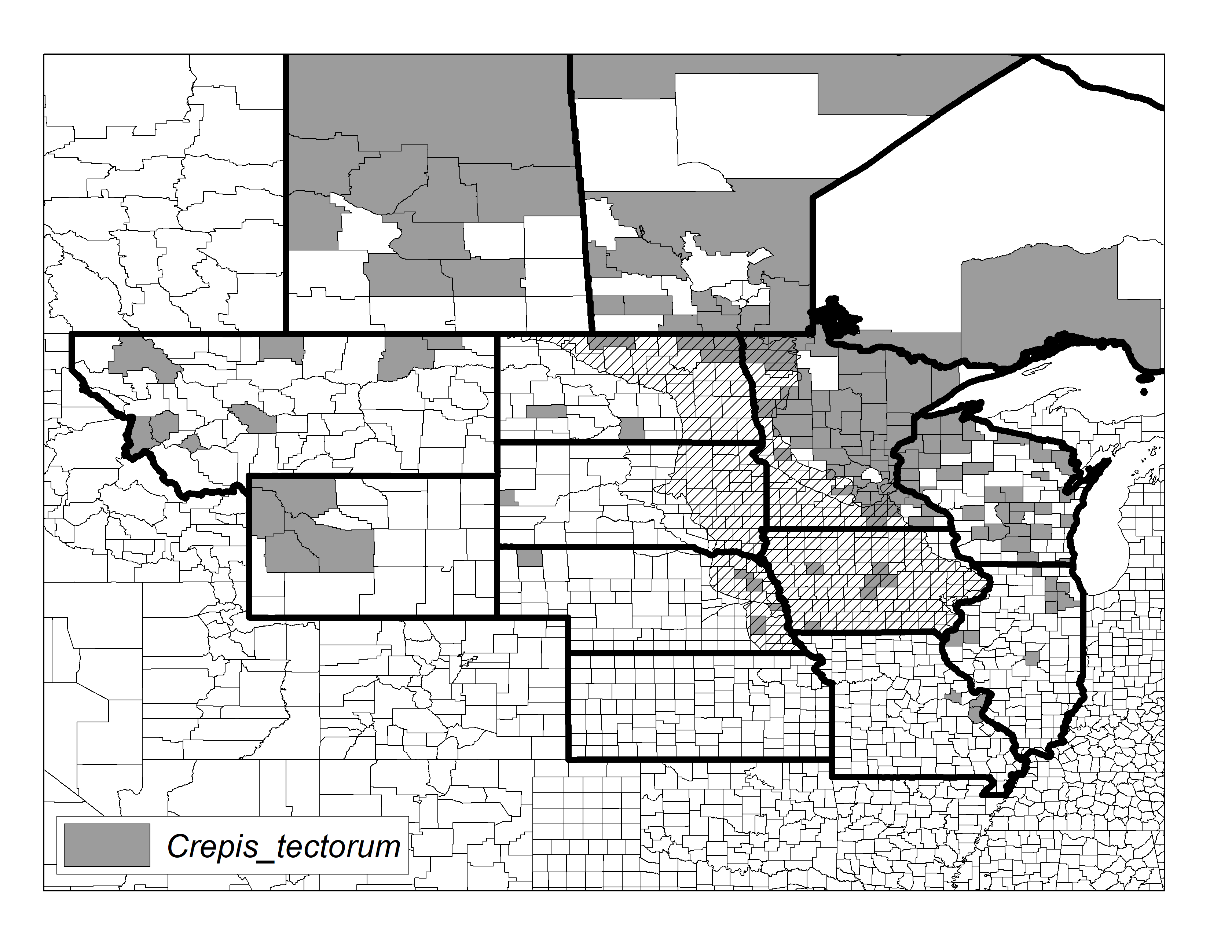

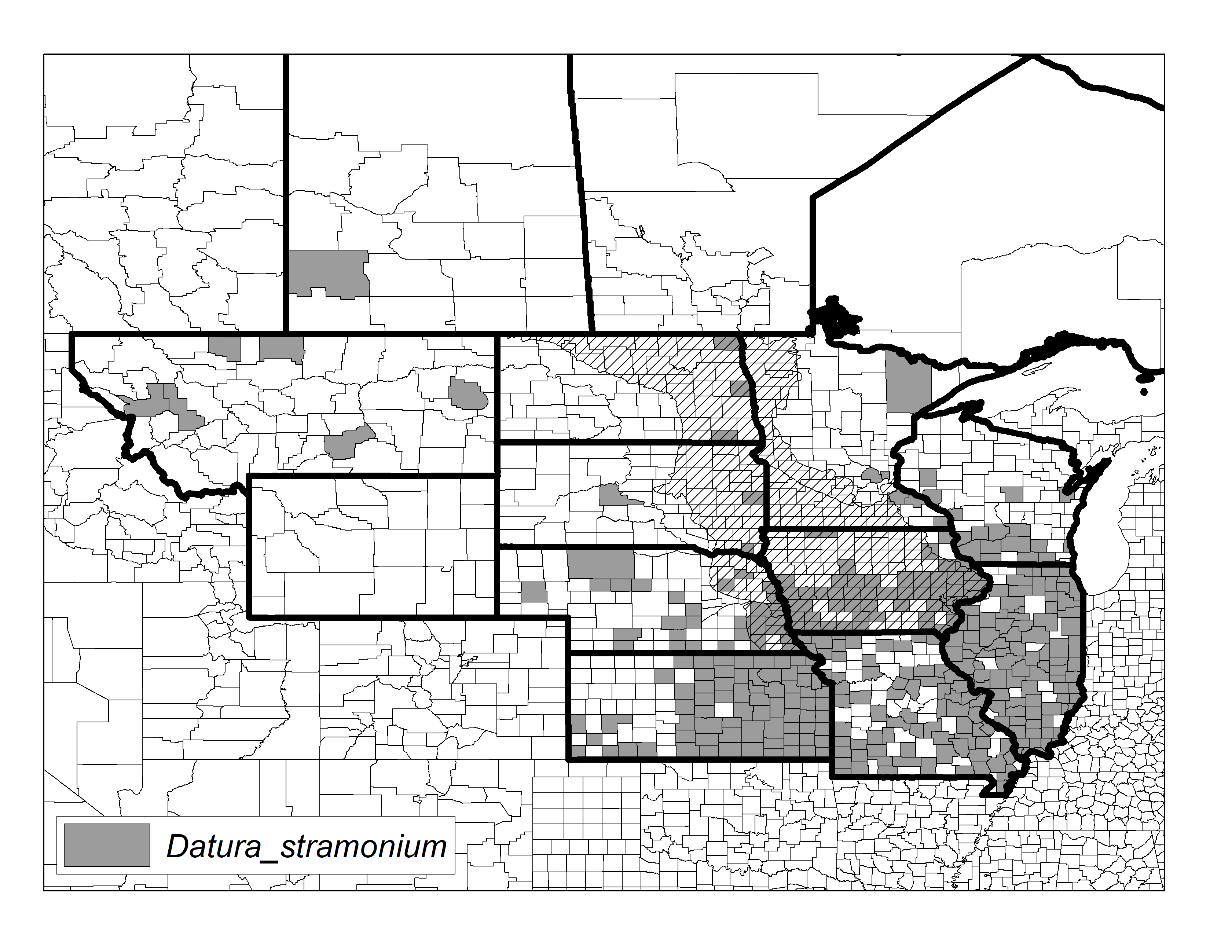


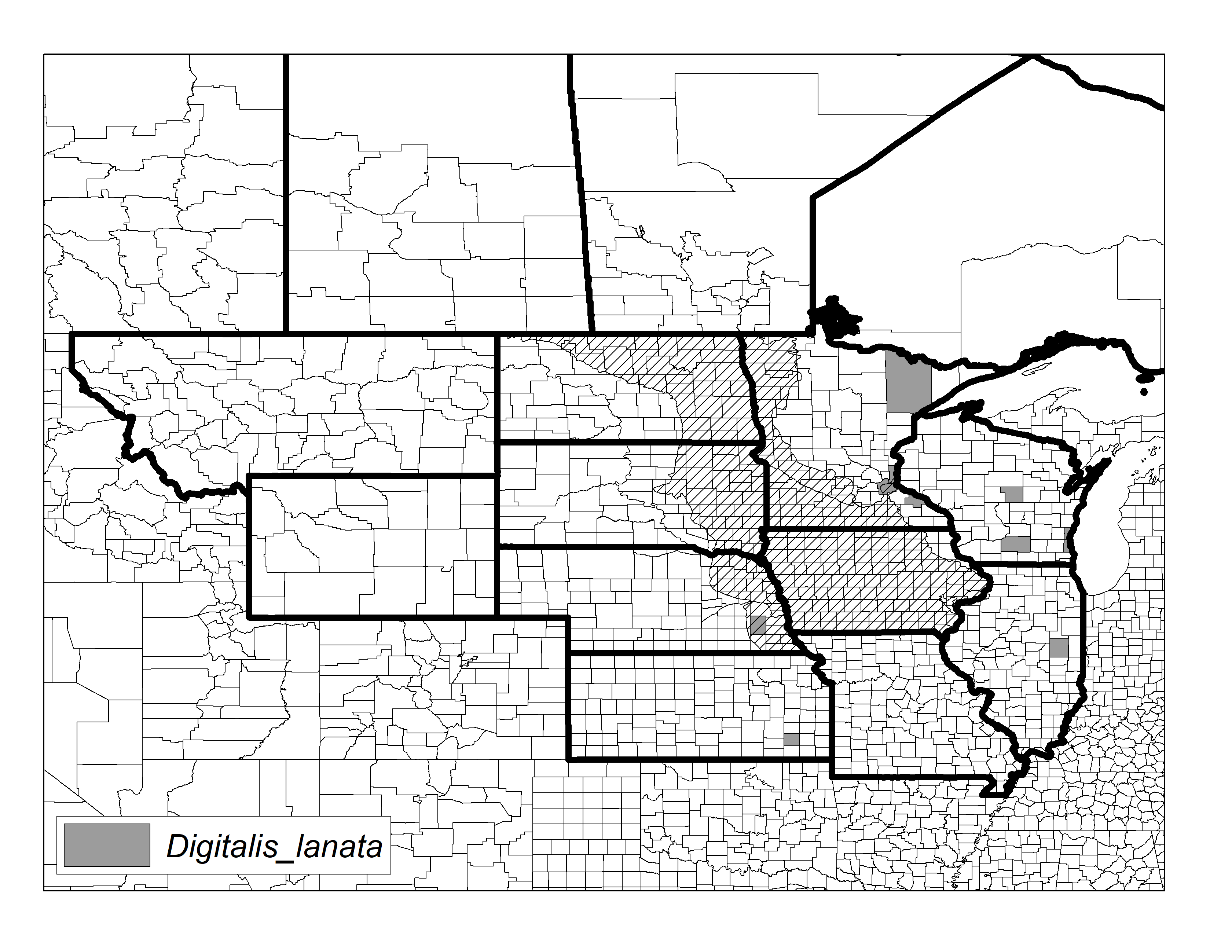

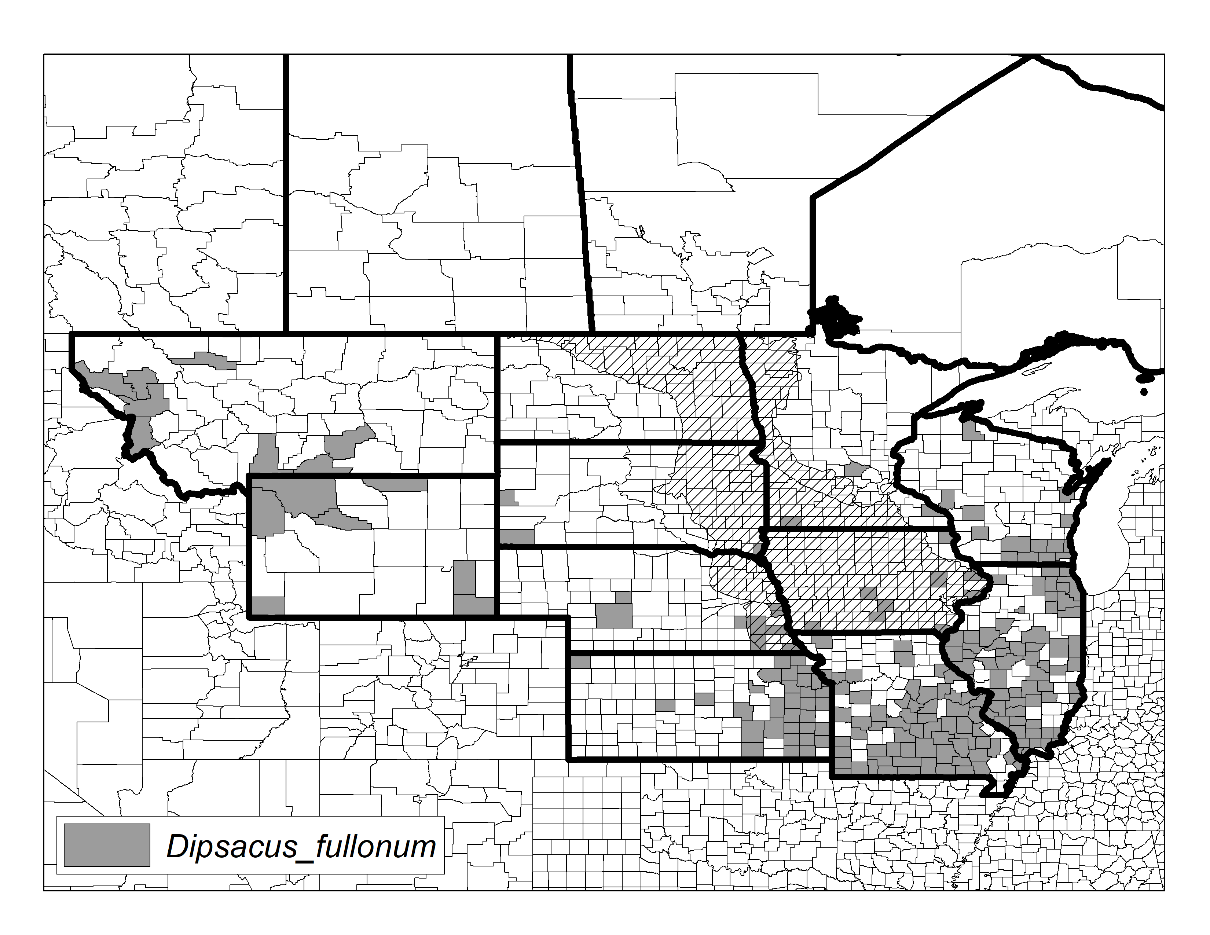


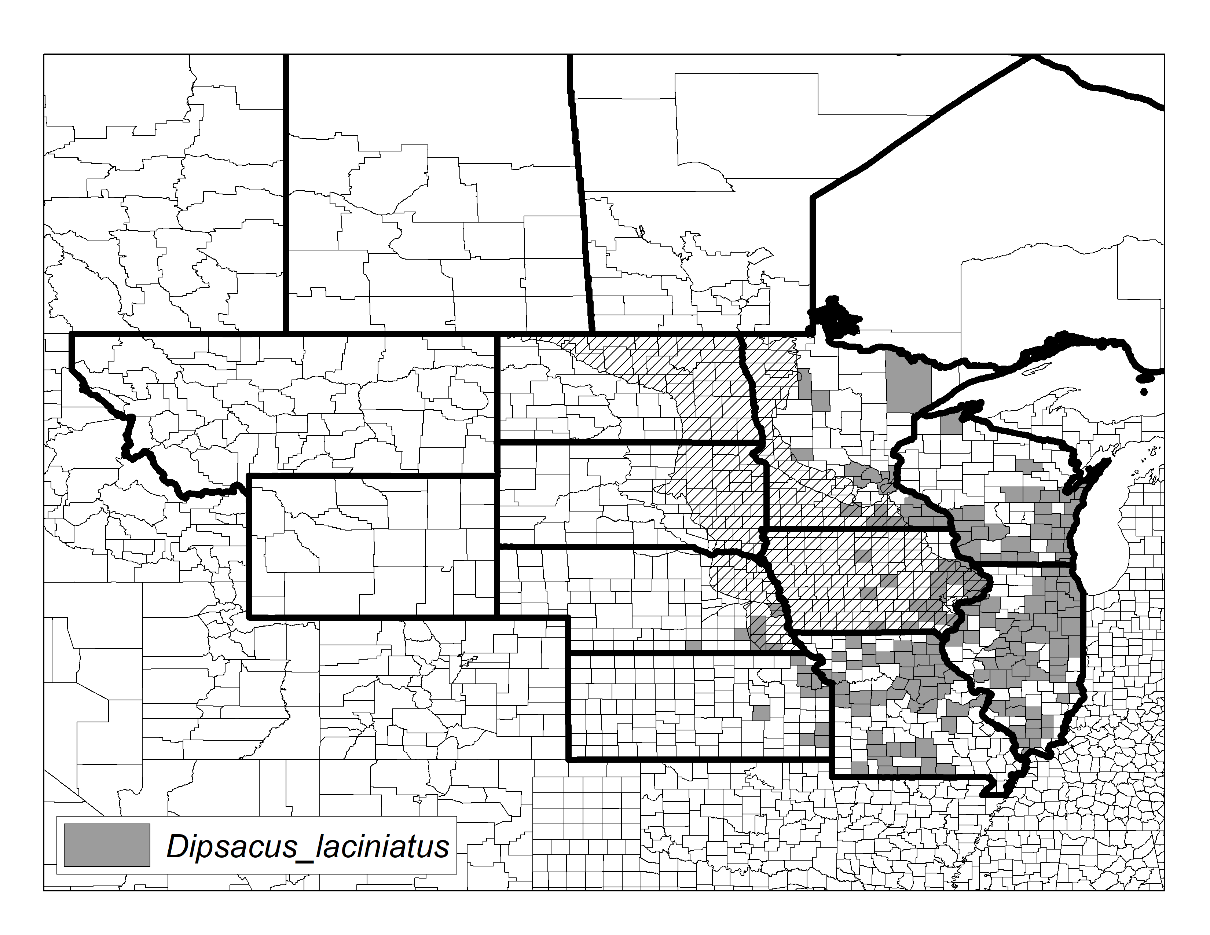

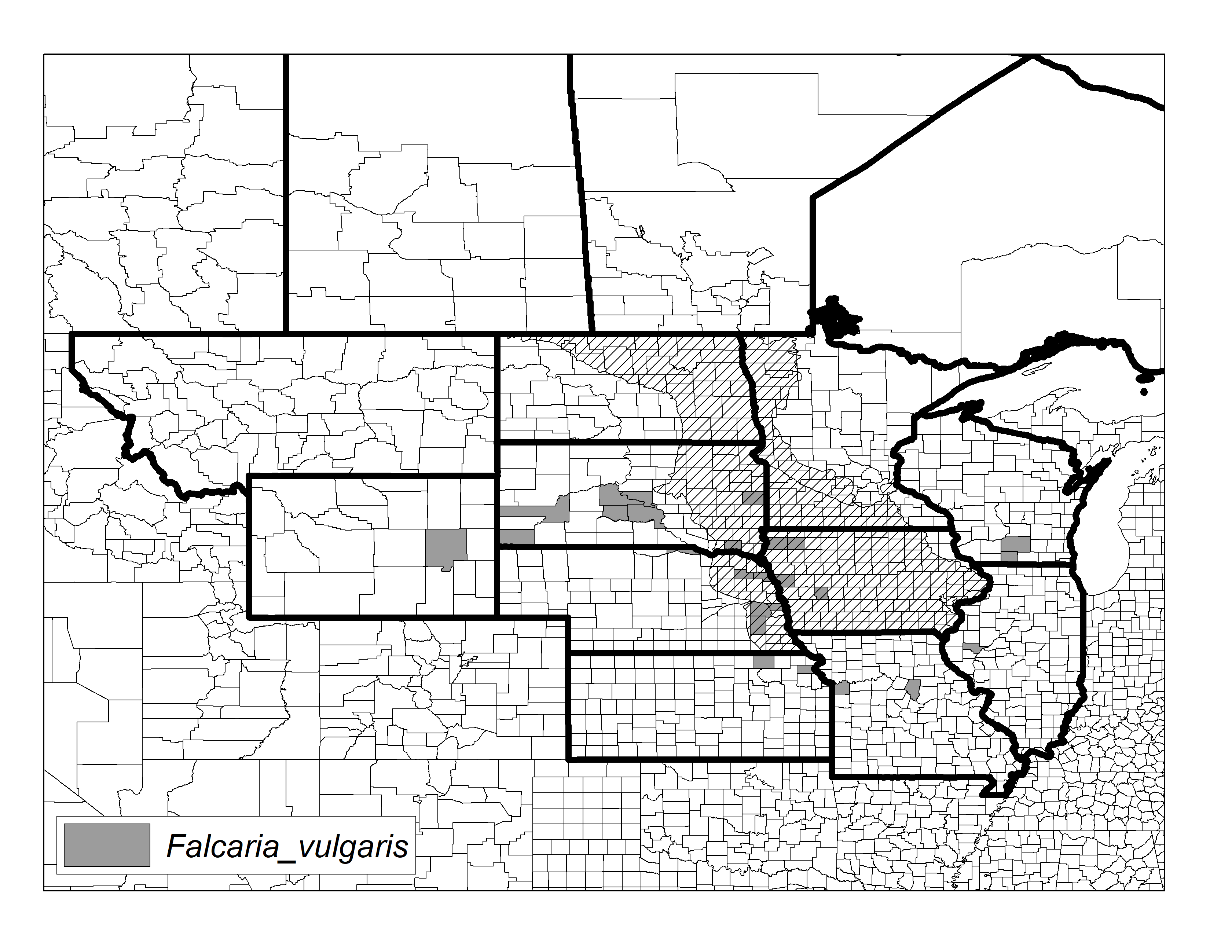


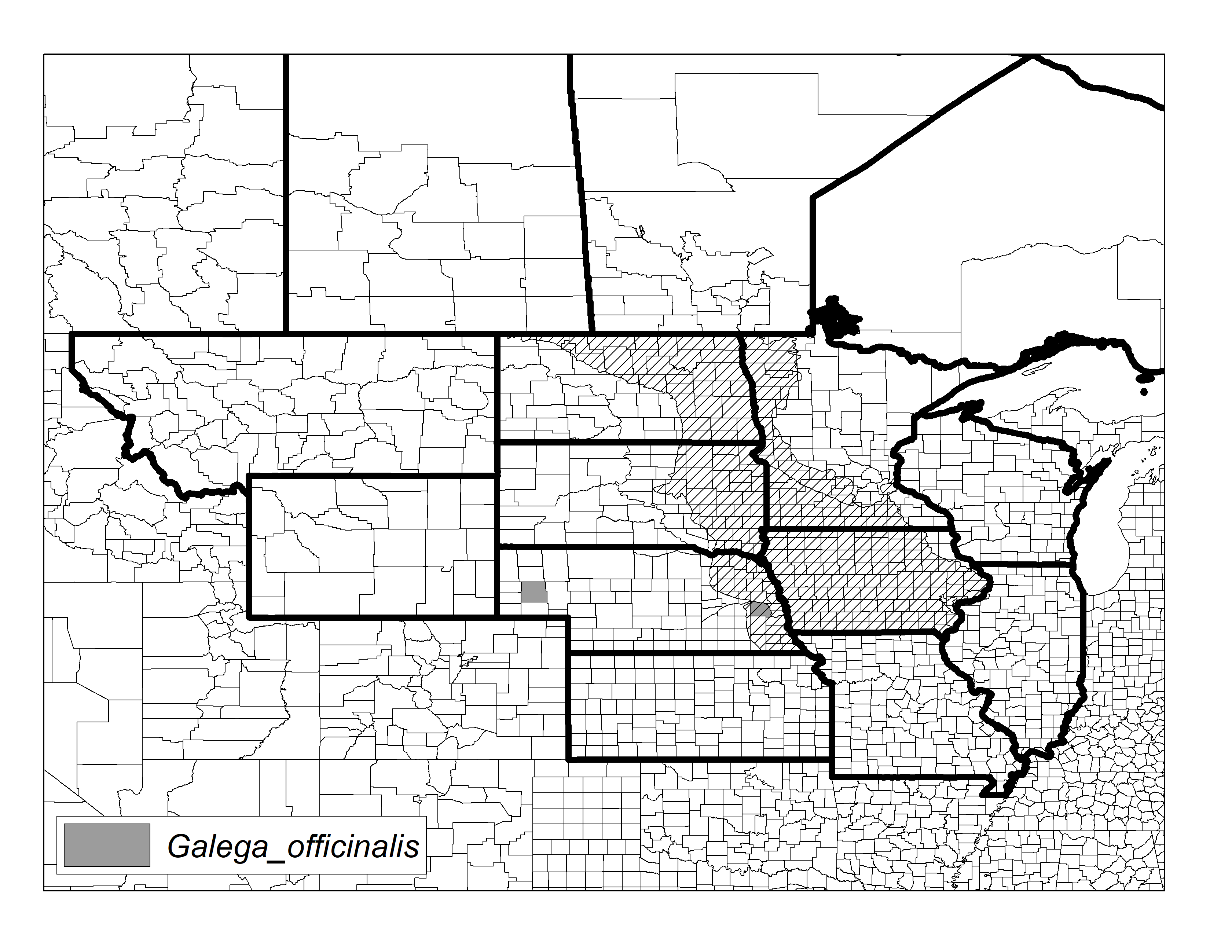

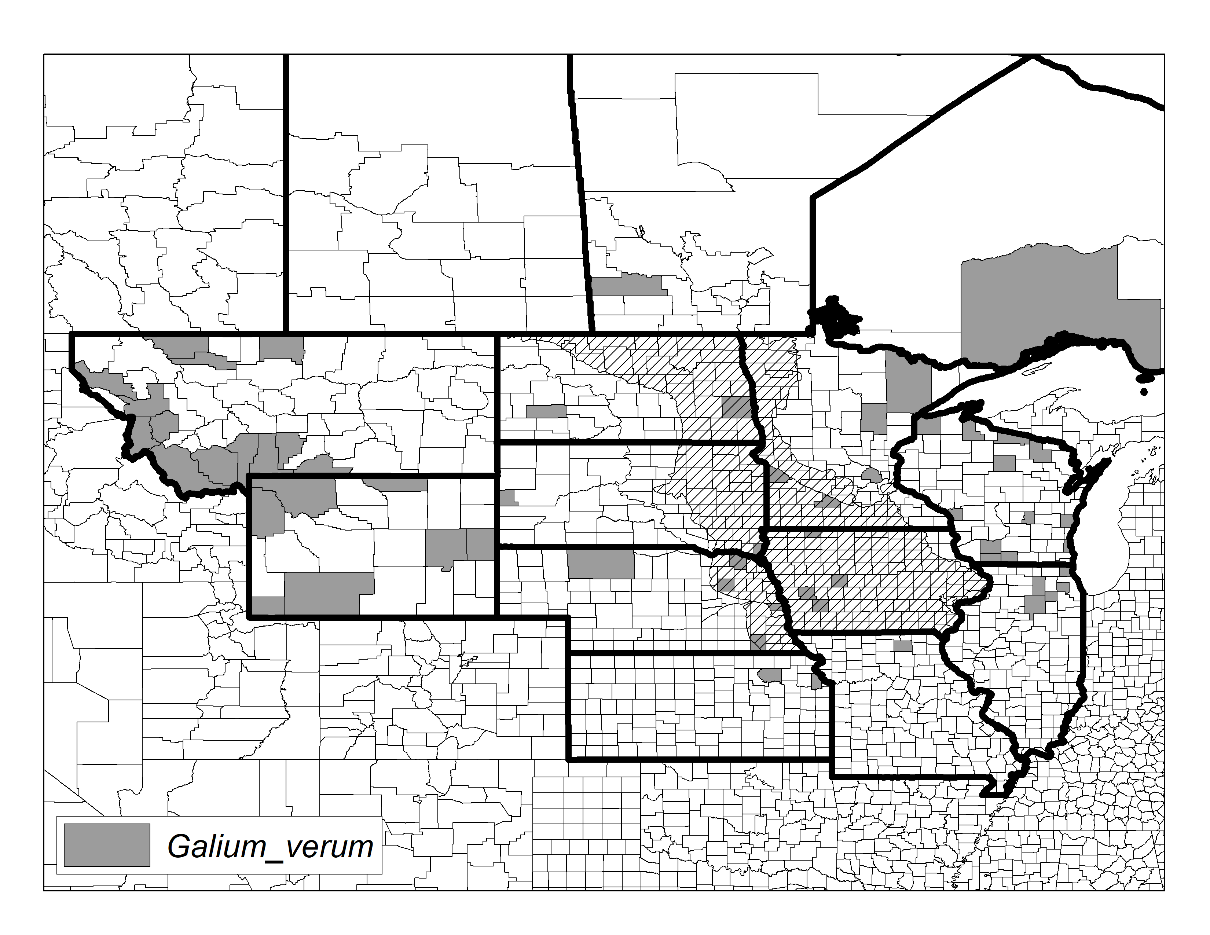


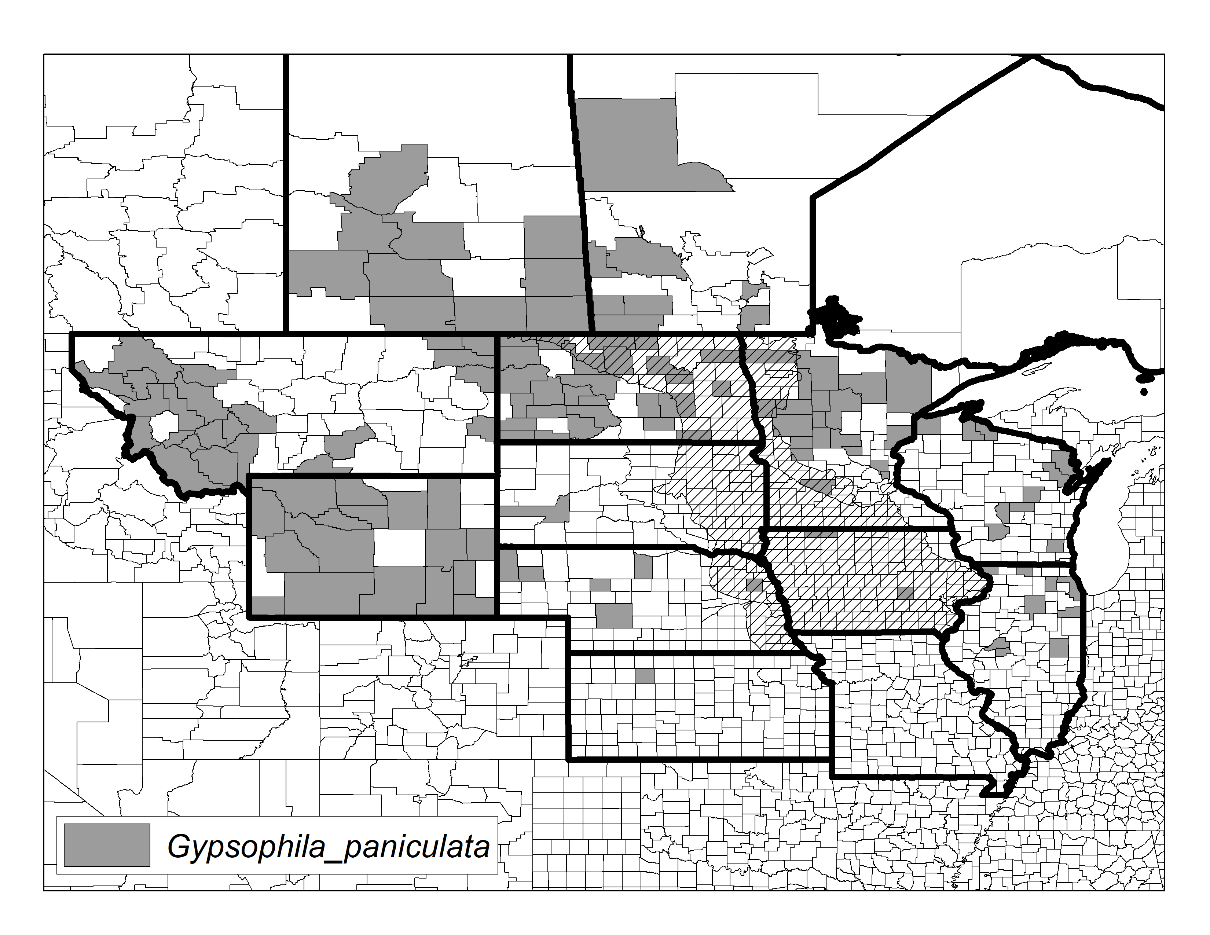

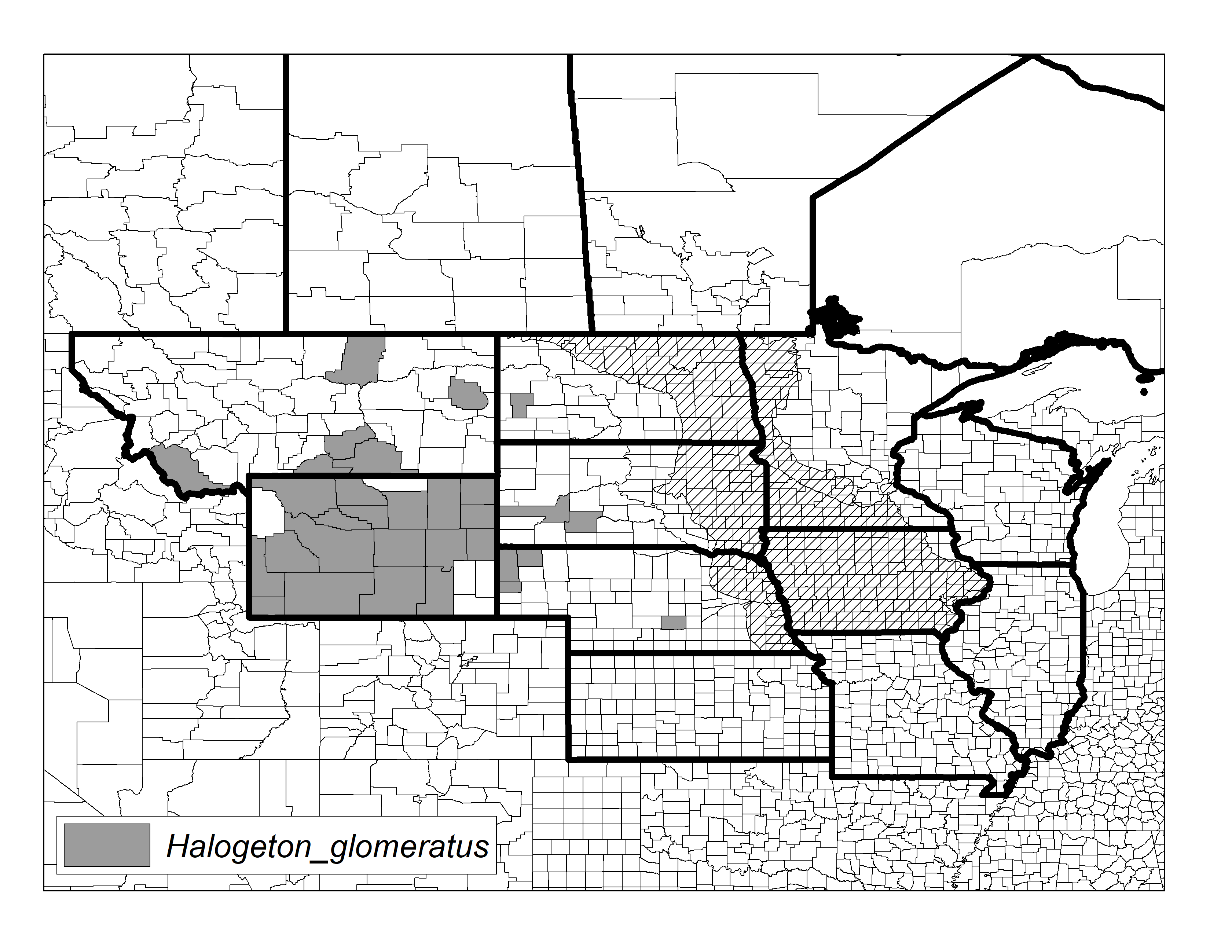


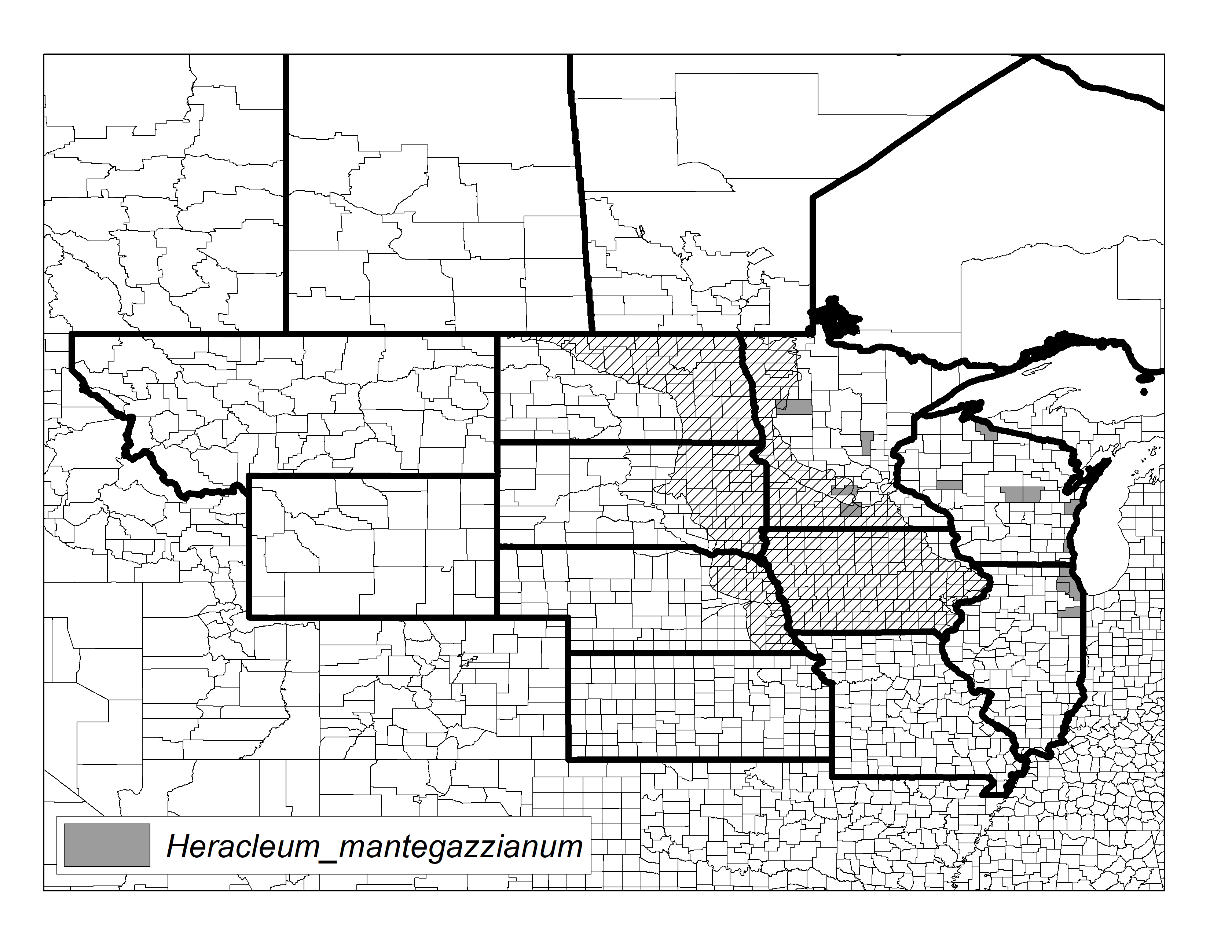

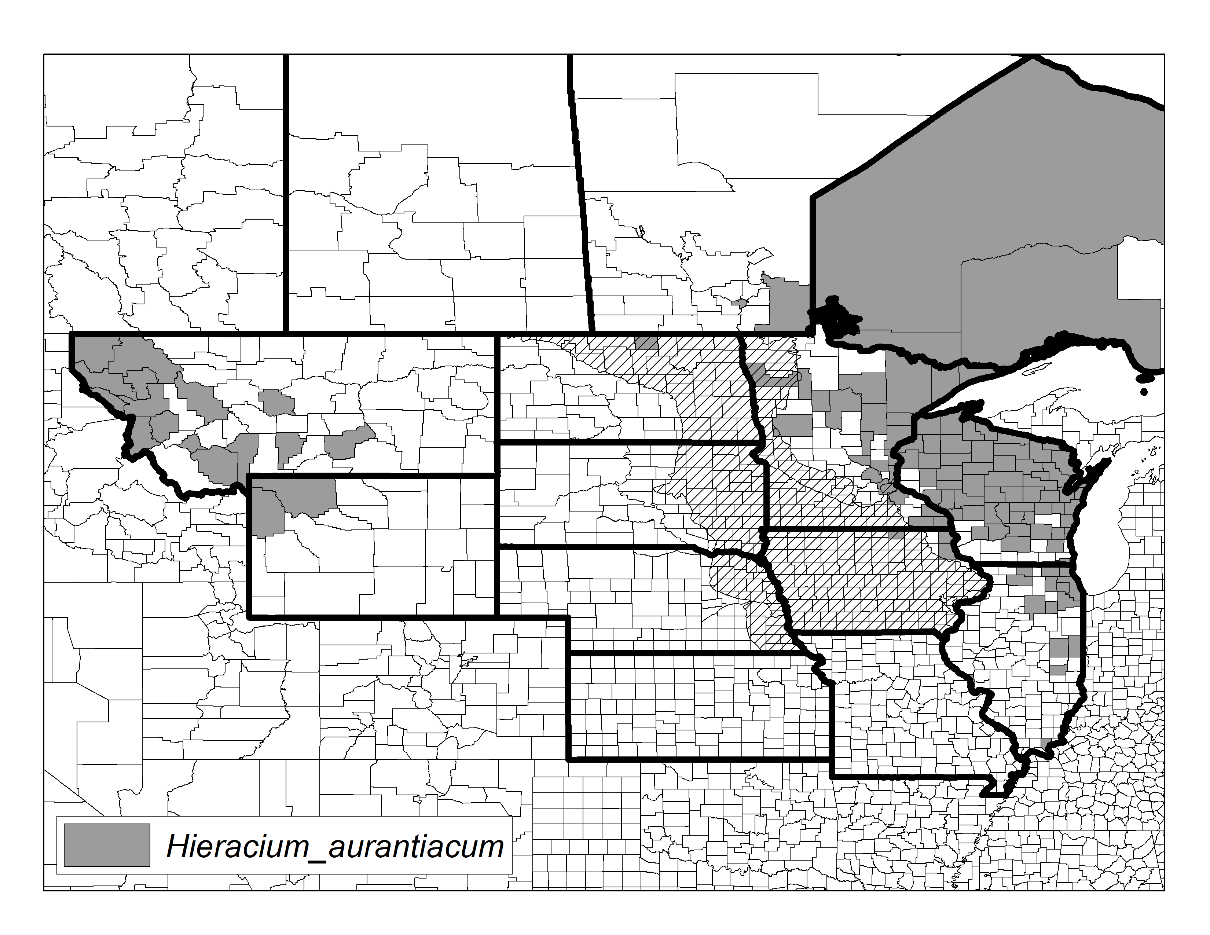


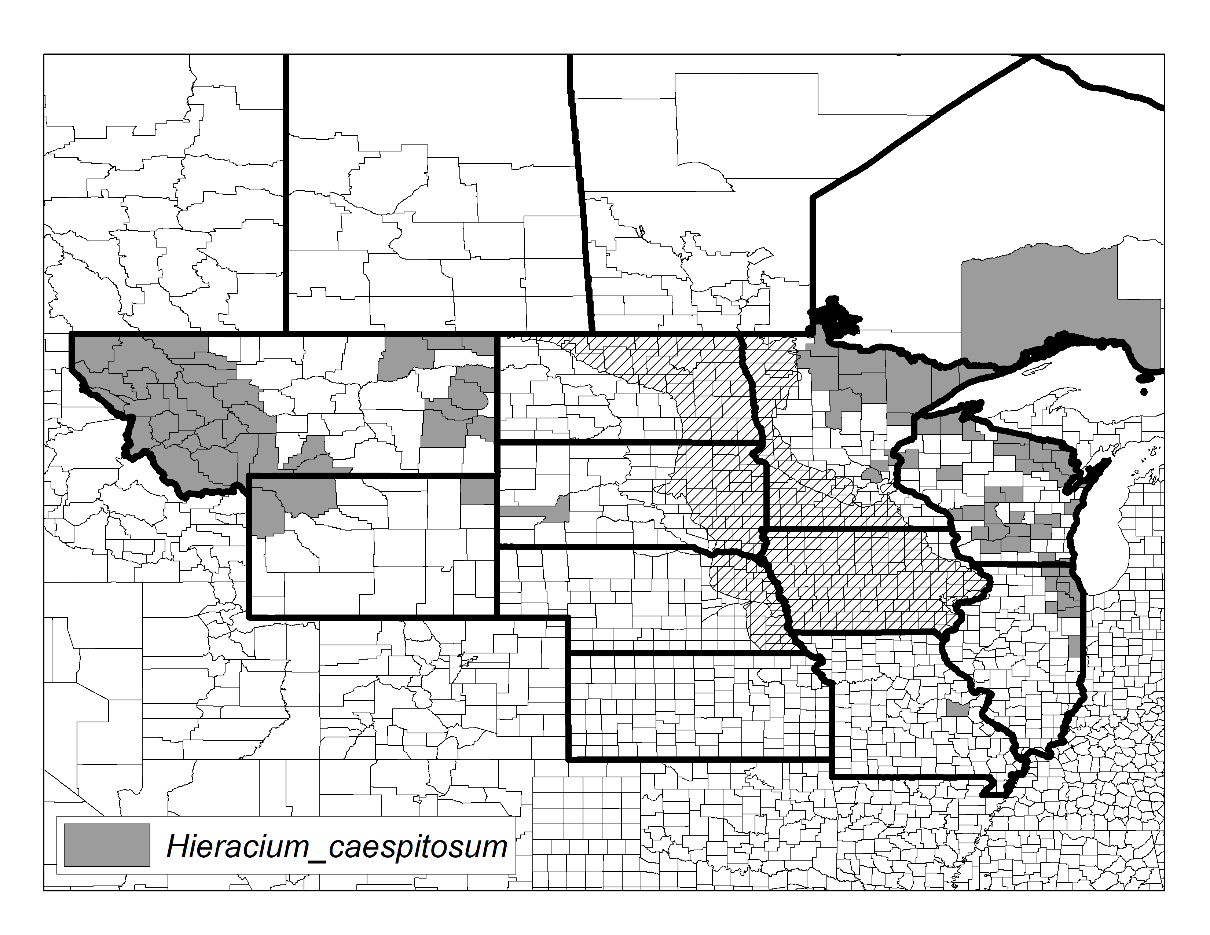

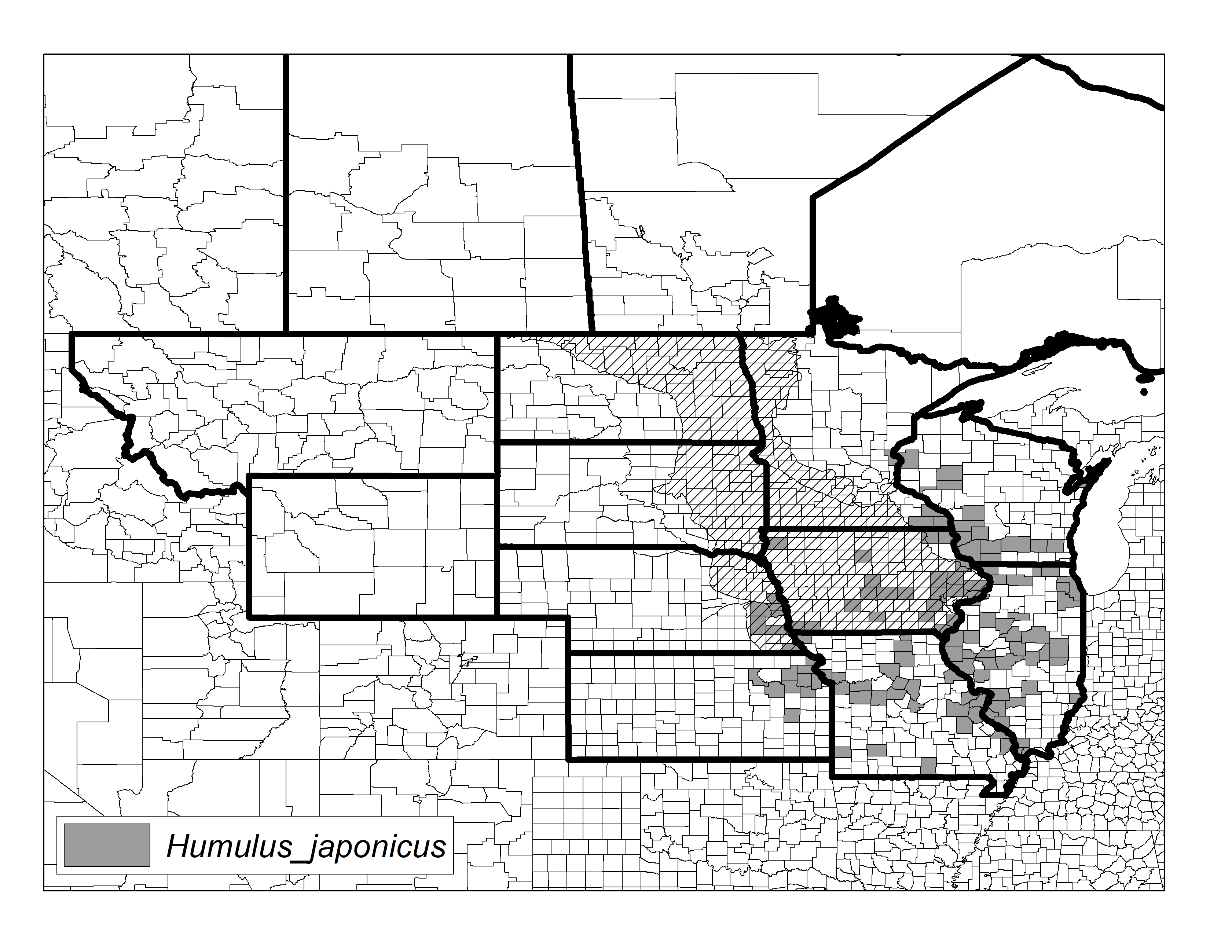


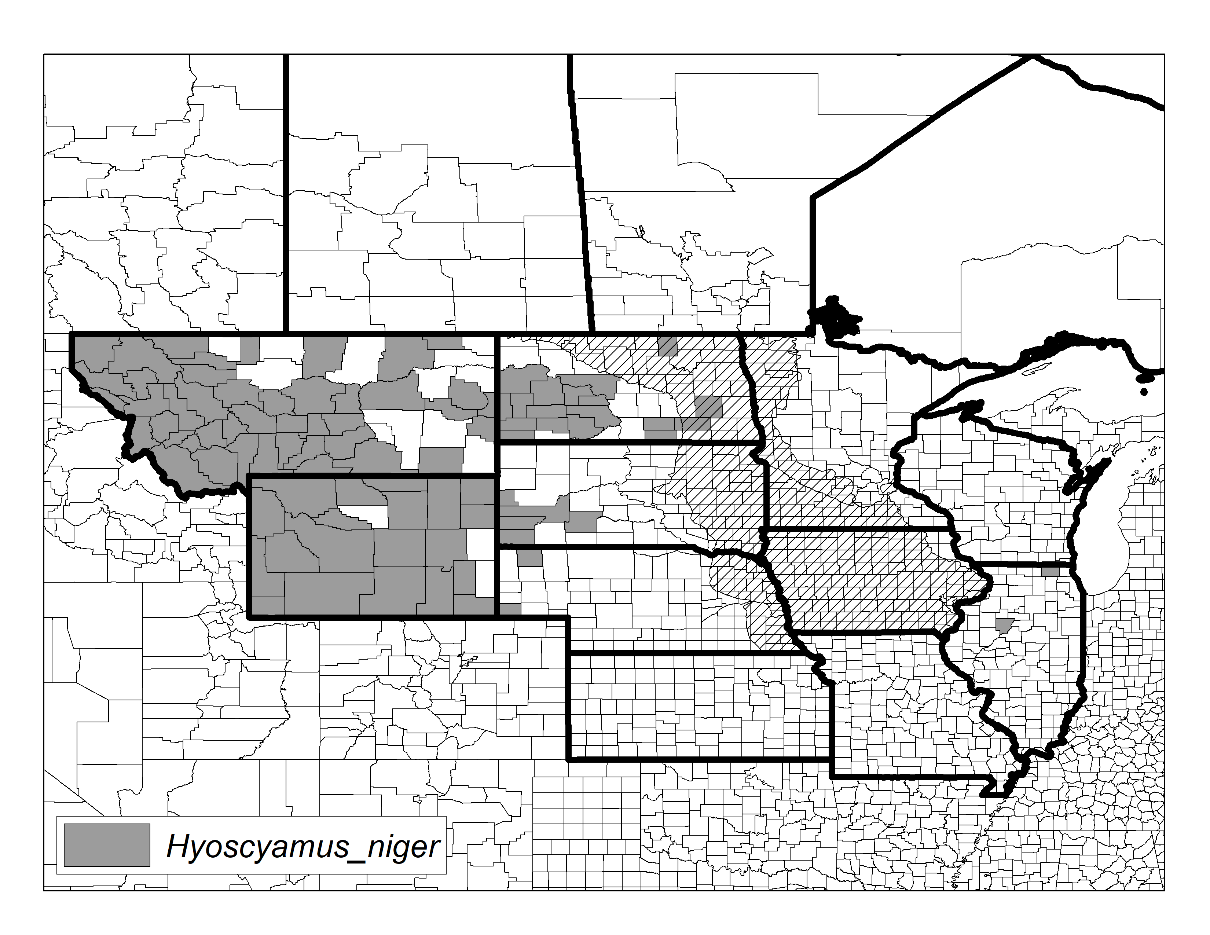

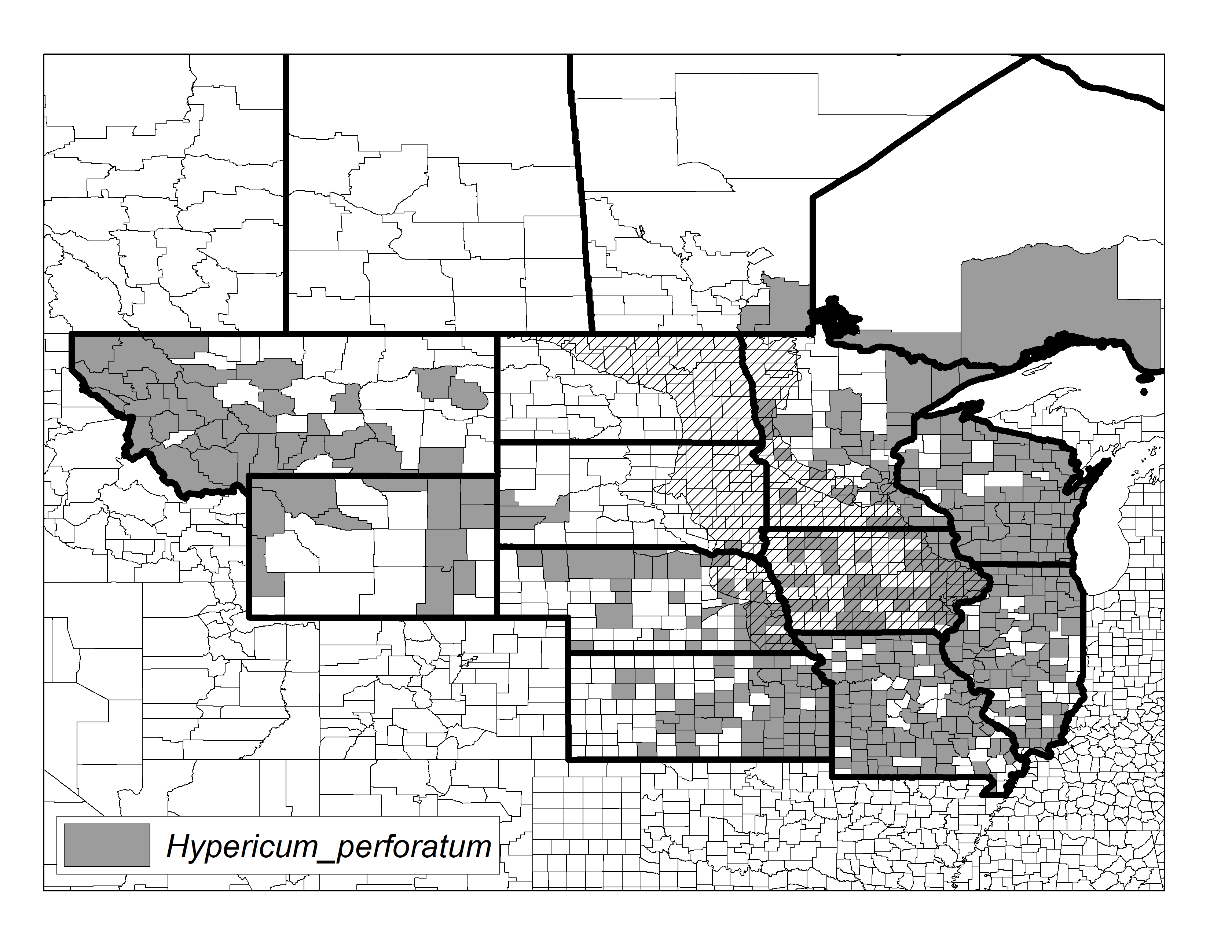


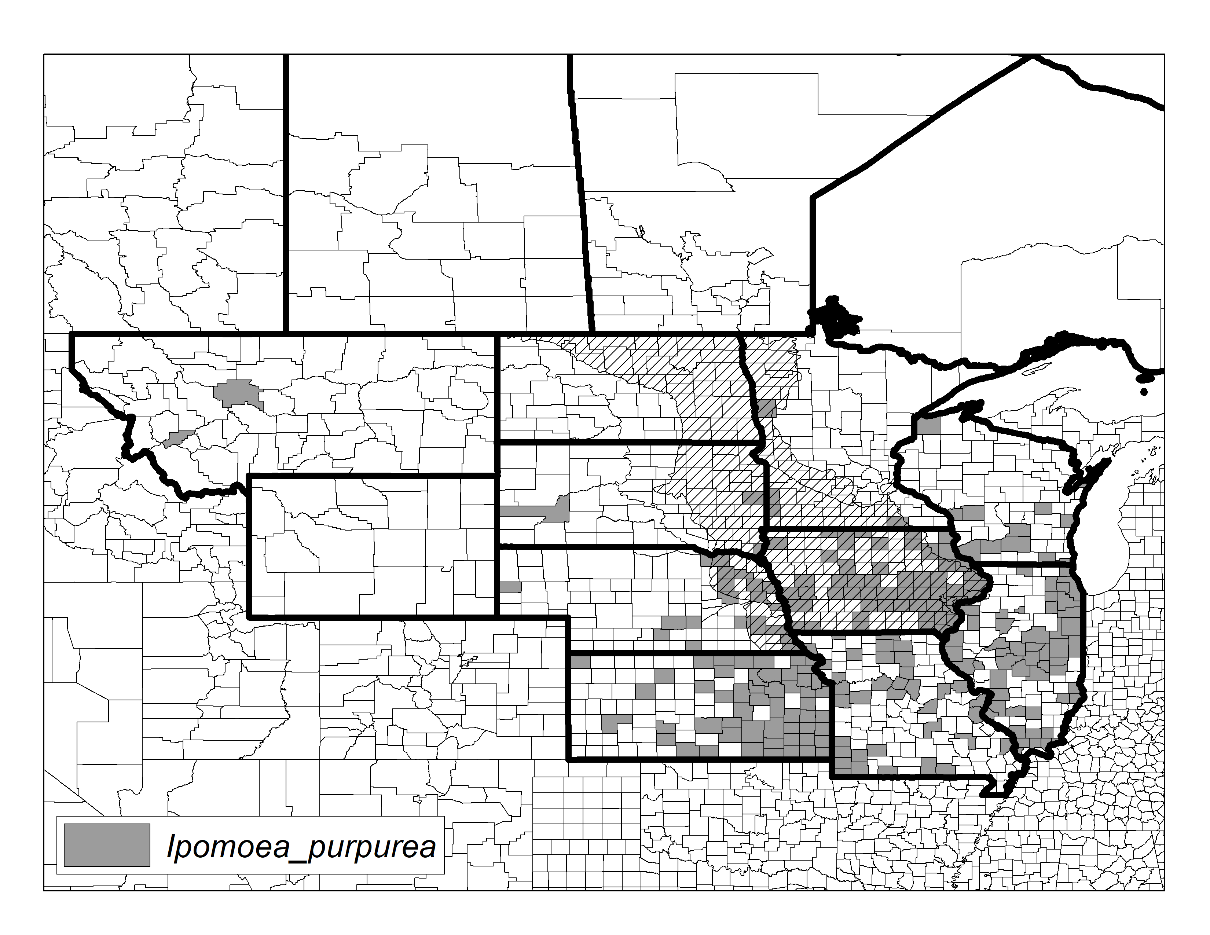

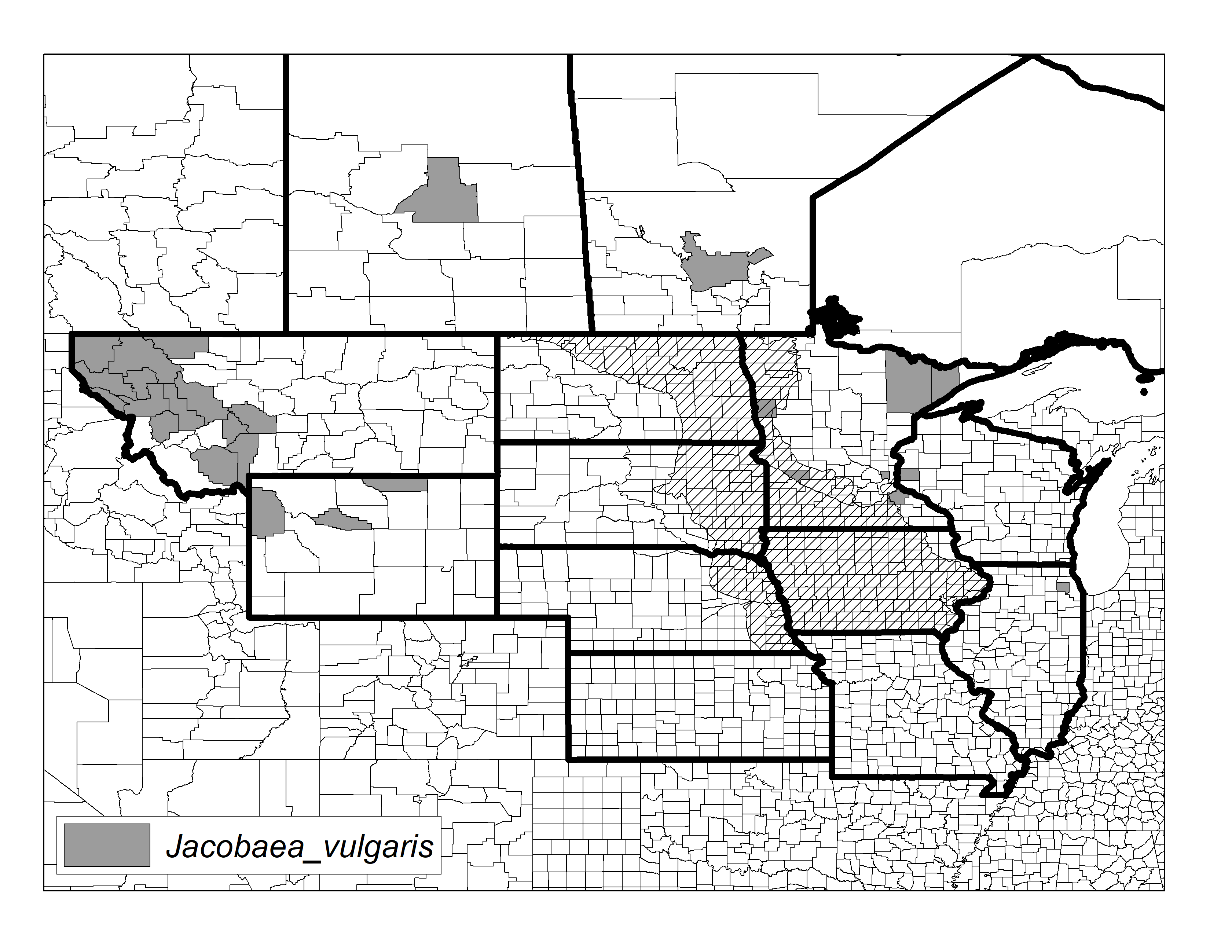


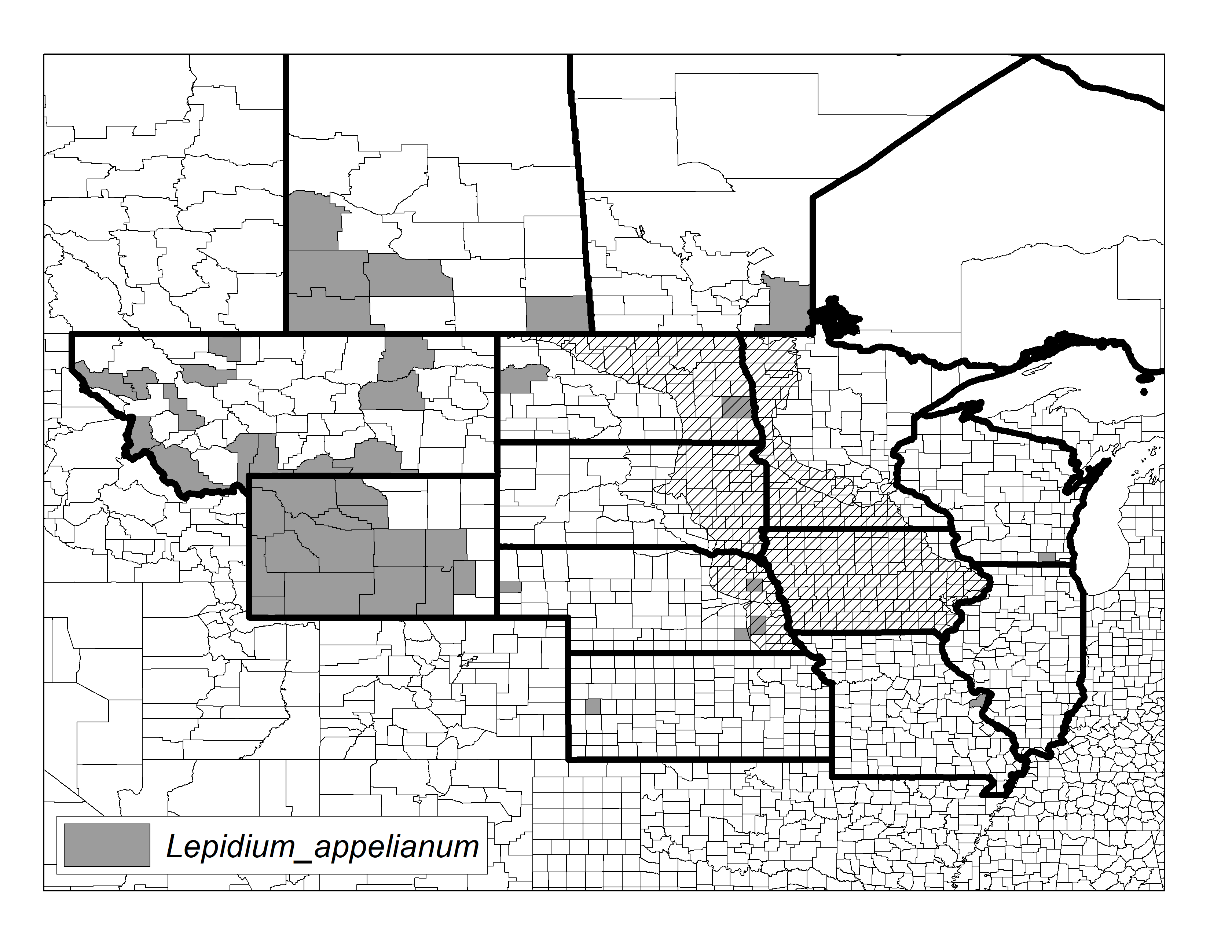

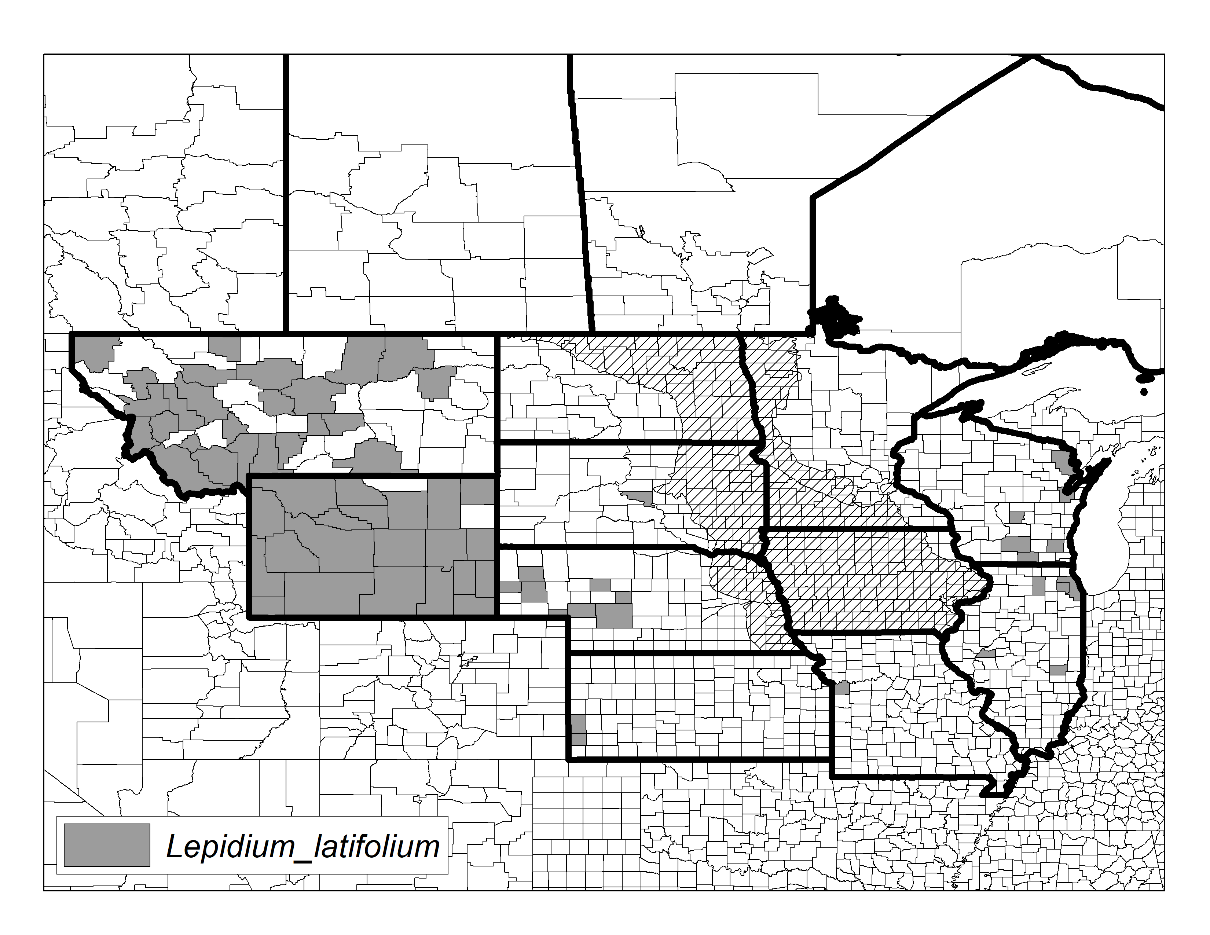


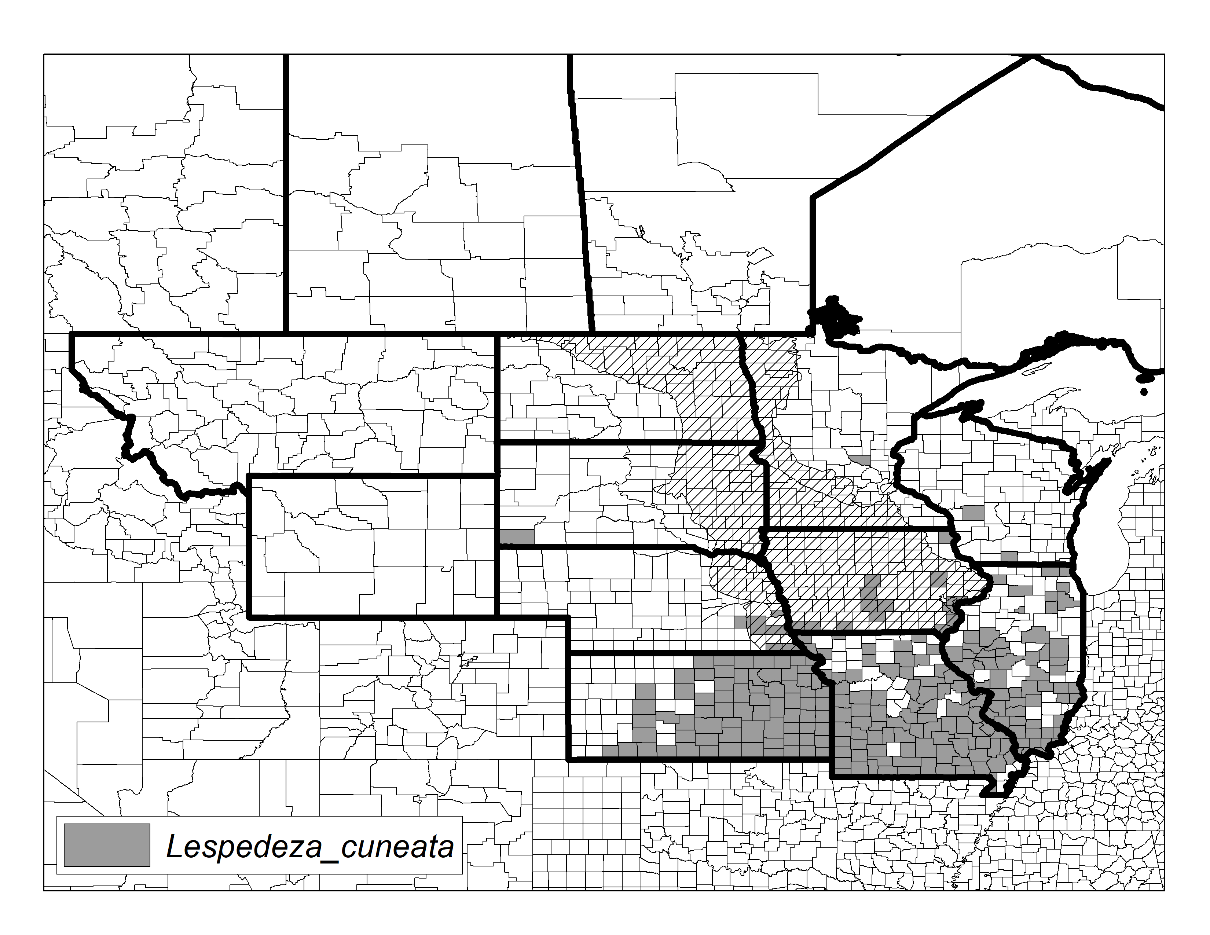

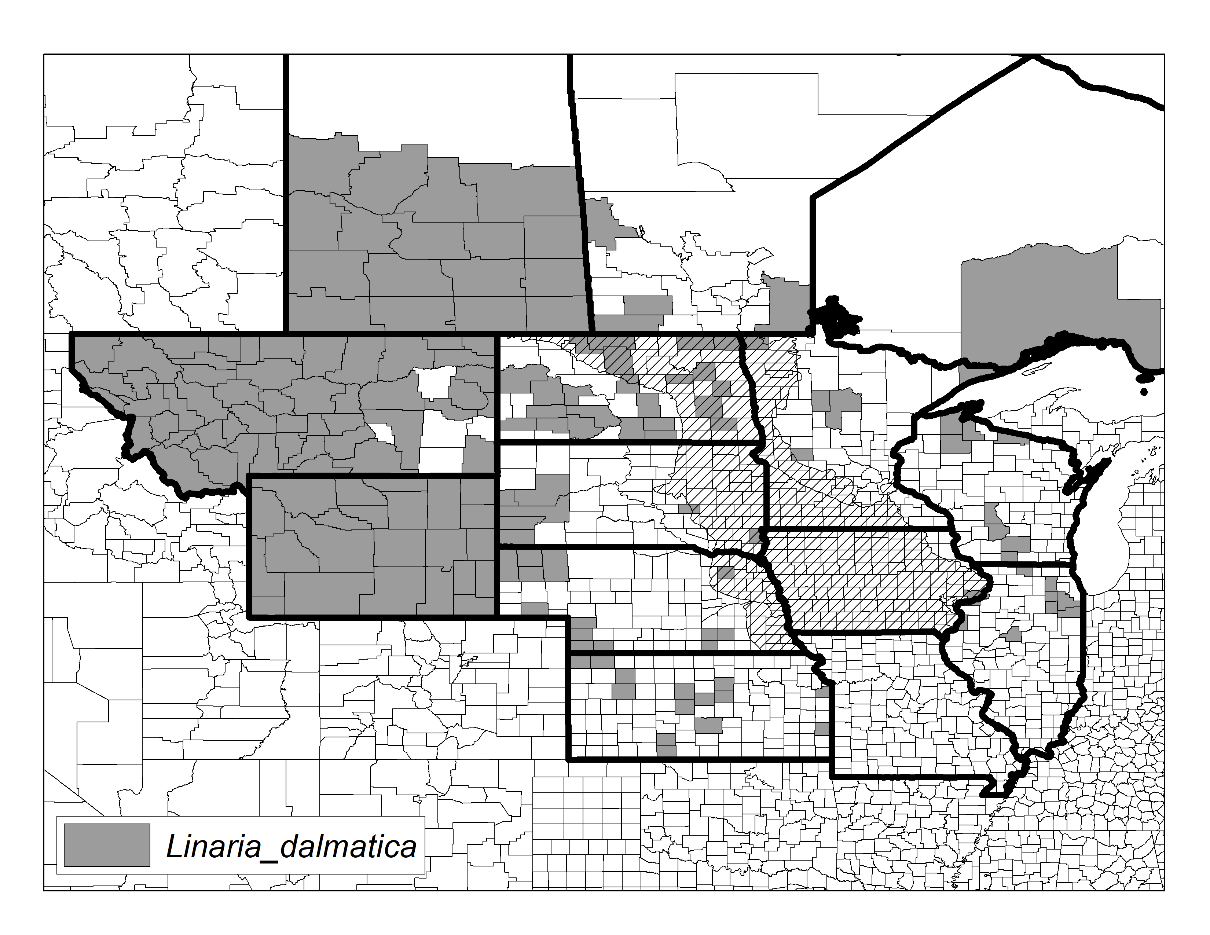


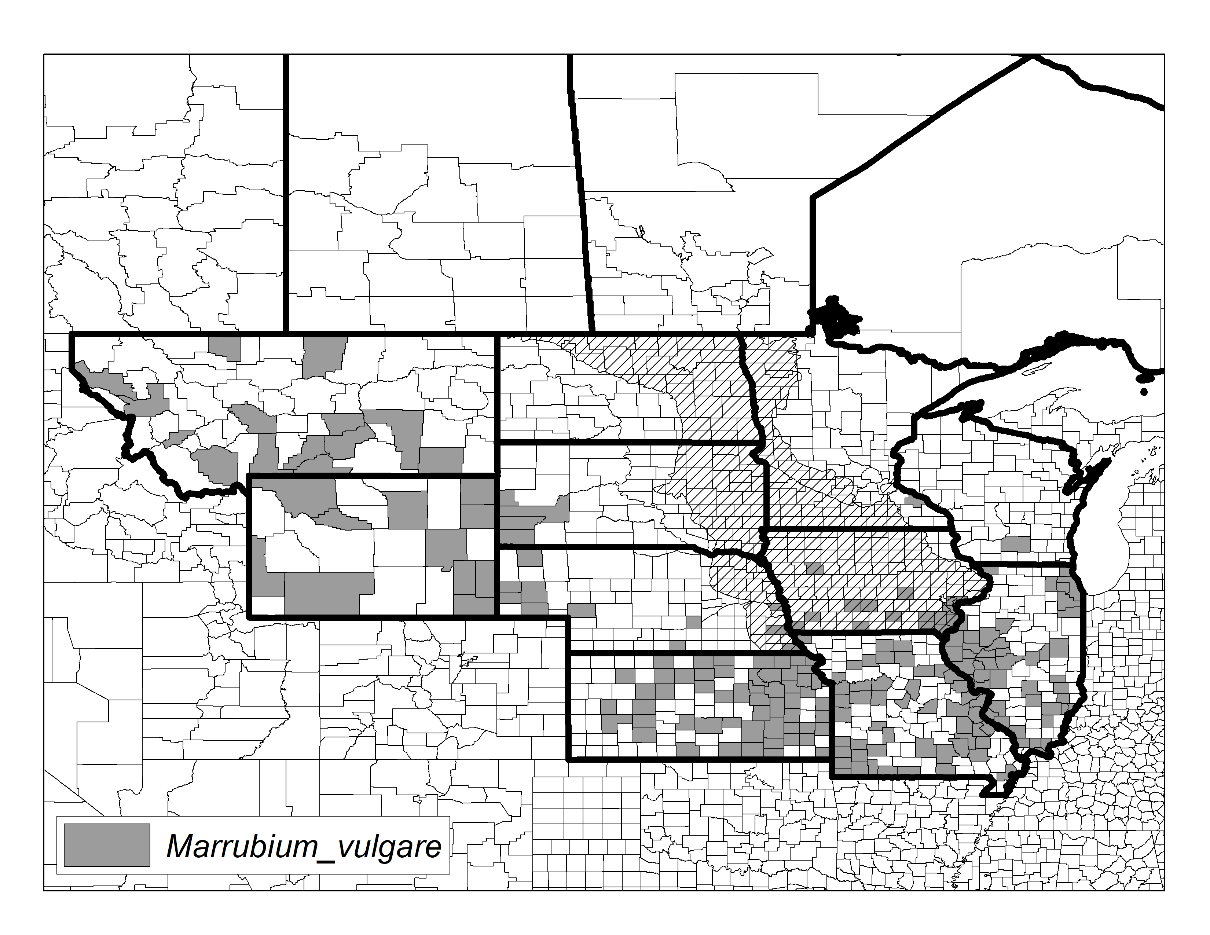

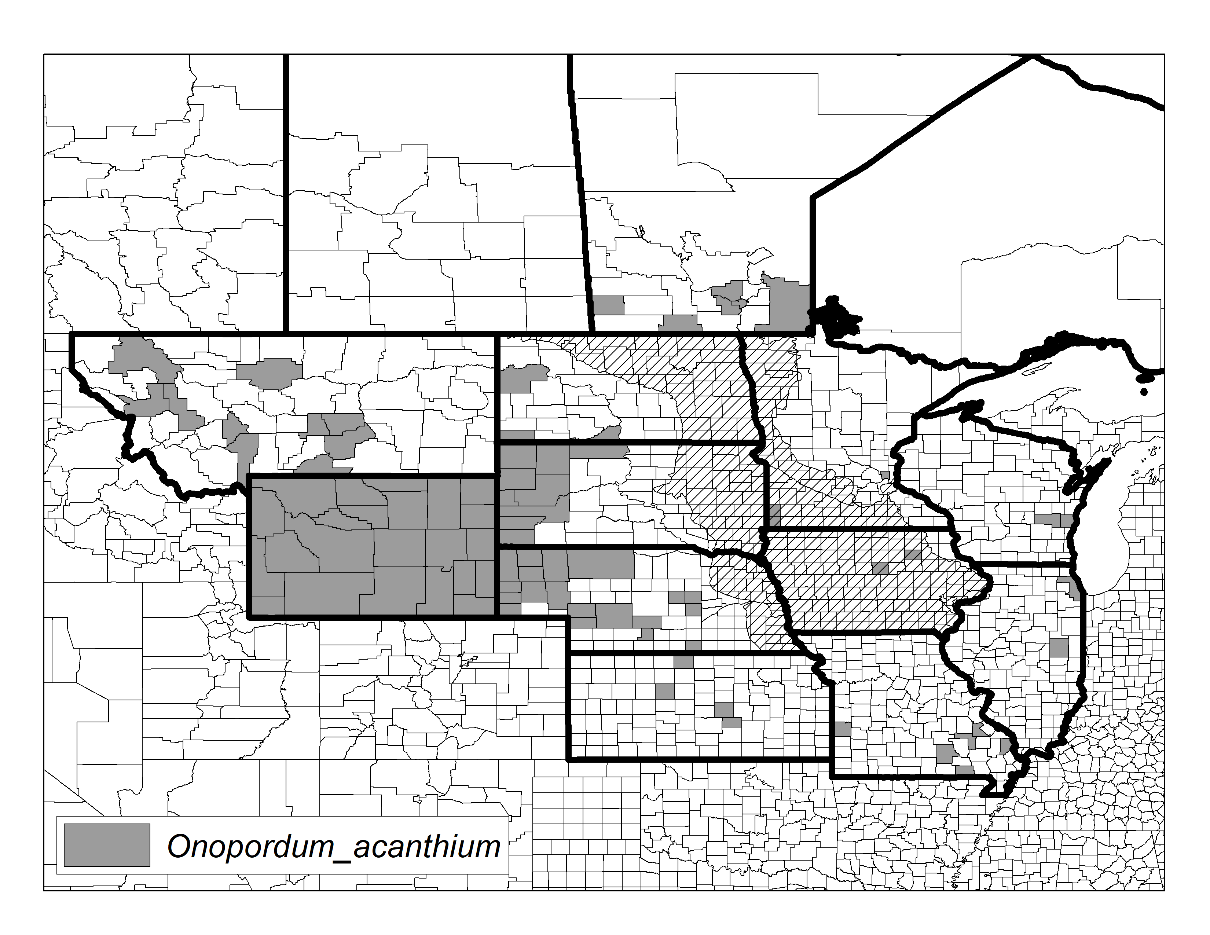


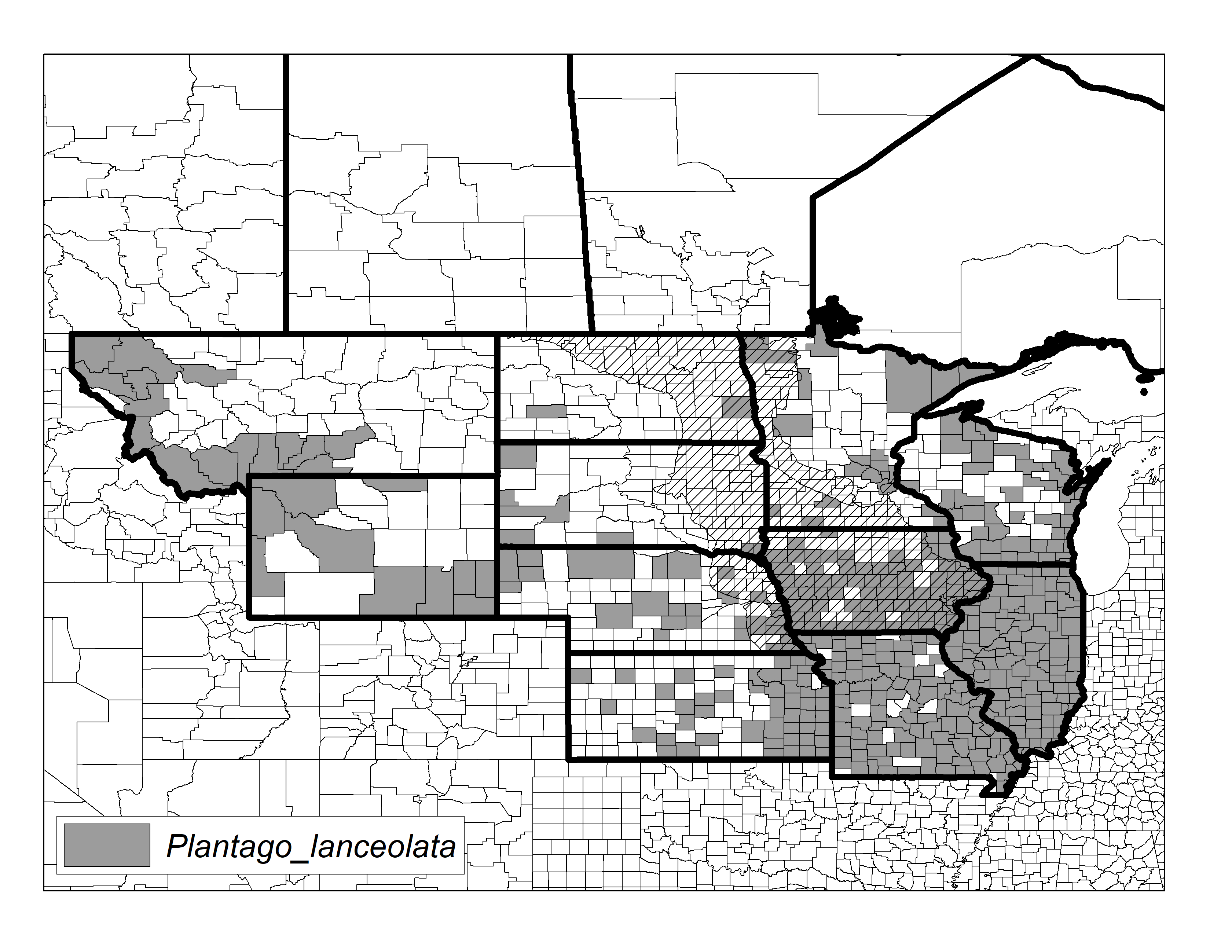

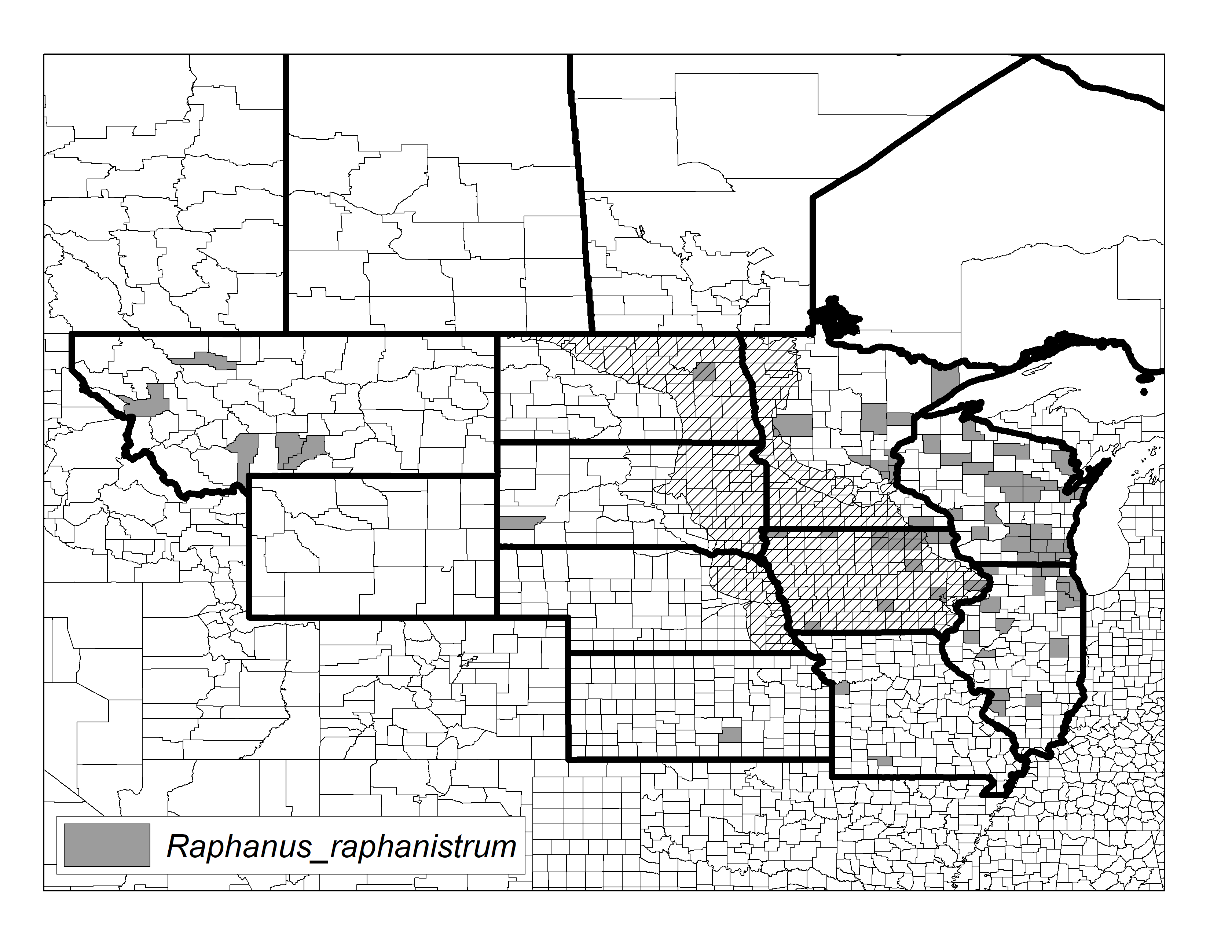


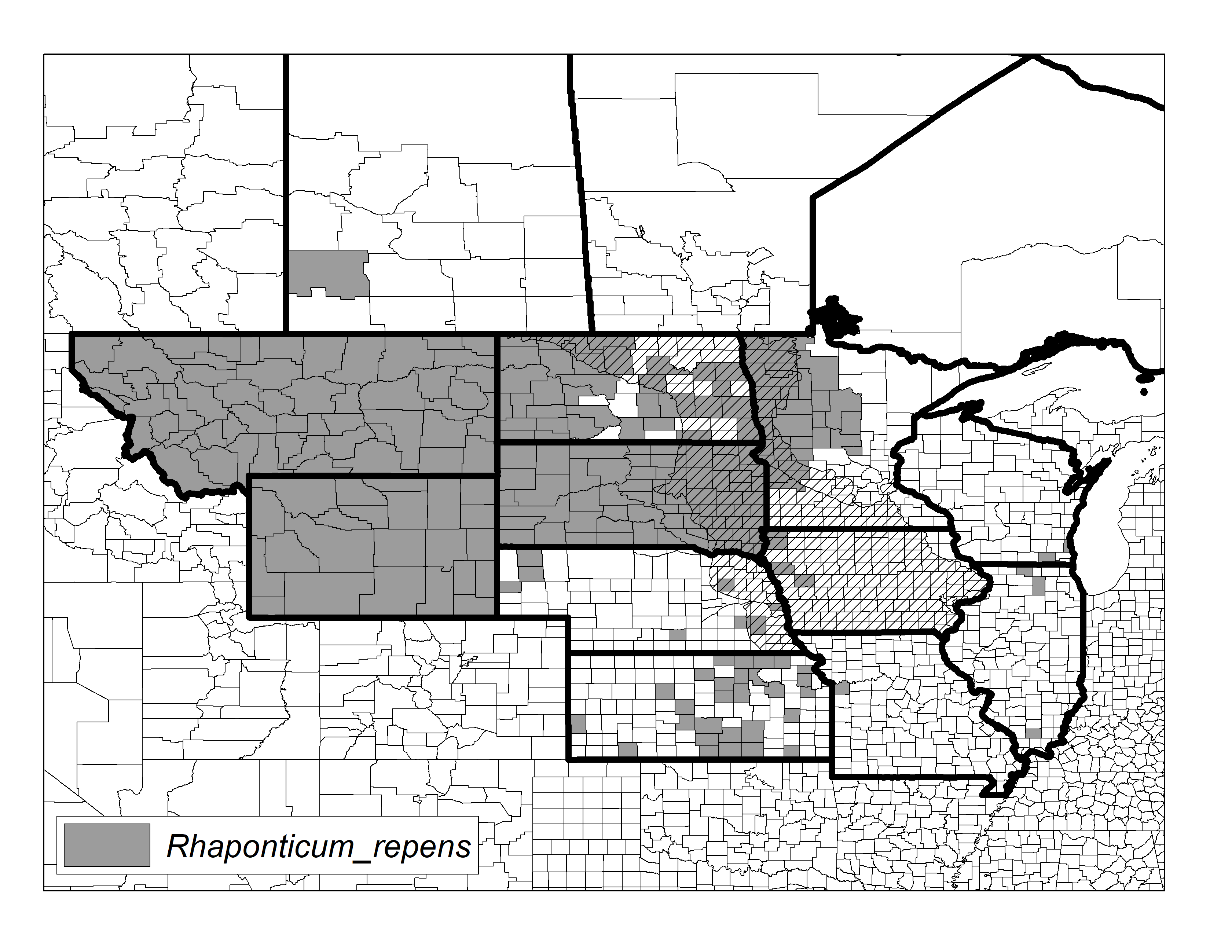

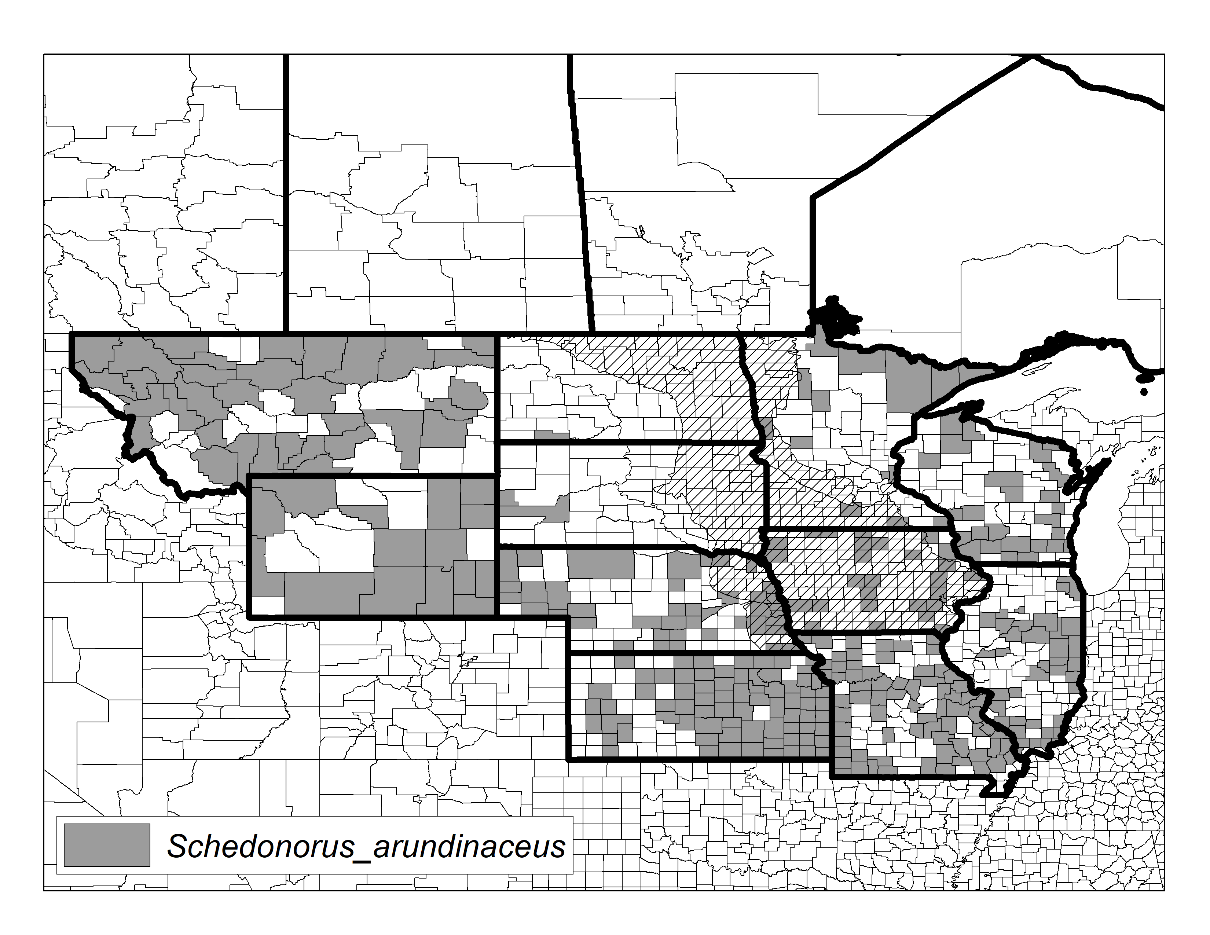


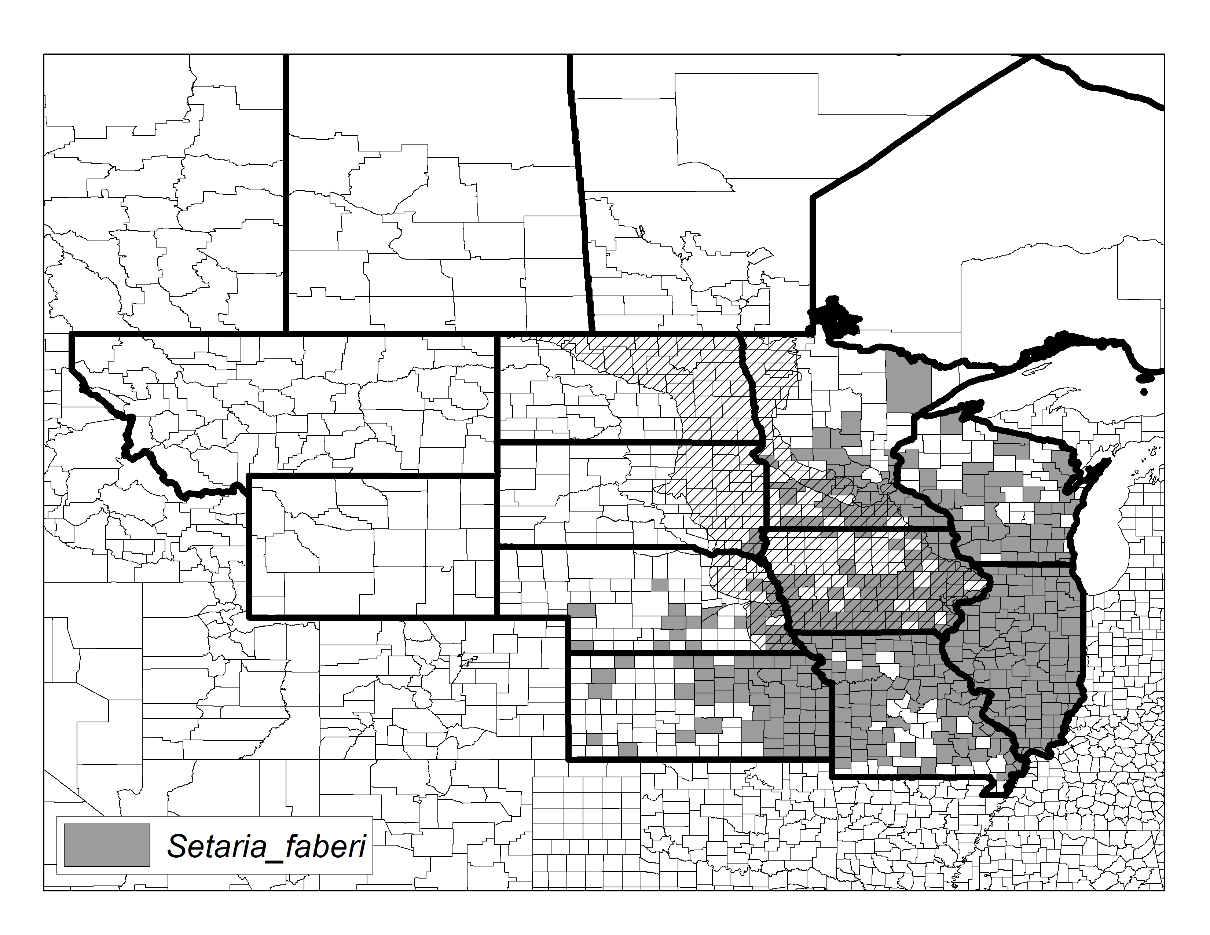

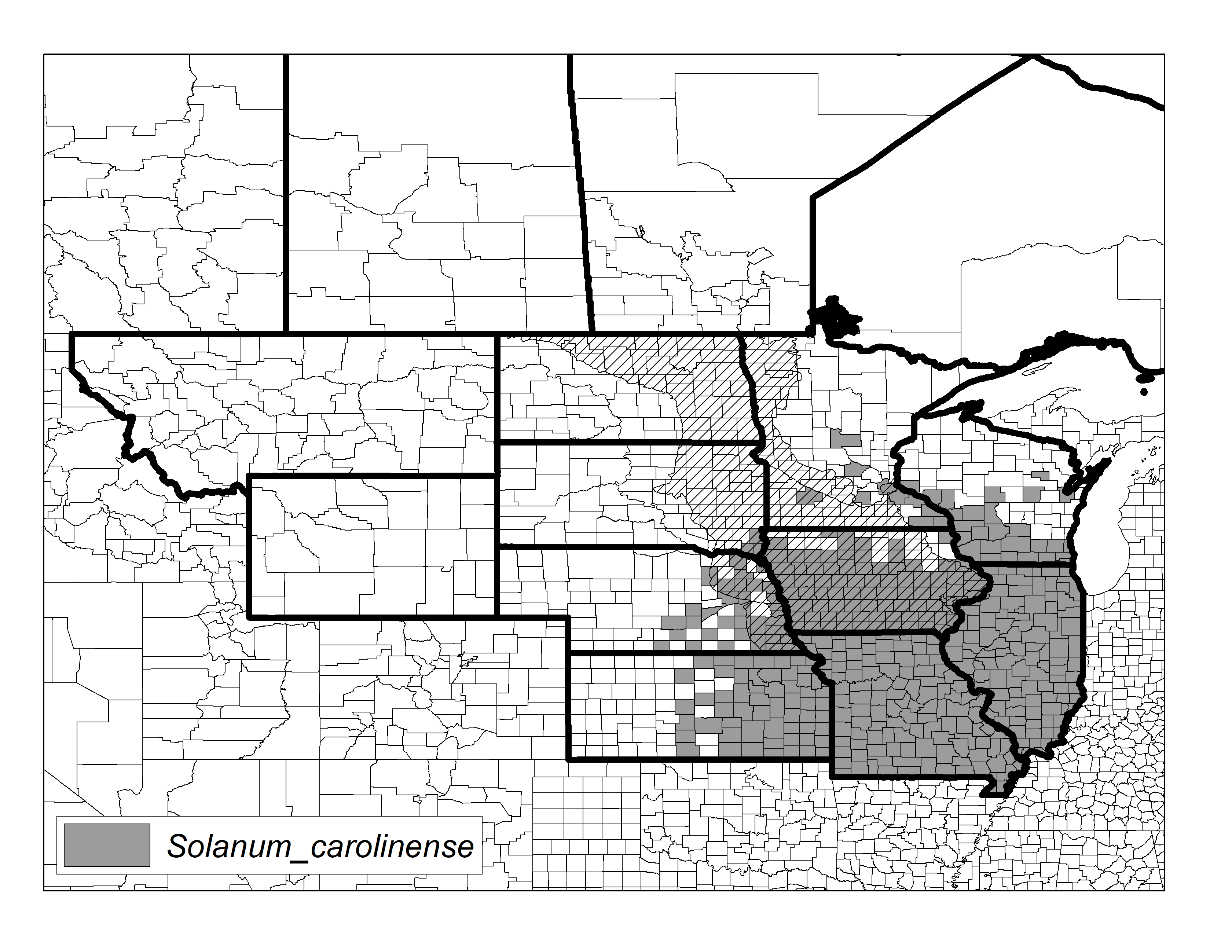


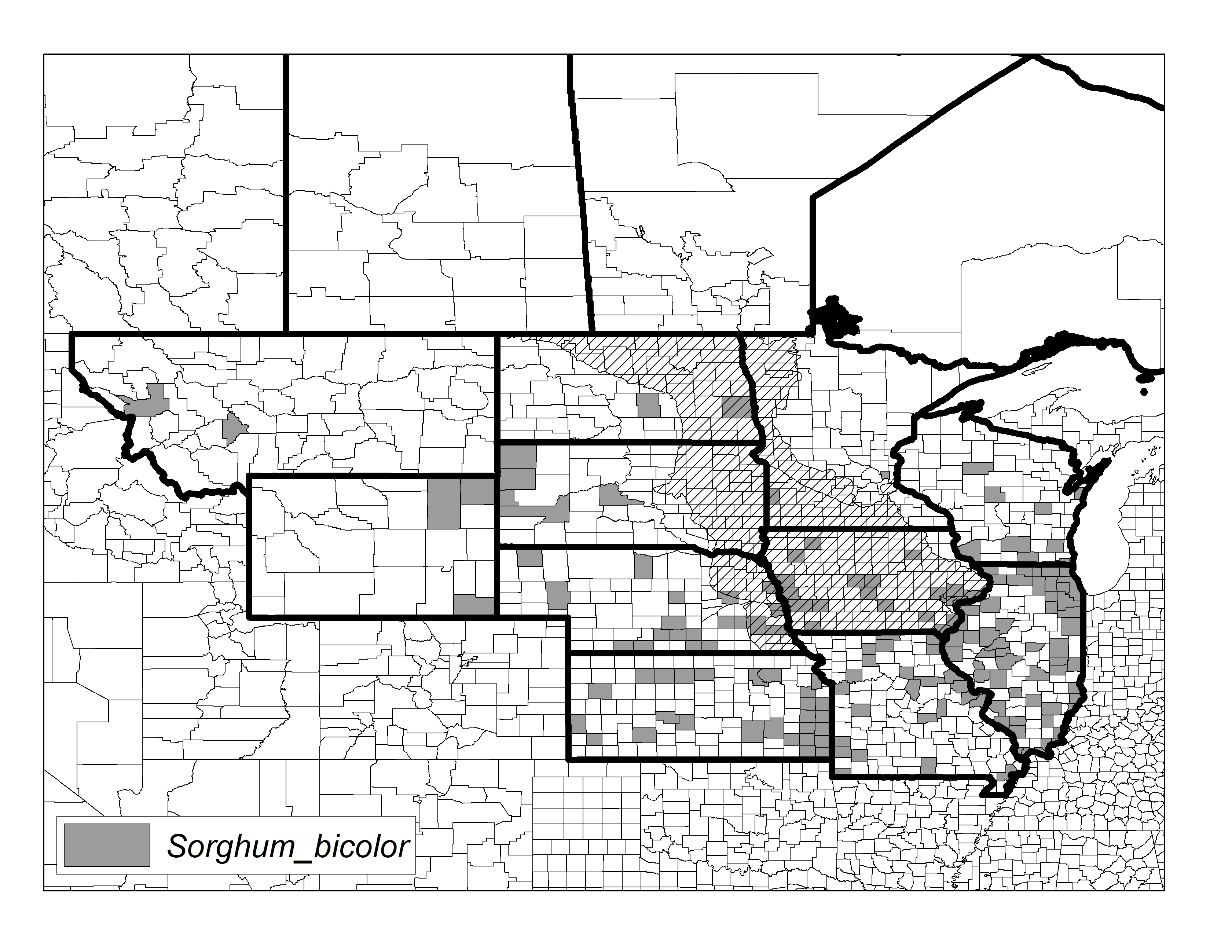

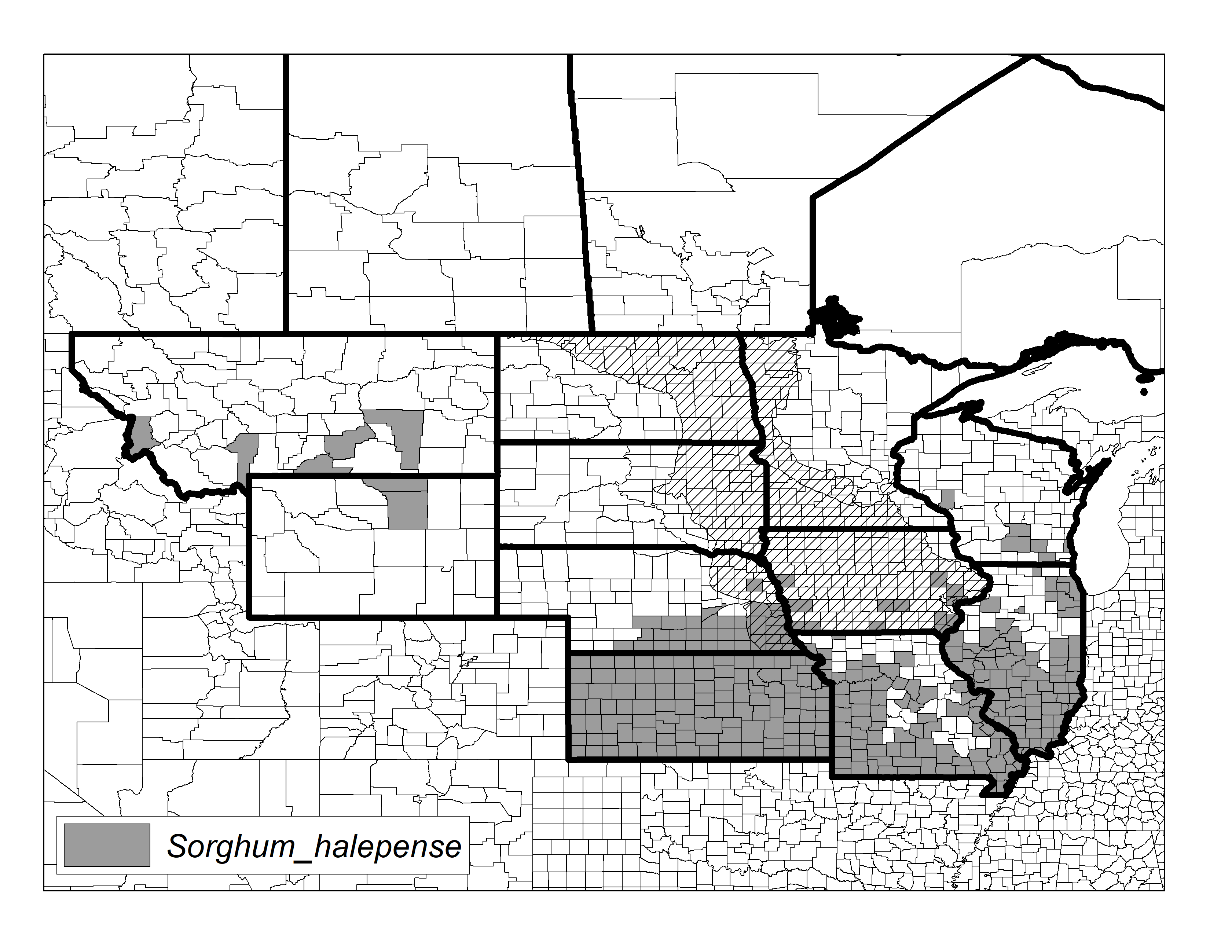


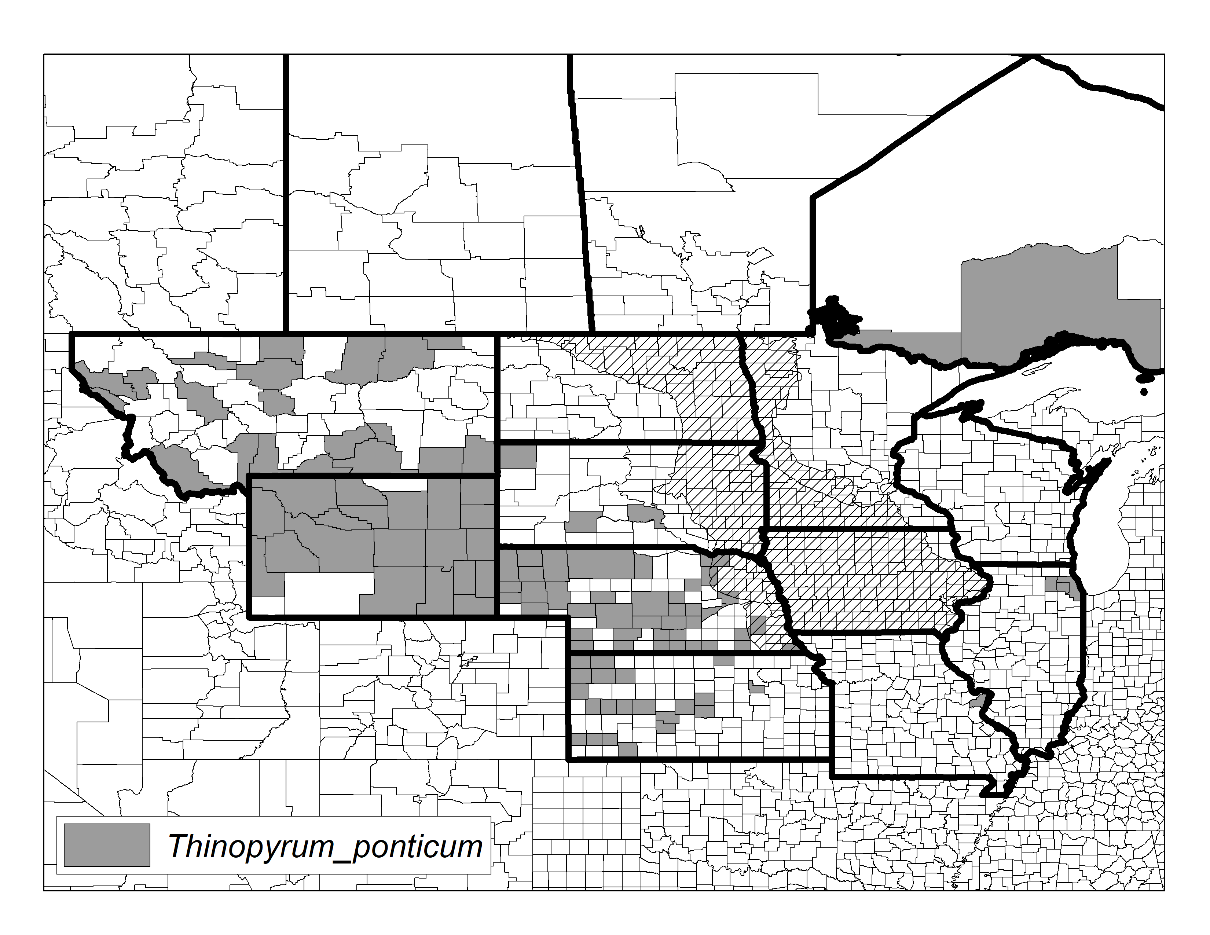

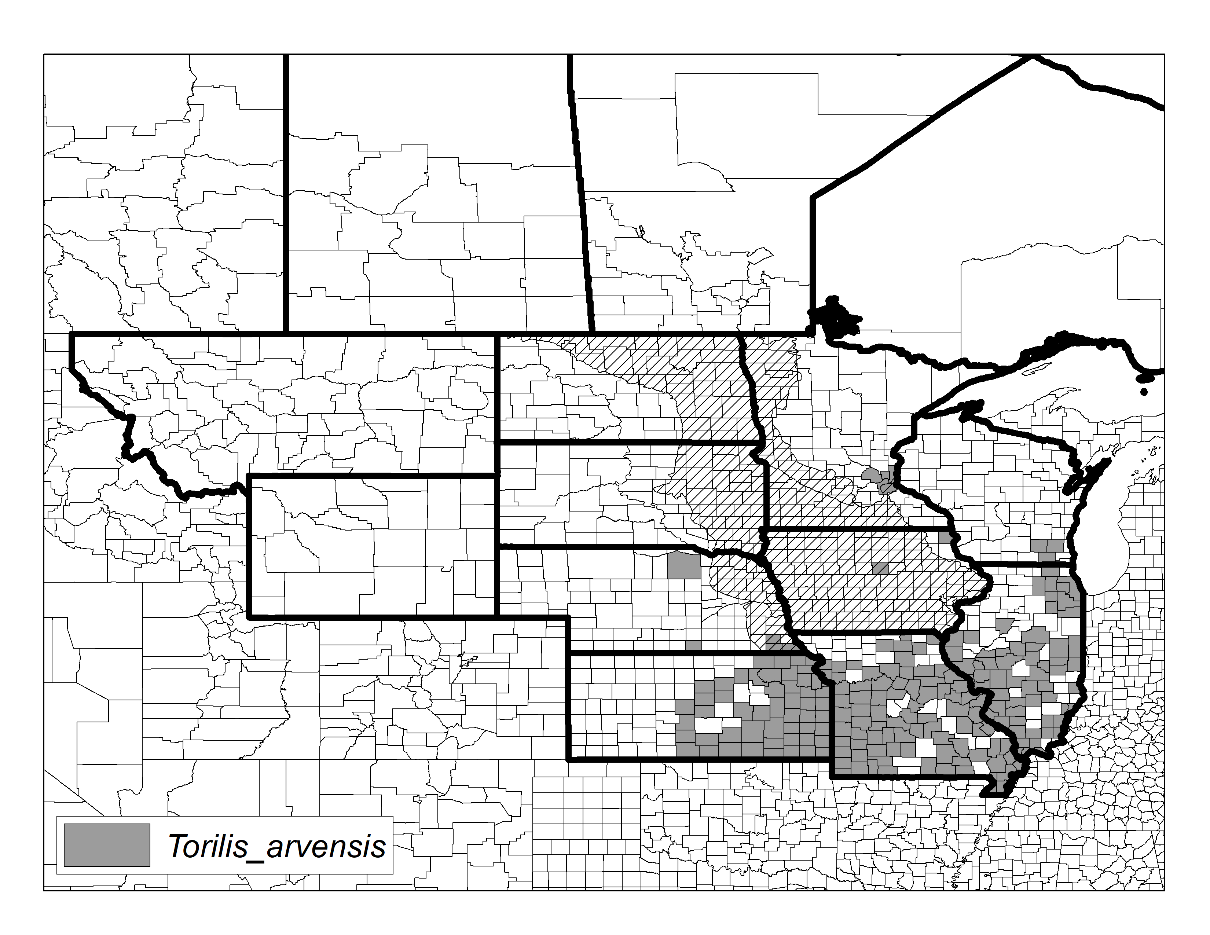


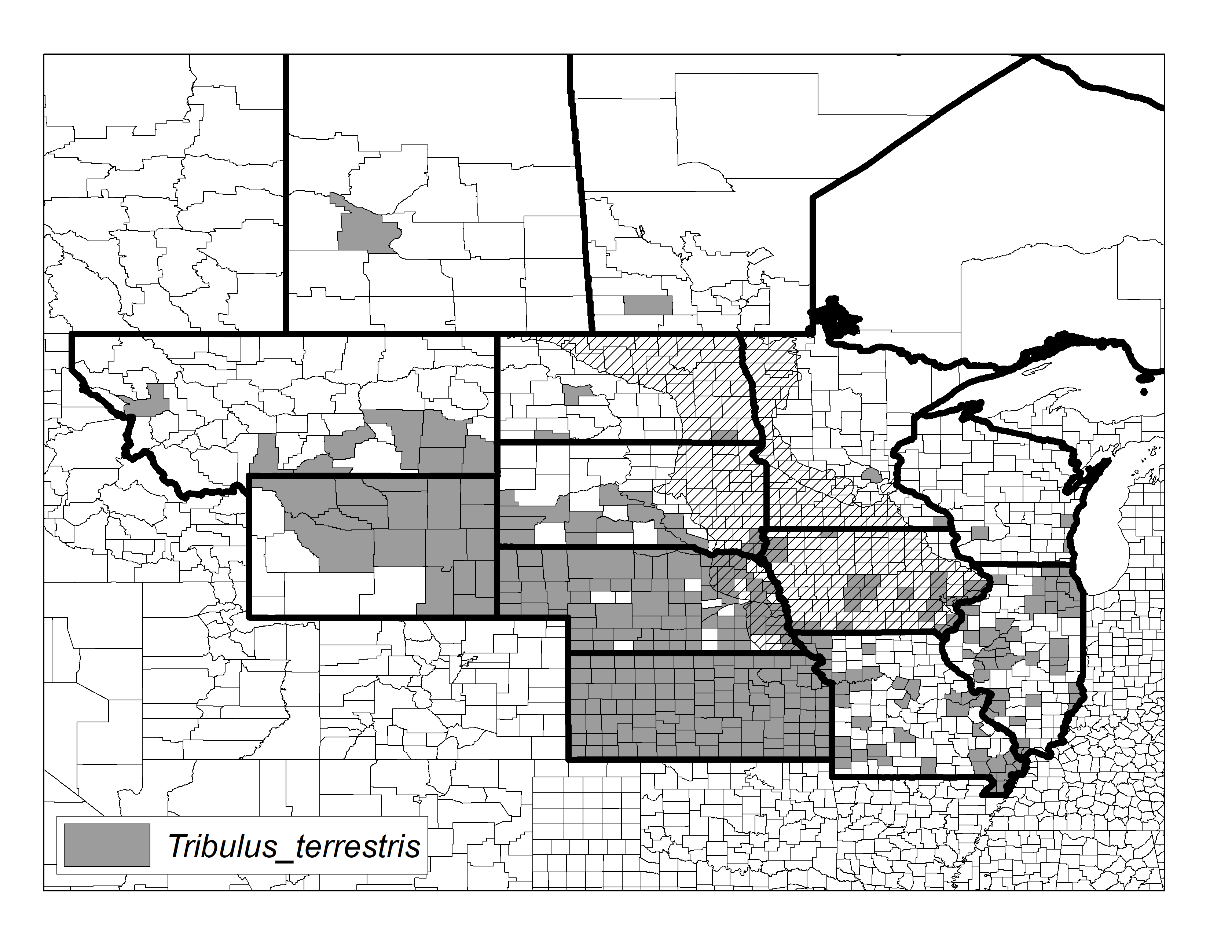

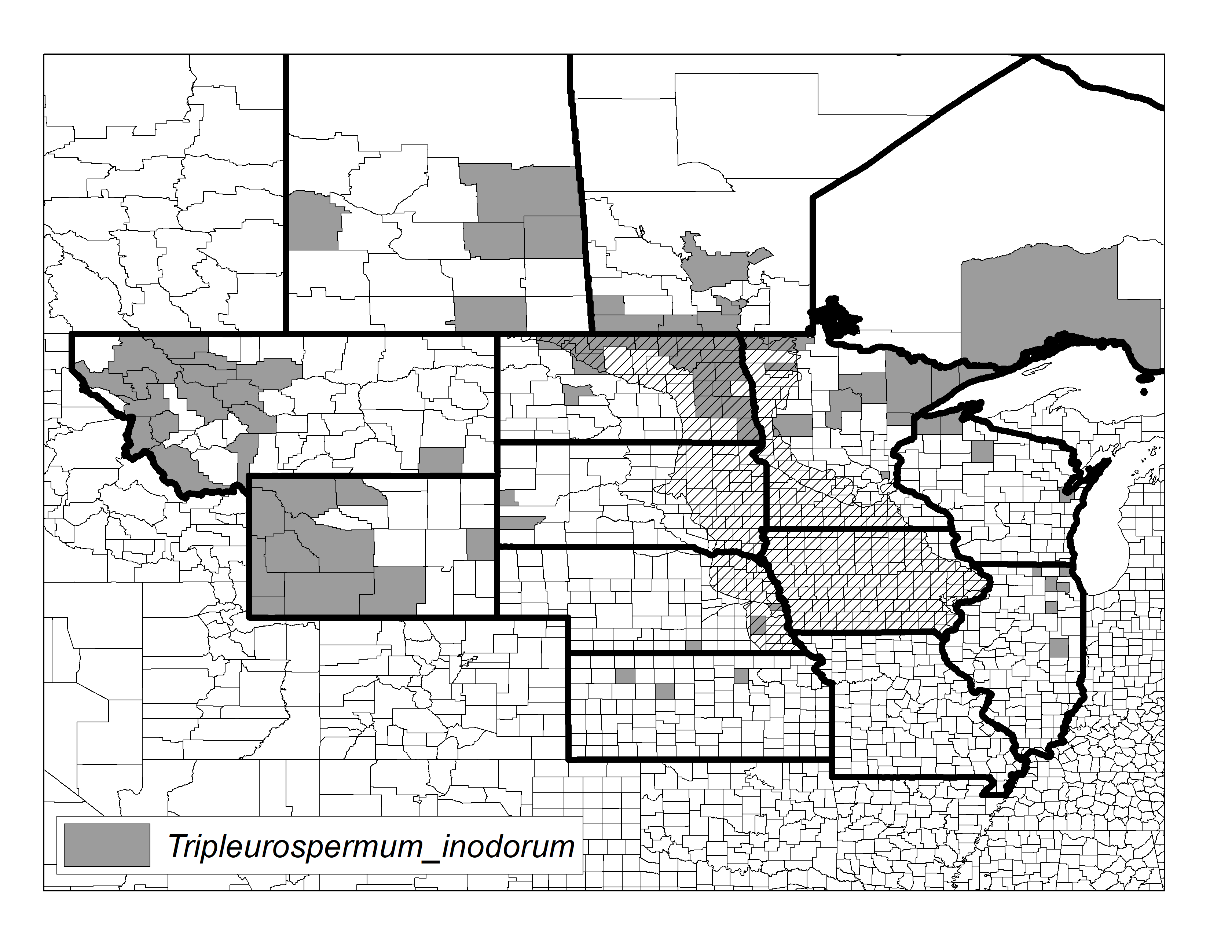

Supplement: S2 Appendix — Bold black lines are borders of the US states or Canadian provinces of our study’s focal region. Thin black lines are the borders of the counties (USA) or census tracts (Canada). The crosshatched area is the Level II Temperate Prairie Ecoregion within the upper Midwest, which was used to delineate our model’s sampling region. Darkened counties or census tracts are known occurrences of the species in each map. A complete list of references used to gather distribution data is provided in S3 Table. (DOCX) [file pone.0248583.s003.docx]
