# Supplementary material for: Balancing the need for seed against invasive species risks in prairie habitat restorations
Source: PLoS One. 2021 Apr 7;16(4):e0248583. doi: 10.1371/journal.pone.0248583 (PMC8026064; doi:10.1371/journal.pone.0248583)
Supplement: S1 Appendix — (DOCX) [file pone.0248583.s002.docx]

**S1 Appendix:** **Determining the number of ‘new’ exotic species needed per restoration site**

Preliminary data were analyzed part-way through data collection to assess sample size requirements for the exotic species distribution data set. These analyses were performed after all species from the state-level noxious weed or noxious weed seed lists (MN, ND, SD, NE & IA) were assessed for inclusion (see Table S2). To determine the minimum sample size of ‘new’ exotic species acceptable for analysis, we used a subset of sites with the maximum number of exotic species (45) and examined the safety regression after randomly removing intervals of 5 species. The figure below illustrates an example using one of these restoration sites:


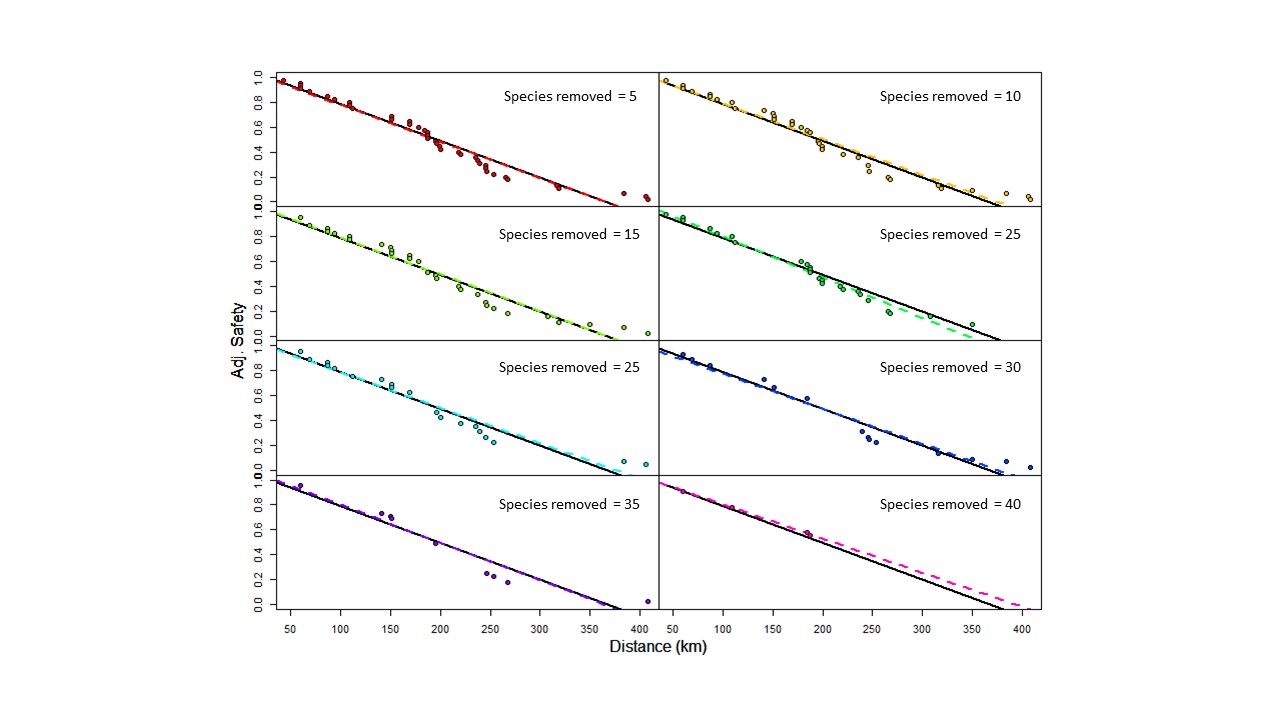
The solid black line is the regression with all 45 species included. Each box shows the number of species randomly removed in the upper right corner, and the dashed line is the new regression calculated with remaining species. Next, we ran a series of 100 iterations, randomly removing increments of 5 species up to 40. The following figure shows results of these iterations:


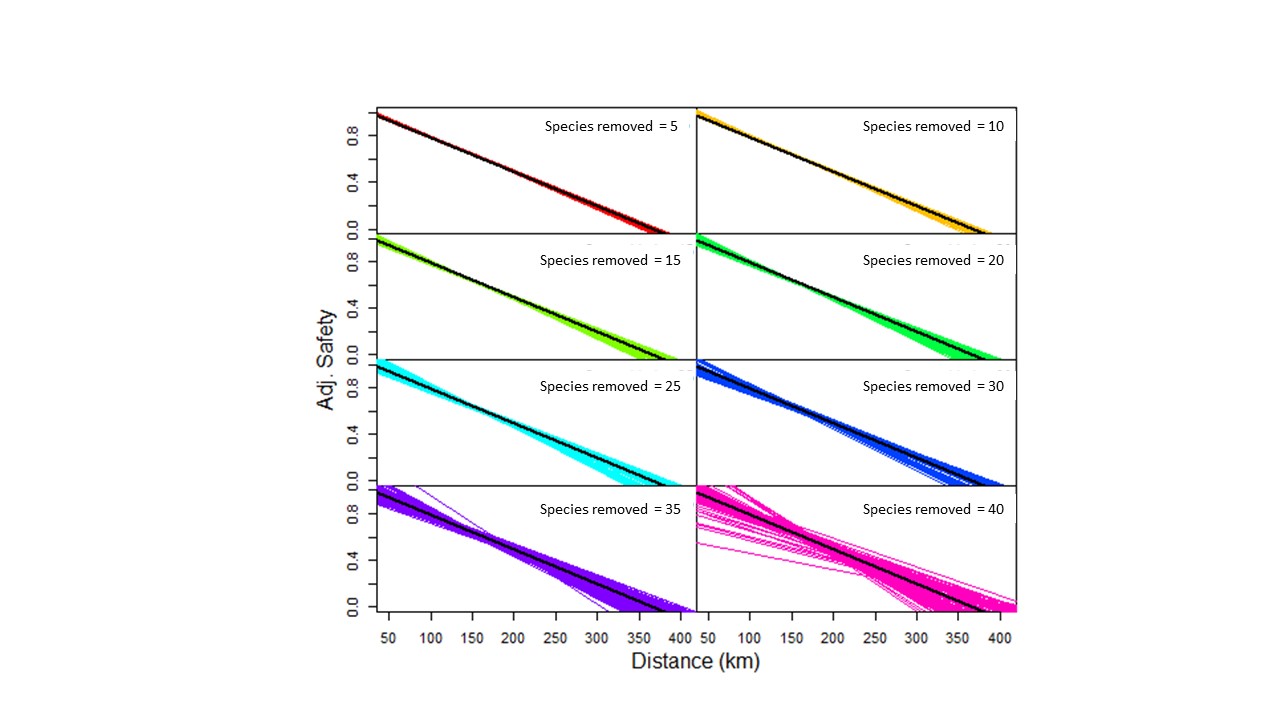


Again, the black line in each box is the regression with all 45 ‘new’ species included in the model. The number of species removed is indicated in the upper right corner of each box, and regression lines for each iteration in color surround the regression with the full complement of species. As each set of 5 species is removed, there is visible evidence of increased spread in the 100 iterations of each removal, which becomes especially obvious starting at the removal of 20 species (25 species remaining). We selected a cutoff point of 25 species because of this increased spread, and because of the natural cutoff of data at this point, as seen in the following histogram (red dotted line indicates 25 species):


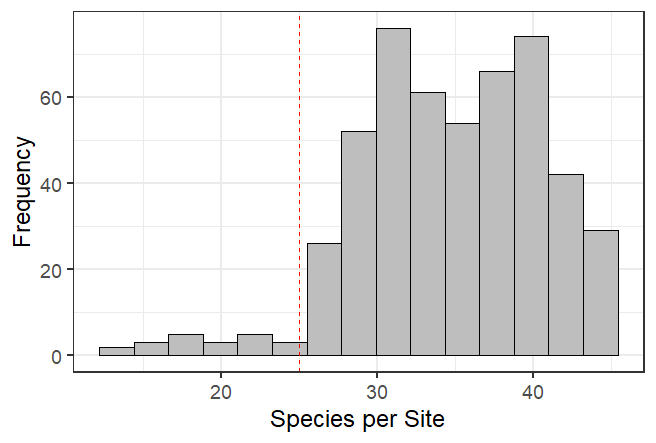


*Conclusion*

Taken together, these results suggest that a minimum sample size of 25 is enough to create a stable linear model. Thus, a minimum of 25 exotic species being ‘new’ to each restoration site was used in this study. After that criterion was set, data collection of exotic species distributions continued by assessing species listed as federal noxious weeds or having noxious weed seeds for inclusion (Table S2). After those species’ distribution assessments were complete, the minimum sample size requirement was met for 96.6% of the total 500 sites originally generated (483 sites). This was deemed adequate and the 17 restorations sites not meeting the 25 species minimum requirement were removed from the final analyses.
