# Supplementary material for: Balancing the need for seed against invasive species risks in prairie habitat restorations
Source: PLoS One. 2021 Apr 7;16(4):e0248583. doi: 10.1371/journal.pone.0248583 (PMC8026064; doi:10.1371/journal.pone.0248583)
Supplement: S1 Table — (DOCX) [file pone.0248583.s004.docx]

**S1 Table: National and regional websites, databases, reports and publications documenting commercial sources for native plant species’ propagation materials.**

| Source Title | Citation |
| --- | --- |
| National |  |
| National Suppliers Directory | National Suppliers Directory. Lady Bird Johnson Wildflower Center (2019). Available at: https://www.wildflower.org/suppliers/search.php |
| National Nursery and Seed Directory | USDA Forest Service & Southern Region Extension Forestry. National Nursery and Seed Directory. Reforestation, Nurseries & Genetic Resources (2019). Available at: https://rngr.net/resources/directory. |
| Native Seed Network | Native Seed Network. Institute for Applied Ecology (2019). Available at: http://nativeseednetwork.org/. |
| Native Plant Materials Directory | Native Plants Journal. 2015. 16(2): 126-200 |
| Plant Materials Centers | USDA & NRCS. Plant Materials Centers. (2019). Available at: https://www.nrcs.usda.gov/wps/portal/nrcs/detailfull/plantmaterials/pmc/?cid=stelprdb1041225. |
| Regional |  |
| Native Plant and Seed Providers | Midwest Invasive Plant Network. Native Plant and Seed Providers. Site Revegetation Resources (2018). Available at: https://www.mipn.org/cwma-resources/site-revegetation/ |
| Seed Certification: 'Where to Buy' | Minnesota Crop Improvement Association. Seed certification: Where to buy. Certification Services (2019). Available at: http://www.mncia.org/seed-certification. |
| Native Plant Suppliers and Landscapers in Minnesota | Minnesota Department of Natural Resources. Native plant suppliers and landscapers in Minnesota. Landscaping with native plants Available at: https://www.dnr.state.mn.us/gardens/nativeplants/suppliers.html |
| Wisconsin Native Plant Nurseries | Natural Heritage Conservation Program & Wisconsin Department of Natural Resources Wisconsin Native Plant Nurseries. (2019). Available at: https://dnr.wi.gov/files/pdf/pubs/er/er0698.pdf. |
| Plant Iowa Native | Plant Iowa Native. Native Plant Information. (2019). Available at: http://plantiowanative.com/resources/#information. |
