# Supplementary material for: Balancing the need for seed against invasive species risks in prairie habitat restorations
Source: PLoS One. 2021 Apr 7;16(4):e0248583. doi: 10.1371/journal.pone.0248583 (PMC8026064; doi:10.1371/journal.pone.0248583)
Supplement: S2 Table — (DOCX) [file pone.0248583.s005.docx]

S2 Table: Sources for state and federal noxious weed lists.

| Source Title | Citation |
| --- | --- |
| State Noxious Weeds |  |
| Minnesota Noxious Weed List | Minnesota Department of Agriculture. Minnesota 2018 Noxious Weed List. (2018). Available at: https://www.mda.state.mn.us/plants-insects/minnesota-noxious-weed-list. |
| North Dakota Noxious Weeds | North Dakota Department of Agriculture. North Dakota Noxious Weeds. (2018). Available at: https://www.nd.gov/ndda/plant-industries/noxious-weeds. |
| State Noxious Weed & Pest List | South Dakota Department of Agriculture. State Noxious Weed & Pest List. (2018). Available at: https://sdda.sd.gov/ag-services/weed-and-pest-control/weed-pest-control/sd-state-noxious-weed-declared-pest-list-and-distribution-maps/default.aspx. |
| Invasive Plant Species | Iowa Department of Natural Resources. Invasive Plant Species. (2018). Available at: https://www.iowadnr.gov/Conservation/Forestry/Forest-Health/Invasive-Plants. |
| Nebraska Invasive Species Program | University of Nebraska - Lincoln. Nebraska Invasive Species Program. (2018). Available at: https://neinvasives.com/species. |
| Federal Noxious Weeds |  |
| Federal Noxious Weeds | USDA & NRCS. Introduced, Invasive, and Noxious Plants (2019). The PLANTS Database. Available at: https://plants.usda.gov/java/noxious. |
| Noxious Weed Seeds |  |
| Federal Seed Act | USDA. State Noxious-Weed Seed Requirements Recognized in the Administration of the Federal Seed Act. (2018). Available at: https://www.ams.usda.gov/rules-regulations/fsa. |
