# Supplementary material for: Balancing the need for seed against invasive species risks in prairie habitat restorations
Source: PLoS One. 2021 Apr 7;16(4):e0248583. doi: 10.1371/journal.pone.0248583 (PMC8026064; doi:10.1371/journal.pone.0248583)
Supplement: S3 Table — (DOCX) [file pone.0248583.s006.docx]

S3 Table: Total list of species used in the analyses for this study; sources used to obtain exotic species occurrence data are listed in endnotes.

| **Species Name** | **Endnote** |
| --- | --- |
| *Aegilops cylindrica* | ^[[1]](#endnote-1),^^[[2]](#endnote-2),^^[[3]](#endnote-3),^^[[4]](#endnote-4),^^[[5]](#endnote-5),^^[[6]](#endnote-6)^ |
| *Allium vineale* | ^1,2,3^ |
| *Alopecurus arundinaceus* | ^2,3,4^ |
| *Anthriscus sylvestris* | ^1,2,3,4,^^[[7]](#endnote-7),^^[[8]](#endnote-8)^ |
| *Artemisia absinthium* | ^1,2,3,4,^^[[9]](#endnote-9),^^[[10]](#endnote-10),7,^^[[11]](#endnote-11)^ |
| *Bothriochloa bladhii* | ^1,2,3,4^ |
| *Bothriochloa ischaemum* | ^1,2,3,4,^^[[12]](#endnote-12)^ |
| *Centaurea diffusa* | ^1,2,3,4,^^[[13]](#endnote-13),^^[[14]](#endnote-14)^ |
| *Centaurea jacea* | ^1,2,3,4,5,10,^^[[15]](#endnote-15)^ |
| *Centaurea solstitialis* | ^1,2,3,4^ |
| *Centaurea x moncktonii* | ^1,3,4,^^[[16]](#endnote-16)^ |
| *Crepis tectorum* | ^1,2,3,4,5,10,^^[[17]](#endnote-17),^^[[18]](#endnote-18),^^[[19]](#endnote-19)^ |
| *Datura stramonium* | ^1,2,3,4,5,11,^^[[20]](#endnote-20)^ |
| *Digitalis lanata* | ^2,3,4,5,12,8^ |
| *Dipsacus fullonum* | ^1,2,3,4,^^[[21]](#endnote-21)^ |
| *Dipsacus laciniatus* | ^1,2,3,4,8^ |
| *Falcaria vulgaris* | ^1,2,3,5,11,^^[[22]](#endnote-22),^^[[23]](#endnote-23)^ |
| *Galega officinalis* | ^3^ |
| *Galium verum* | ^1,2,3,4,9^ |
| *Gypsophila paniculata* | ^1,2,3,4,5,9,7,8^ |
| *Halogeton glomeratus* | ^1,2,3^ |
| *Heracleum mantegazzianum* | ^1,3,8^ |
| *Hieracium aurantiacum* | ^1,2,3,4,5,12^ |
| *Hieracium caespitosum* | ^1,2,3,4,5,16^ |
| *Humulus japonicus* | ^1,2,3,4,7,8^ |
| *Hyoscyamus niger* | ^1,2,3,4^ |
| *Hypericum perforatum* | ^1,2,3,4,9^ |
| *Ipomoea purpurea* | ^1,2,3,4,5^ |
| *Jacobaea vulgaris* | ^1,2,3,13,10,16^ |
| *Lepidium appelianum* | ^1,2,3,^^[[24]](#endnote-24)^ |
| *Lepidium latifolium* | ^1,2,3,4,9,8^ |
| *Lespedeza cuneata* | ^1,2,3,4^ |
| *Linaria dalmatica* | ^1,2,3,9,^^[[25]](#endnote-25),13,^^[[26]](#endnote-26),^^[[27]](#endnote-27)^ |
| *Marrubium vulgare* | ^1,2,3,4^ |
| *Onopordum acanthium* | ^1,2,3,4,25,7,^^[[28]](#endnote-28)^ |
| *Plantago lanceolata* | ^1,2,3,4^ |
| *Raphanus raphanistrum* | ^1,2,3,4,5,7^ |
| *Rhaponticum repens* | ^1,2,3^ |
| *Schedonorus arundinaceus* | ^1,2,3,4,5^ |
| *Setaria faberi* | ^1,2,3,4^ |
| *Solanum carolinense* | ^2,3,4^ |
| *Sorghum bicolor* | ^1,2,3,4,5^ |
| *Sorghum halepense* | ^1,2,3,4,^^[[29]](#endnote-29),^^[[30]](#endnote-30),^^[[31]](#endnote-31)^ |
| *Thinopyrum ponticum* | ^1,2,3,4,9^ |
| *Torilis arvensis* | ^1,2,3,4,7,8^ |
| *Tribulus terrestris* | ^1,2,3,4,25^ |
| *Tripleurospermum inodorum* | ^1,2,3,4,5^ |

1. EDDMapS. 2019. Early Detection & Distribution Mapping System. The University of Georgia - Center for Invasive Species and Ecosystem Health. Available online at <http://www.eddmaps.org/>. [↑](#endnote-ref-1)
2. USDA & NRCS. 2019. The PLANTS Database (http://plants.usda.gov). National Plant Data Team, Greensboro, NC 27401-4901 USA. [↑](#endnote-ref-2)
3. Kartesz, J.T., The Biota of North America Program (BONAP). 2015. North American Plant Atlas. (http://bonap.net/napa). Chapel Hill, N.C. [maps generated from Kartesz, J.T. 2015. Floristic Synthesis of North America, Version 1.0. Biota of North America Program (BONAP). (in press)]. [↑](#endnote-ref-3)
4. Consortium of Midwest Herbaria. SEINet Network (2019). Available at: http://midwestherbaria.org/portal/index.php [↑](#endnote-ref-4)
5. Bell Museum. Minnesota Biodiversity Atlas. University of Minnesota (2019). Available at: <https://bellatlas.umn.edu/>. [↑](#endnote-ref-5)
6. Donald, W. W. & Zimdahl, R. L. Persistence, Germinability, and Distribution of Jointed Goatgrass (Aegilops cylindrica) Seed in Soil. Weed Sci. 35, 149–154 (1987). [↑](#endnote-ref-6)
7. Chatka, K. & Dziuk, P. M. Minnesota Wildflower. (2019). Available at: <https://www.minnesotawildflowers.info/>. [↑](#endnote-ref-7)
8. Wisconsin Department of Natural Resources. Invasive Species. (2019). Available at: <https://dnr.wi.gov/topic/Invasives/%0A>. [↑](#endnote-ref-8)
9. Brouillet L, Desmet P, Coursol F, Meades SJ, Favreau M, Anions M, Bélisle P, Gendreau C, Shorthouse D, and contributors (2010+). Database of Vascular Plants of Canada (VASCAN). Online at http://data.canadensys.net/vascan and http://www.gbif.org/dataset/3f8a1297-3259-4700-91fc-acc4170b27ce, released on 2010-12-10. doi: http://doi.org/10.3897/phytokeys.25.3100 [↑](#endnote-ref-9)
10. Minnesota Department of Natural Resources. The State of Minnesota Vascular Plant Checklist (MNTaxa). (2019). Available at: https://webapps15.dnr.state.mn.us/mntaxa/reports/index. [↑](#endnote-ref-10)
11. University of Wisconsin - Madison. Online Virtual Flora of Wisconsin. (2019). Available at: <http://wisflora.herbarium.wisc.edu/index.php%0A>. [↑](#endnote-ref-11)
12. Hilty, J. Illinois Wildflower. (2017). Available at: <https://www.illinoiswildflowers.info/index.htm%0A>. [↑](#endnote-ref-12)
13. Purdue University. Pest Tracker: Exotic Pest Reporting. (2019). Available at: <http://pest.ceris.purdue.edu/pests.php>. [↑](#endnote-ref-13)
14. Sheley, R. L., Jacobs, J. S. & Carpinelli, M. F. Distribution, Biology, and Management of Diffuse Knapweed (Centaurea diffusa) and Spotted Knapweed (Centaurea maculosa). Weed Sci. 12, 353–362 (1998). [↑](#endnote-ref-14)
15. Cowbrough, M. Noxious Weeds Profile - Knapweed spp. (2006). Ontario Ministry of Agriculture, Food and Rural Affairs. Available at: http://www.omafra.gov.on.ca/english/crops/facts/info_knapweed.htm#current. [↑](#endnote-ref-15)
16. Montana Weed Control Association. Invasive and Pest Species. Montana Field Guide (2019). Available at: <http://fieldguide.mt.gov/Invasives.aspx%0A>. [↑](#endnote-ref-16)
17. Illinois State Museum. Illinois State Museum Herbarium Collection. (2012). Available at: http://www.museum.state.il.us/ismdepts/botany/herbarium/database.html. [↑](#endnote-ref-17)
18. Najda, H. G., Darwent, A. L. & Hamilton, G. The Biolog of Canadian Weeds. 54. Crepis tectorum L. Can. J. Plant Sci. 62, 473–481 (1982). [↑](#endnote-ref-18)
19. Sumners, W. H. & Archibold, O. W. Exotic plant species in the southern boreal forest of Saskatchewan. For. Ecol. Manage. 251, 156–163 (2007). [↑](#endnote-ref-19)
20. Weaver, S. E. & Warwick, S. I. The Biology of Canadian Weeds. 64. Datura stramonium L. Can. J. Plant Sci. 64, 979–991 (1984). [↑](#endnote-ref-20)
21. Graeve, K. MN NWAC Risk Assessment Worksheet - Common Teasel. (2016). Minnesota Department of Agriculture. Available at: https://www.mda.state.mn.us/plants/pestmanagement/weedcontrol/noxiouslist/commonteasel [↑](#endnote-ref-21)
22. Piya, S., Nepal, M. P., Neupane, A., Larson, G. L. & Butler, J. L. Inferring introduction history and spread of Falcaria vulgaris Bernh. (Apiaceae) in the United States based on herbarium records. Proc. S. Dak. Acad. Sci. 91, 113–129 (2012). [↑](#endnote-ref-22)
23. Piya, S. Introduction history and population genetics of Falcaria vulgaris (Apiaceae) in the United States. (South Dakota State University, 2013). [↑](#endnote-ref-23)
24. Francis, A. & Warwick, S. I. The biology of Canadian weeds. 3. Lepidium draba L., L. chalepense L., L. appelianum Al-Shehbaz (updated). Can. J. Plant Sci. 6, 379–401 (2008). [↑](#endnote-ref-24)
25. South Dakota Department of Agriculture. Weed & Pest Control. (2019). Available at: https://sdda.sd.gov/ag-services/weed-and-pest-control/weed-pest-control/. [↑](#endnote-ref-25)
26. Lym, R. G. Identification and Control of Invasive and Troublesome Weeds in North Dakota. (2018). North Dakota State University. Available at: https://www.ag.ndsu.edu/publications/crops/identification-and-control-of-invasive-and-troublesome-weeds-in-north-dakota. [↑](#endnote-ref-26)
27. Vujnovic, K. & Wein, R. W. The biology of Canadian weeds. 106. Linaria dalmatica (l.) Mill. Can. J. Plant Sci. 77, 483–491 (1997). [↑](#endnote-ref-27)
28. Invasive Species Council of Manitoba. Scotch Thistle. (2019). Available at: http://invasivespeciesmanitoba.com/site/index.php?page=scotch-thistle. [↑](#endnote-ref-28)
29. Werle, R., Jhala, A. J., Yerka, M. K., Dille, J. A. & Lindquist, J. L. Distribution of Herbicide-Resistant Shattercane and Johnsongrass Populations in Sorghum Production Areas of Nebraska and Northern Kansas. Pest Interact. Agron. Syst. 108, 321–328 (2016). [↑](#endnote-ref-29)
30. Warwick, S. I. & Black, I. D. The biology of Canadian weeds. 61. Sorghum halepense (l.) Pers. Can. J. Plant Sci. 63, 997–1014 (1983). [↑](#endnote-ref-30)
31. Dunn, C. D., Stephenson, M. B. & Stubbendieck, J. Common Grasses of Nebraska. (2016). [↑](#endnote-ref-31)
